# Supplementary material for: Immunogenicity of a 20-Valent Pneumococcal Conjugate Vaccine Versus a 13-Valent Vaccine in Infants: A Systematic Review and Meta-Analysis
Source: Vaccines (Basel). 2025 Nov 12;13(11):1156. doi: 10.3390/vaccines13111156 (PMC12656742; doi:10.3390/vaccines13111156)
Supplement: Supplementary file 1 [file vaccines-13-01156-s001.zip › vaccines-3917547-supplementary.pdf]

SUPPLEMENTARY DATA

TABLE OF CONTENTS

Abreviations and acronyms 3

Table S1. Searching strategy 4

Table S2. Studies excluded after full text read with the reason for exclusion 6

Table S3. Summary of meta-analysis results of GMR and DP after primary series for shared and additional serotypes. 7

Table S4. Summary of meta-analysis results of GMR and DP after booster dose for shared and additional serotypes. 10

Table S5 . Summary of meta-analysis results of GMTs of OPA after primary series in PCV20 and PCV13 for shared and additional serotypes. 13

Table S6. Summary of meta-analysis results of OPA after booster dose in PCV20 and PCV13 for shared and additional serotypes. 17

Table S7. Quality Grading of Evidence 21

Figures of risk of bias (S1-S6) 32

Figures of sensivity analysis for each serotype (S7-S14) 38

Figure S7. Sensivity analysis for GMR after primary series. 38

Figure S8. Sensivity analysis for GMR after booster dose. 43

Figure S9. Sensivity analysis for DP after primary series. 48

Figure S10. Sensivity analysis for DP after booster dose. 52

Figure S11. Sensivity analysis of GMTs of OPA PCV20 after primary series. 56

Figure S12. Sensivity analysis of GMTs of OPA PCV20 after booster dose. 60

Figure S13. Sensivity analysis of GMTs of OPA PCV13 after primary series. 64

Figure S14. Sensivity analysis of GMTs of OPA PCV13 after booster dose. 68

Abreviations and acronyms

DP: difference (PCV20 - PCV13) in percentage of participants who achieved predefined antibody levels for each serotype.

GMR: geometric mean ratio (PCV20/PCV13) of serotype-specific pneumococcal anti-capsular pneumococcal antibodies.

GMTs of OPA: geometric mean titers of serotype-specific opsonophagocytic activity.

IgG: immunoglobulin G.

MeSH: Medical Subject Headings.

PCV: pneumococcal conjugate vaccine.

PCV20: 20-valent pneumococcal conjugate vaccine.

PCV13: 13-valent pneumococcal conjugate vaccine.

Table S1. Searching strategy

Database	Strategy	Results
PubMed	1. "child"[MeSH Terms] OR "child*"[Title/Abstract] OR "infant"[MeSH Terms] OR "infant*"[Title/Abstract] OR toddl*[Title/Abstract]	3,473,239
	2. “pneumococcal vaccines"[MeSH Terms] OR "vaccines, conjugate"[MeSH Terms] OR (("streptococcus pneumoniae"[MeSH Terms] OR "pneumo*"[Title/Abstract]) AND ("vaccin*"[Title/Abstract] OR "vaccines"[MeSH Terms]) AND ("conjugate"[Title/Abstract] OR "valent*"[Title/Abstract]))	14,642
	3. "immunoglobulins"[MeSH Terms] OR "Immunoglobulin G"[MeSH Terms] OR "IgG"[Title/Abstract] OR "immunoglobulin*"[Title/Abstract] OR "immunogenicity, vaccine"[MeSH Terms] OR "immunogenicit*"[Title/Abstract] OR "antigenicit*"[Title/Abstract]	1,159,759
	4. “PCV20"[Title/Abstract] OR "twenty-valent"[Title/Abstract] OR "20-valent"[Title/Abstract] OR "PCV13"[Title/Abstract] OR "thirteen-valent"[Title/Abstract] OR "13-valent"[Title/Abstract]	2,931
	5. “randomized controlled trial"[Publication Type] OR "randomized controlled trials as topic"[MeSH Terms] OR "randomi*ed"[Title/Abstract] OR "clinical trials as topic"[MeSH Terms] OR "clinical trial"[Publication Type] OR "trial*"[Title/Abstract]	2,266,481
	6. #1 AND #2 AND #3 AND #4 AND #5	192
Scopus	**TITLE-ABS-KEY**	
	1. child* OR infant* OR toddl*	4,669,173
	2. pneumo* AND vaccin* AND (conjugat* OR valent*)	10,598
	3. random* OR trial* OR meta-analysis	5,450,799
	4. PCV20 OR twenty-valent OR 20-valent OR PCV13 OR thirteen-valent OR 13-valent	3,116
	5. immunog* OR igG OR antigenicit*	1,063,882
	6. #1 AND #2 AND #3 AND #4 AND #5	229
Web of Science	**All Fields**	
	1. child* OR infant* OR toddl*	4,189,952
	2. pneumo* AND vaccin* AND (conjugat* OR valent*)	12,279
	3. random* OR trial*	4,089,473
	4. PCV20 OR twenty-valent OR 20-valent OR PCV13 OR thirteen-valent OR 13-valent	3,008
	5. immunog* OR igG OR antigenicit*	527,529
	6. #1 AND #2 AND #3 AND #4 AND #5	223

	Title Abstract Keyword	
**Cochrane Library**	1. (child* OR infant* OR toddl*)	264,041
	2. (pneumo* AND vaccin* AND (conjugat* OR valent*))	1,907
	3. (PCV20 OR "twenty-valent" OR "20-valent" OR PCV13 OR "thirteen-valent" OR "13-valent")	610
	4. (immunog* OR igG OR antigenicit*)	38,516
	5. (random* OR trial*)	2,220,251
	6. #1 AND #2 AND #3 AND #4 AND #5	266

	**Database ClinicalTrials.gov search strategy**	
**Condition or disease field**	(child OR infant OR toddler OR children OR infants OR toddler)	17
**Intervention/treatment field**	((pneumococcal AND (vaccine OR vaccines) AND (conjugate OR valent)) AND (randomised OR trial) AND ((PCV20 OR twenty-valent OR 20-valent) AND (PCV13 OR thirteen-valent OR 13-valent)) AND (immunogenicity OR igG OR antigenicity))	

Table S2. Studies excluded after full text read with the reason for exclusion

**Reference** (Author and year)	**Reason for exclusion**
Sabharwal et al, 2022	Study population
Essink et al, 2022	Study population
Cannon et al, 2021	Study population
Hurley et al, 2021	Study population
Klein et al, 2021	Study population
Thompson et al, 2019	Study population
Fitz-Patrick et al,2021	Study population
Meyer et al, 2024	Single-arm study
Kelly et al, 2024	Intervention and control not of interest
NCT05412030, 2024	Intervention and control not of interest
NCT05408429, 2022	Intervention and control not of interest
De Wals et al 2024	Non outcome of interest
Tamimiet al, 2023	Non outcome of interest

Table S3. Summary of meta-analysis results of GMR and DP after primary series for shared and additional serotypes.

		Primary series			
**Serotype**	**Author**	**GMR**	**CI 95%**	**DP**	**CI 95%**
Common					
1	Korbal, 2024	0,61	[0,54; 0,69]	-13,5	[-18,3; -8,7]
	Senders, 2024	0,65	[0,59; 0,72]	-8,6	[-12,1; -5,1]
	Ishihara, 2024	0,62	[0,54; 0,72]	-1,4	[-4,4; 1,3]
	Senders, 2021	0,79	[0,65; 0,96]	0,1	[-6,87; 7,07]
	**Overall**	**0,66**	**[0,55; 0,78]**	**-5,99**	**[-15,96; 3,97]**
3	Korbal, 2024	0,71	[0,64; 0,79]	-17,9	[-23,2; -12,4]
	Senders, 2024	0,7	[0,64; 0,76]	-15,5	[-20,1; -10,8]
	Ishihara, 2024	0,71	[0,63; 0,81]	-2,7	[-6,2; 0,1]
	Senders, 2021	0,77	[0,64; 0,92]	-10,3	[-19,82; -0,78]
	**Overall**	**0,71**	**[0,68; 0,75]**	**-11,46**	**[-22,66; -0,26]**
4	Korbal, 2024	0,6	[0,52; 0,69]	-11	[-16; -5,9]
	Senders, 2024	0,7	[0,63; 0,78]	-8,4	[-12; -4,9]
	Ishihara, 2024	0,6	[0,51; 0,7]	-2,3	[-5,6; 0,5]
	Senders, 2021	0,83	[0,66; 1,05]	-3,6	[-10,09; 2,89]
	**Overall**	**0,67**	**[0,53; 0,84]**	**-6,27**	**[-12,77; 0,22]**
5	Korbal, 2024	0,6	[0,52; 0,7]	-12,6	[-17,8; -7,2]
	Senders, 2024	0,69	[0,61; 0,77]	-4,3	[-7,8; -0,8]
	Ishihara, 2024	0,59	[0,49; 0,71]	-5	[-9,6; -0,9]
	Senders, 2021	0,82	[0,65; 1,04]	-2	[-8,72; 4,72]
	**Overall**	**0,66**	**[0,31; 0,83]**	**-6,01**	**[-13,05; 1,02]**
6A	Korbal, 2024	0,54	[0,45; 0,65]	-14,1	[-19,5; -8,6]
	Senders, 2024	0,72	[0,65; 0,81]	-2,4	[-4,6; -0,2]
	Ishihara, 2024	0,59	[0,5; 0,7]	-8,1	[-13; -4]
	Senders, 2021	0,89	[0,7; 1,12]	1,2	[-4,28; 6,68]
	**Overall**	**0,67**	**[0,48; 0,94]**	**-5,75**	**[-16,21; 4,71]**
6B	Korbal, 2024	0,51	[0,43; 0,61]	-15,8	[-21; -10,6]
	Senders, 2024	0,6	[0,51; 0,7]	-4,1	[-7; -1,2]
	Ishihara, 2024	0,51	[0,4; 0,64]	-8,6	[-14; -3,7]
	Senders, 2021	0,64	[0,45; 0,9]	-3,6	[-10,40; 3,20]
	**Overall**	**0,55**	**[0471; 0,65]**	**-8,00**	**[-16,944; 0,94]**
7F	Korbal, 2024	0,72	[0,64; 0,8]	-2,6	[-6,3; 1,1]
	Senders, 2024	0,75	[0,69; 0,81]	-1	[-2,7; 0,7]

	Ishihara, 2024	0,72	[0,62; 0,84]	-3,2	[-6,8; -0,3]
	Senders, 2021	0,83	[0,70; 0,98]	1	[-2,03; 4,03]
	**Overall**	**0,75**	**[0,69; 0,82]**	**-1,29**	**[-4,094; 1,50]**
	Korbal, 2024	0,59	[0,5; 0,69]	-14,3	[-19,7; -8,9]
	Senders, 2024	0,72	[0,65; 0,8]	-7,9	[-11,3; -4,6]
9V	Ishihara, 2024	0,69	[0,6; 0,8]	-2,7	[-6,4; 0,4]
	Senders, 2021	0,84	[0,68; 1,05]	0,1	[-6,48; 6,68]
	**Overall**	**0,70**	**[0,56; 0,87]**	**-6,27**	**[-16,08; 3,53]**
	Korbal, 2024	0,82	[0,7; 0,96]	-3,3	[-7,9; 1,4]
	Senders, 2024	0,79	[0,71; 0,89]	-0,8	[-3,1; 1,6]
14	Ishihara, 2024	0,84	[0,7; 1,01]	-0,9	[-4,4; 2,4]
	Senders, 2021	0,88	[0,70; 1,10]	-1,50	[-6,35; 3,35]
	**Overall**	**0,82**	**[0,77; 0,87]**	**-1,26**	**[-2,82; 0,30]**
	Korbal, 2024	0,79	[0,67; 0,92]	-5,5	[-10,6; -0,4]
	Senders, 2024	0,77	[0,7; 0,84]	-0,6	[-3,1; 1,9]
18C	Ishihara, 2024	0,66	[0,57; 0,77]	-2,3	[-5,6; 0,5]
	Senders, 2021	0,78	[0,63; 0,96]	-2,6	[-7,82; 2,62]
	**Overall**	**0,75**	**[0,66; 0,85]**	**-2,17**	**[-5,18; 0,83]**
	Korbal, 2024	0,59	[0,51; 0,69]	-1,7	[-4,8; 1,3]
	Senders, 2024	0,79	[0,72; 0,86]	-1	[-2,6; 0,5]
19A	Ishihara, 2024	0,76	[0,65; 0,87]	0	[-2,1; 2,1]
	Senders, 2021	0,83	[0,69; 1,01]	0,5	[-2,72; 3,72]
	**Overall**	**0,74**	**[0,58; 0,94]**	**-0,63**	**[-1,93; 0,68]**
	Korbal, 2024	0,72	[0,64; 0,82]	-1,4	[-4; 1,2]
	Senders, 2024	0,79	[0,73; 0,86]	0,2	[-1,5; 2]
19F	Ishihara, 2024	0,75	[0,67; 0,85]	0	[-1,7; 1,7]
	Senders, 2021	0,87	[0,73; 1,04]	1,6	[-1,97; 5,17]
	**Overall**	**0,77**	**[0691; 0,86]**	**-0,02**	**[-1,54; 1,50]**
	Korbal, 2024	0,52	[0,44; 0,62]	-18,3	[-23,6; -12,9]
	Senders, 2024	0,66	[0,58; 0,75]	-7,6	[-11,4; -3,9]
23F	Ishihara, 2024	0,64	[0,53; 0,78]	-4	[-9,5; 1,2]
	Senders, 2021	0,75	[0,56; 0,99]	-1,9	[-10,21; 6,41]
	**Overall**	**0,63**	**[0,57; 0,77]**	**-5,47**	**[-12,36; 1,41]**
Additional					
	Korbal, 2024	26,55	[22,98; 30,67]	59,9	[55,6; 64,1]
	Senders, 2024	1,98	[1,81; 2,16]	11,2	[8,6; 14]
8	Senders, 2021	4,01	[3, 36; 4,79]	5,9	[3; 10]
	Senders, 2021	2,11	[1,62; 2,76]	17,7	[11,72; 23,68]

	**Overall**	**4,60**	**[0,67; 31,69]**	**23,66**	**[-15,54; 62,86]**
	Korbal, 2024	2,67	[2,25; 3,17]	-7,6	[-13,1; -2,1]
	Senders, 2024	1,32	[1,18; 1,49]	-3,3	[-6,9; 0,3]
10A	Ishihara, 2024	0,6	[0,48; 0,76]	-33,5	[-40,7; -26,2]
	Senders, 2021	1,69	[1,21; 2,35]	6	[-1,59; 13,59]
	**Overall**	**1,37**	**[0,51; 3,72]**	**-9,55**	**[-36,29; 17,19]**
	Korbal, 2024	26,6	[22,95; 30,82]	57,6	[53,1; 61,9]
11A	Senders, 2024	1,52	[1,39; 1,67]	7,1	[4,2; 10,2]
	Ishihara, 2024	6,8	[5,69; 8,13]	6,4	[3,8; 10,4]
	Senders, 2021	1,96	[1,48; 2,60]	15,16	[9,24; 21,96]
	**Overall**	**4,82**	**[0,60; 39,07]**	**21,66**	**[-17,05; 60,36]**
	Korbal, 2024	2,48	[2,08; 2,97]	-6,2	[-11,7; -0,7]
	Senders, 2024	0,6	[0,54; 0,67]	-18,1	[-22,1; -14]
12F	Ishihara, 2024	0,95	[0,76; 1,2]	-19	[-25,7; -12,5]
	Senders, 2021	0,87	[0,64; 1,18]	0,7	[-7,40; 8,80]
	**Overall**	**1,05**	**[0,40; 2,77]**	**-10,93**	**[-25,94; 4,08]**
	Korbal, 2024	54,6	[46,35; 64,3]	57,8	[53,3; 62,1]
	Senders, 2024	4,82	[4,39; 5,3]	12,7	[10,2; 15,4]
15B	Ishihara, 2024	8,18	[6,75; 9,92]	5,5	[2,2; 9,6]
	Senders, 2021	5,92	[4,45; 7,87]	17,1	[11,01; 23,19]
	**Overall**	**10,64**	**[0,51; 0,79]**	**23,26**	**[-14,16; 60,68]**
	Korbal, 2024	36,8	[31,57; 42,89]	57,8	[53,3; 62,1]
	Senders, 2024	4,06	[3,68; 4,48]	12,8	[10,3; 15,5]
22F	Ishihara, 2024	5,97	[5; 7,12]	6,4	[3,8; 10,4]
	Senders, 2021	4,67	[3,49; 6,24]	17,1	[11,01; 23,19]
	**Overall**	**8,04**	**[1,57; 41,32]**	**23,50**	**[-13,53; 60,54]**
	Korbal, 2024	5,03	[4,27; 5,92]	10,3	[4,5; 16]
	Senders, 2024	1,64	[1,46; 1,83]	1,1	[-2,2; 4,5]
33F	Ishihara, 2024	2,06	[1,69; 2,51]	1,3	[-3,2; 6]
	Senders, 2021	2,23	[1,65; 3,02]	10,3	[3,17; 17,43]
	**Overall**	**2,48**	**[1,13; 5,44]**	**5,21**	**[-3,10; 13,51]**

GMR: geometric mean ratio; DP: difference in percentage; CI: confidence interval.

Table S4. Summary of meta-analysis results of GMR and DP after booster dose for shared and additional serotypes.

		Booster dose			
**Serotype**	**Author**	**GMR**	**CI 95%**	**DP**	**CI 95%**
Common					
1	Korbal, 2024	0,67	[0,6; 0,75]	-1,92	[-3,1; 0,9]
	Senders, 2024	0,69	[0,63; 0,76]	-2,9	[-5; -0,8]
	Ishihara, 2024	0,60	[0,51; 0,71]		
	Senders, 2021	0,73	[0,61; 0,87]		
	**Overall**	**0,67**	**[0,60; 0,75]**	**-1,92**	**[-13,99; 10,14]**
3	Korbal, 2024	0,66	[0,59; 0,73]	-10,6	[-14,72; -6,7]
	Senders, 2024	0,66	[0,61; 0,73]	-12,1	[-16,21; -8,1]
	Ishihara, 2024	0,67	[0,58; 0,78]		
	Senders, 2021	0,77	[0,62; 0,97]		
	**Overall**	**0,67**	**[0,62; 0,73]**	**-11,34**	**[-20,87; -1,81]**
4	Korbal, 2024	0,77	[0,68; 0,87]	0	[-1,4; 1,3]
	Senders, 2024	0,78	[0,7; 0,86]	-0,1	[-1,3; 1]
	Ishihara, 2024	0,69	[0,58; 0,82]		
	Senders, 2021	0,76	[0,62; 0,93]		
	**Overall**	**0,76**	**[0,70; 0,82]**	**-0,06**	**[-0,69; 0,57]**
5	Korbal, 2024	0,72	[0,64; 0,81]	0,4	[-1,4; 2,2]
	Senders, 2024	0,74	[0,67; 0,82]	0,2	[-1,4; 1,7]
	Ishihara, 2024	0,64	[0,54; 0,75]		
	Senders, 2021	0,69	[0,56; 0,84]		
	**Overall**	**0,71**	**[0,64; 0,78]**	**0,29**	**[-0,97; 1,54]**
6A	Korbal, 2024	0,66	[0,57; 0,75]	0	[-1,6; 1,5]
	Senders, 2024	0,77	[0,7; 0,85]	-0,3	[-1,1; 0,5
	Ishihara, 2024	0,69	[0,59; 0,80]		
	Senders, 2021	0,72	[0,61; 0,88]		
	**Overall**	**0,72**	**[0,64; 0,85]**	**-0,24**	**[-1,79; 1,32]**
6B	Korbal, 2024	0,57	[0,48; 0,67]	0,8	[-1,1; 2,7]
	Senders, 2024	0,7	[0,62; 0,79]	-0,4	[-1,4; 0,6]
	Ishihara, 2024	0,69	[0,58; 0,81]		
	Senders, 2021	0,65	[0,51; 0,83]		
	**Overall**	**0,66**	**[0,56; 0,76]**	**-0,03**	**[-7,05; 6,98]**
7F	Korbal, 2024	0,73	[0,67; 0,8]	-0,4	[-1,5; 0,4]

	Senders, 2024	0,76	[0,7; 0,82]	-0,4	[-1,2; 0,3]
	Ishihara, 2024	0,67	[0,58; 0,78]		
	Senders, 2021	0,66	[0,56; 0,77]		
	**Overall**	**0,72**	**[0,65; 0,80]**	**-0,39**	**[-0,46; -033]**
9V	Korbal, 2024	0,73	[0,66; 0,81]	0,4	[-1; 1,9]
	Senders, 2024	0,8	[0,73; 0,88]	-0,4	[-1,6; -0,8]
	Ishihara, 2024	0,69	[0,58; 0,80]		
	Senders, 2021	0,71	[0,58; 0,86]		
	**Overall**	**0,74**	**[0,67; 0,83]**	**-0,06**	**[-5,08; 4,95]**
14	Korbal, 2024	0,8	[0,69; 0,92]	-1,5	[-3,7; 0,6]
	Senders, 2024	0,9	[0,81; 1]	-0,5	[-1,6; 0,4]
	Ishihara, 2024	0,80	[0,68; 0,94]		
	Senders, 2021	0,78	[0,62; 0,98]		
	**Overall**	**0,84**	**[0,75; 0,94]**	**-0,73**	**[-6,04; 4,59]**
18C	Korbal, 2024	0,75	[0,67; 0,84]	1	[-0,5; 2,7]
	Senders, 2024	0,74	[0,67; 0,82]	0,3	[-0,9; 1,5]
	Ishihara, 2024	0,66	[0,56; 0,79]		
	Senders, 2021	0,66	[0,54; 0,81]		
	**Overall**	**0,72**	**[0,65; 0,79]**	**0,56**	**[-3,74; 4,86]**
19A	Korbal, 2024	0,82	[0,72; 0,93]	0	[-1,1; 1,1]
	Senders, 2024	0,85	[0,77; 0,94]	0,1	[-0,5; 0,9]
	Ishihara, 2024	0,85	[0,73; 0,99]		
	Senders, 2021	0,81	[0,65; 1,01]		
	**Overall**	**0,84**	**[0,81; 0,87]**	**0,07**	**[-0,50; 0,65]**
19F	Korbal, 2024	0,77	[0,68; 0,87]	0,2	[-0,9; 1,4]
	Senders, 2024	0,86	[0,78; 0,96]	-0,1	[-1,3; 1,1]
	Ishihara, 2024	0,79	[0,68; 0,92]		
	Senders, 2021	0,84	[0,68; 1,03]		
	**Overall**	**0,82**	**[0,75; 0,89]**	**0,06**	**[-1,85; 1,96]**
23F	Korbal, 2024	0,6	[0,52; 0,69]	-0,9	[-3,2; 1,4]
	Senders, 2024	0,64	[0,57; 0,72]	-0,9	[-2,5; 0,7]
	Ishihara, 2024	0,60	[0,50; 0,71]		
	Senders, 2021	0,62	[0,48; 0,79]		
	**Overall**	**0,62**	**[0,58; 0,65]**	**-0,87**	**[-1,46; -0,28]**
Additional					
8	Korbal, 2024	1,48	[1,32; 1,66]	2	[0,4; 3,9]
	Senders, 2024	1,87	[1,71; 2,06]	2,3	[1,1; 3,8]

	Ishihara, 2024	1,26	[1,07; 1,48]		
	Senders, 2021	0,86	[0,73; 1,02]		
	**Overall**	**1,32**	**[0,79; 2,22]**	**2,19**	**[0,34; 4,03]**
10A	Korbal, 2024	2,02	[1,77; 2,3]	0,6	[-1,5; 2,7]
	Senders, 2024	2,94	[2,64; 3,26]	0,6	[-1,1; 2,3]
	Ishihara, 2024	1,50	[1,26; 1,79]		
	Senders, 2021	2,74	[2,26; 3,32]		
	**Overall**	**2,23**	**[1,37; 3,63]**	**0,60**	**[0,53; 0,66]**
11A	Korbal, 2024	1,55	[1,37; 1,75]	1,2	[-0,7; 3,2]
	Senders, 2024	1,67	[1,51; 1,84]	1,6	[0,2; 3,2]
	Ishihara, 2024	1,23	[1,04; 1,46]		
	Senders, 2021	1,57	[1,30; 1,89]		
	**Overall**	**1,51**	**[1,23; 1,86]**	**1,45**	**[-1,01; 3,91]**
12F	Korbal, 2024	0,77	[0,68; 0,87]	-0,6	[-2,9; 1,6]
	Senders, 2024	0,88	[0,79; 0,97]	-1,9	[1,4; 4]
	Ishihara, 2024	0,59	[0,50; 0,70]		
	Senders, 2021	0,53	[0,44; 0,64]		
	**Overall**	**0,68**	**[0,47; 0,99]**	**-1,5**	**[-9,13; 6,14]**
15B	Korbal, 2024	5,42	[4,82; 6,1]	2,2	[0,7; 4,1]
	Senders, 2024	5,95	[5,39; 6,55]	2,6	[1,4; 4]
	Ishihara, 2024	3,98	[3,42; 4,63]		
	Senders, 2021	5,08	[4,29; 6,03]		
	**Overall**	**5,09**	**[3,87; 6,69]**	**2,45**	**[-0,002; 4,91]**
22F	Korbal, 2024	3,84	[3,4; 4,34]	2	[0,4; 3,9]
	Senders, 2024	5,01	[4,54; 5,52]	2,4	[1,3; 3,9]
	Ishihara, 2024	3,03	[2,59; 3,55]		
	Senders, 2021	4,04	[3,32; 4,92]		
	**Overall**	**3,94**	**[2,83; 5,49]**	**2,26**	**[-0,18; 4,69]**
33F	Korbal, 2024	2,64	[2,33; 2,99]	1,4	[-0,4; 3,4]
	Senders, 2024	4,4	[3,99; 4,85]	2,3	[1,1; 3,8]
	Ishihara, 2024	2,22	[1,90; 2,59]		
	Senders, 2021	1,29	[1,09; 1,53]		
	**Overall**	**2,41**	**[1,08; 5,38]**	**1,98**	**[-3,49; 7,45]**

GMR: geometric mean ratio; DP: difference in percentage; CI: confidence interval.

Table S5 . Summary of meta-analysis results of GMTs of OPA after primary series in PCV20 and PCV13 for shared and additional serotypes.

Serotype	Author	Primary series			
		PCV20		PCV13	
		GMTs of OPA	CI 95%	GMTs of OPA	CI 95%
**Common**					
1	Korbal, 2024	14	[12; 16]	23	[19; 28]
	Senders, 2024	26	[21; 33]	34	[27; 42]
	Ishihara, 2024	56	[45; 70]	126	[104; 152]
	Senders, 2021	16,3	[12,8; 20,8]	31,3	[22,8; 43]
	**Overall**	**23,96**	**[8,89; 64,61]**	**42,01**	**[12,62; 139,82]**
	**Schedule 3+1**	**28,78**	**[6,12; 135,30]**	**51,45**	**[7,35; 359,99]**
	**Schedule 2+1**	**14**	**[12; 16]**	**23**	**[19; 28]**
3	Korbal, 2024	31	[26; 36]	40	[34; 47]
	Senders, 2024	51	[43; 61]	63	[53; 76]
	Ishihara, 2024	120	[103; 139]	170	[147; 196]
	Senders, 2021	50,2	[39; 64,6]	61,8	[50,1; 76,2]
	**Overall**	**55,63**	**[22,62; 136,84]**	**71,83**	**[27,04; 190,81]**
	**Schedule 3+1**	**67,82**	**[19,55; 235,23]**	**87,42**	**[20,69; 369,29]**
	**Schedule 2+1**	**31**	**[26; 36]**	**40**	**[34; 47]**
4	Korbal, 2024	333	[270; 413]	391	[314; 486]
	Senders, 2024	339	[252; 455]	280	[207; 378]
	Ishihara, 2024	1617	[1298; 2015]	1768	[1393; 2245]
	Senders, 2021	424,5	[296,2; 608,2]	390,3	[239,5; 636]
	**Overall**	**529,61**	**[158,37; 1771,09]**	**527,81**	**[140,64; 1980,79]**
	**Schedule 3+1**	**619,11**	**[75,31; 5089,49]**	**583,10**	**[50,05; 6793,71]**
	**Schedule 2+1**	**333**	**[270; 413]**	**391**	**[314; 486]**
5	Korbal, 2024	21	[18; 23]	27	[23; 31]
	Senders, 2024	32	[27; 39]	39	[32; 47]
	Ishihara, 2024	91	[77; 109]	154	[131; 183]
	Senders, 2021	32,4	[273; 441]	45,2	[35,9; 57]
	**Overall**	**37,5**	**[13,85; 101,54]**	**52,05**	**[15,62; 173,45]**
	**Schedule 3+1**	**45,67**	**[10,26; 203,35]**	**64,89**	**[9,94; 423,43]**
	**Schedule 2+1**	**21**	**[18; 23]**	**27**	**[23; 31]**
6A	Korbal, 2024	347	[273; 441]	409	[318; 527]
	Senders, 2024	910	[763; 1084]	936	[757; 1156]
	Ishihara, 2024	3120	[2562; 3799]	3339	[2777; 4013]
	Senders, 2021	817	[590,3; 1130,7]	866,8	[690,3; 1088,6]
	**Overall**	**949,33**	**[224,62; 4012,18]**	**1028,76**	**[257,14; 4115,91]**
	**Schedule 3+1**	**1329,77**	**[208,65; 8474,81]**	**1396,77**	**[212,38; 9185,97]**
	**Schedule 2+1**	**347**	**[273; 441]**	**409**	**[318; 527]**

6B	Korbal, 2024	54	[42; 71]	105	[76; 144]
	Senders, 2024	318	[242; 419]	516	[409; 651]
	Ishihara, 2024	1563	[1215; 2010]	2489	[2005; 3088]
	Senders, 2021	432,6	[287; 652,2]	668,8	[474,8; 942,2]
	**Overall**	**328,06**	**[35,97; 2992,36]**	**549,67**	**[69,52; 4346,33]**
	**Schedule 3+1**	**602,14**	**[73,10; 4959,82]**	**953,99**	**[116,53; 7810,11]**
	**Schedule 2+1**	**54**	**[42; 71]**	**105**	**[76; 144]**
7F	Korbal, 2024	858	[736; 1000]	895	[781; 1027]
	Senders, 2024	1222	[1020; 1465]	1149	[926; 1424]
	Ishihara, 2024	4491	[3675; 5490]	4428	[3821; 5131]
	Senders, 2021	1480,4	[1155,5; 1896,6]	1390,7	[1040; 1858,4]
	**Overall**	**1622,59**	**[520; 5063,03]**	**1589,29**	**[512,52; 4928,3]**
	**Schedule 3+1**	**2011,26**	**[350,36; 11545,86]**	**1930,09**	**[312,45; 11922,52]**
	**Schedule 2+1**	**858**	**[736; 1000]**	**895**	**[781; 1027]**
9V	Korbal, 2024	233	[182; 298]	285	[228; 358]
	Senders, 2024	661	[482; 906]	594	[421; 838]
	Ishihara, 2024	1929	[1554; 2394]	2388	[1986; 2870]
	Senders, 2021	522,4	[359,1; 7598]	601,4	[400,6; 902,7]
	**Overall**	**642,97**	**[148,08; 2791,87]**	**705,09**	**[169,79; 2928,03]**
	**Schedule 3+1**	**1003,17**	**[177,91; 5656,35]**	**961,41**	**[129,99; 7110,55]**
	**Schedule 2+1**	**233**	**[182; 298]**	**285**	**[228; 358]**
14	Korbal, 2024	287	[215; 383]	360	[264; 489]
	Senders, 2024	415	[323; 535]	420	[330; 535]
	Ishihara, 2024	2103	[1527; 2897]	2593	[1999; 3362]
	Senders, 2021	606,9	[400,9; 918,7]	456,9	[310; 673,4]
	**Overall**	**622,82**	**[156,77; 2474,29]**	**652,61**	**[148,67; 2864,62]**
	**Schedule 3+1**	**808,42**	**[97,49; 6703,62]**	**795,00**	**[61,64; 10252,95]**
	**Schedule 2+1**	**287**	**[215; 383]**	**360**	**[264; 489]**
18C	Korbal, 2024	588	[467; 741]	719	[590; 876]
	Senders, 2024	1153	[910; 1460]	996	[754; 1317]
	Ishihara, 2024	4908	[4037; 5967]	5355	[4617; 6212]
	Senders, 2021	1218,6	[948,2; 1566,2]	1491,7	[1070,7; 2078,2]
	**Overall**	**1421,46**	**[343,57; 5881,01]**	**1551,85**	**[379,70; 6342,47]**
	**Schedule 3+1**	**1908,34**	**[247,70; 14702,47]**	**2010,97**	**[224,94; 17977,98]**
	**Schedule 2+1**	**588**	**[467; 741]**	**719**	**[590; 876]**
19A	Korbal, 2024	57	[43; 75]	91	[69; 121]
	Senders, 2024	108	[78; 149]	109	[79; 151]
	Ishihara, 2024	553	[441; 693]	676	[551; 830]
	Senders, 2021	105,3	[73,2; 151,4]	157,2	[105,1; 235,3]
	**Overall**	**138,15**	**[29,17; 654,17]**	**181,23**	**[42,18; 778,61]**
	**Schedule 3+1**	**185,92**	**[17,41; 1985,40]**	**228,18**	**[20,50; 2539,44]**
	**Schedule 2+1**	**57**	**[43; 75]**	**91**	**[69; 121]**
19F	Korbal, 2024	97	[81; 116]	117	[94; 146]
	Senders, 2024	84	[67; 105]	116	[90; 149]
	Ishihara, 2024	488	[411; 580]	624	[494; 788]

	Senders, 2021	90,9	[66,2; 124,8]	121,2	[91,3; 160,9]
	**Overall**	**138,39**	**[35,96; 532,64]**	**179,18**	**[47,56; 675,03]**
	**Schedule 3+1**	**155,72**	**[13,12; 1848,65]**	**206,61**	**[19,04; 2242,22]**
	**Schedule 2+1**	**97**	**[81; 116]**	**117**	**[94; 146]**
23F	Korbal, 2024	59	[42; 84]	68	[48; 96]
	Senders, 2024	255	[186; 350]	295	[215; 406]
	Ishihara, 2024	1402	[1103; 1782]	1849	[1499; 2281]
	Senders, 2021	234,1	[158; 346,7]	268,2	[157; 458,3]
	**Overall**	**266,2**	**[33,67; 2104,89]**	**317,62**	**[36,41; 2770,9]**
	**Schedule 3+1**	**440,56**	**[35,63; 5448,06]**	**534,56**	**[35,34; 8084,76]**
	**Schedule 2+1**	**59**	**[42; 84]**	**68**	**[48; 96]**
**Additional**					
8	Korbal, 2024	164	[133; 203]	17	[15; 18]
	Senders, 2024	665	[503; 880]	18	[17; 20]
	Ishihara, 2024	1541	[1220; 1946]	16	[15; 18]
	Senders, 2021	475,5	[346,6; 652,2]	17,3	[15,2; 19,8]
	**Overall**	**531,21**	**[120,99; 2332,35]**	**17,07**	**[15,72; 18,54]**
	**Schedule 3+1**	**792,02**	**[175,30; 3578,39]**	**17,09**	**[14,59; 20,02]**
	**Schedule 2+1**	**164**	**[133; 203]**	**17**	**[15; 18]**
10A	Korbal, 2024	855	[610; 1199]	39	[34; 44]
	Senders, 2024	2558	[1869; 3501]	37	[33; 42]
	Ishihara, 2024	6780	[5436; 8456]	40	[33; 47]
	Senders, 2021	1846,7	[1347,6; 2530,5]	36,8	[31,6; 42,8]
	**Overall**	**2301,83**	**[586,28; 9037,33]**	**38**	**[35,78; 40,36]**
	**Schedule 3+1**	**3199,13**	**[591,45; 17303,97]**	**37,60**	**[33,94; 41,46]**
	**Schedule 2+1**	**855**	**[610; 1199]**	**39**	**[34; 44]**
11A	Korbal, 2024	327	[253; 423]	49	[47; 51]
	Senders, 2024	289	[212; 395]	50	[46; 55]
	Ishihara, 2024	1838	[1451; 2327]	58	[47; 71]
	Senders, 2021	423,9	[287; 626,3]	19,9	[14,6; 27,2]
	**Overall**	**523,18**	**[133,21; 2054,86]**	**41,62**	**[19,51; 88,78]**
	**Schedule 3+1**	**612,03**	**[53,63; 6984,74]**	**39,11**	**[9,41; 162,49]**
	**Schedule 2+1**	**327**	**[253; 423]**	**49**	**[47; 51]**
12F	Korbal, 2024	4788	[2779; 6067]	26	[23; 28]
	Senders, 2024	7677	[5952; 9901]	28	[24; 33]
	Ishihara, 2024	21475	[14378; 32074]	24	[24; 25]
	Senders, 2021	6084,9	[4578,8; 8086,4]	26,5	[22,7; 30,9]
	**Overall**	**8211,29**	**[2907,57; 23189,53]**	**25,32**	**[22,83; 28,09]**
	**Schedule 3+1**	**9882,05**	**[1893,77; 51566,26]**	**25,34**	**[20,79; 30,88]**
	**Schedule 2+1**	**4788**	**[2779; 6067]**	**26**	**[23; 28]**
15B	Korbal, 2024	846	[605; 1183]	17	[15; 19]
	Senders, 2024	1560	[1090; 2233]	18	[16; 22]
	Ishihara, 2024	5707	[4129; 7889]	17	[15; 20]
	Senders, 2021	1085,8	[702,9; 1677,4]	22,8	[17,4; 229,9]
	**Overall**	**1697,89**	**[437,73; 6585,9]**	**18,01**	**[14,97; 21,67]**
	**Schedule 3+1**	**2146,10**	**[244,86; 18810,07]**	**18,57**	**[13,11; 26,29]**

	**Schedule 2+1**	**846**	**[605; 1183]**	**17**	**[15; 19]**
22F	Korbal, 2024	4444	[3666; 5386]	10	[9; 11]
	Senders, 2024	6797	[5170; 8936]	9	[9; 9]
	Ishihara, 2024	19276	[14969; 24822]	10	[8; 11]
	Senders, 2021	6304	[4430,3; 8970,1]	11,1	[8,6; 14,1]
	**Overall**	**7781,5**	**[2830,94; 21389,26]**	**10,13**	**[9,14; 11,22]**
	**Schedule 3+1**	**9440,67**	**[1989,83; 44790,97]**	**9,57**	**[7,54; 12,13]**
	**Schedule 2+1**	**4444**	**[3666; 5386]**	**10**	**[9; 11]**
33F	Korbal, 2024	2373	[1759; 3202]	178	[163; 195]
	Senders, 2024	7388	[4803; 11365]	198	[177; 220]
	Ishihara, 2024	15931	[11550; 21974]	177	[160; 195]
	Senders, 2021	7266,5	[4855,4; 10875,1]	60,7	[39,5; 93,3]
	**Overall**	**6692,72**	**[1902,78; 23540,57]**	**143,88**	**[61,42; 337,07]**
	**Schedule 3+1**	**9647,12**	**[3114,48; 29881,98]**	**132,17**	**[26,96; 647,96]**
	**Schedule 2+1**	**2373**	**[1759; 3202]**	**178**	**[163; 195]**

GMT of OPA: geometric mean titers of opsonophagocitic activitiy; CI: confidence interval.
Overall: includes the four studies.
Schedule 3+1: includes the three studies with Schedule 3+1: Senders, 2024; Ishihara, 2024; and Shenders, 2021.

Table S6. Summary of meta-analysis results of OPA after booster dose in PCV20 and PCV13 for shared and additional serotypes.

Serotype	Author	Booster dose			
		PCV20		PCV13	
		GMT of OPA	CI 95%	GMT of OPA	CI 95%
**Common**					
1	Korbal, 2024	54	[43; 69]	101	[79; 129]
	Senders, 2024	36	[27; 48]	66	[50; 87]
	Ishihara, 2024	194	[150; 251]	386	[306; 486]
	Senders, 2021	50,4	[35,4; 71,9]	92,9	[65,9; 131,1]
	**Overall**	**66,22**	**[20,34; 215,57]**	**124,81**	**[36,09; 431,69]**
	**Schedule 3+1**	**70,85**	**[7,69; 652,41]**	**133,79**	**[12,98; 1379,50]**
	**Schedule 2+1**	**54**	**[43; 69]**	**101**	**[79; 129]**
3	Korbal, 2024	99	[84; 117]	129	[34; 47]
	Senders, 2024	62	[49; 78]	102	[53; 76]
	Ishihara, 2024	150	[126; 179]	193	[147; 196]
	Senders, 2021	93	[73, 118,4]	109,3	[50,1; 76,2]
	**Overall**	**96,74**	**[54,41; 171,99]**	**129,21**	**[82,00; 203,58]**
	**Schedule 3+1**	**95,74**	**[31,78; 288,44]**	**129,22**	**[54,12; 308,53]**
	**Schedule 2+1**	**99**	**[84; 117]**	**129**	**[34; 47]**
4	Korbal, 2024	904	[752; 1086]	992	[777; 1266]
	Senders, 2024	621	[435; 887]	961	[714; 1294]
	Ishihara, 2024	1544	[1224; 1948]	2038	[1471; 2825]
	Senders, 2021	490,3	[310,6; 774]	662,5	[415,3; 1056,9]
	**Overall**	**828,15**	**[374,97; 1829,06]**	**1079,03**	**[517,31; 2250,67]**
	**Schedule 3+1**	**794,11**	**[174,06; 3622,85]**	**1106,15**	**[268,72; 4553,36]**
	**Schedule 2+1**	**904**	**[752; 1086]**	**992**	**[777; 1266]**
5	Korbal, 2024	60	[50; 72]	82	[66; 101]
	Senders, 2024	55	[45; 67]	69	[54; 87]
	Ishihara, 2024	141	[112; 179]	248	[204; 303]
	Senders, 2021	78,7	[59,3; 104,5]	112,8	[85,6; 148,6]
	**Overall**	**77,47**	**[39,35; 152,51]**	**112,40**	**[45,44; 278,03]**
	**Schedule 3+1**	**84,66**	**[25,84; 277,37]**	**124,88**	**[25,00; 623,72]**
	**Schedule 2+1**	**60**	**[50; 72]**	**82**	**[66; 101]**
6A	Korbal, 2024	1101	[897; 1350]	1304	[1018; 1671]
	Senders, 2024	1384	[1092; 1753]	1767	[1329; 2348]
	Ishihara, 2024	3489	[2831; 4300]	5455	[4379; 6795]
	Senders, 2021	1671,4	[1181,4; 2364,5]	2155,8	[1716,2; 2708,1]
	**Overall**	**1728,47**	**[774,58; 3857,06]**	**2287,81**	**[855,41; 6118,78]**
	**Schedule 3+1**	**2019,44**	**[592,08; 6887,77]**	**2759,50**	**[618,03; 12321,07]**
	**Schedule 2+1**	**1101**	**[897; 1350]**	**1304**	**[1018; 1671]**

6B	Korbal, 2024	537	[408; 706]	864	[664; 1125]
	Senders, 2024	666	[489; 906]	1211	[861; 1703]
	Ishihara, 2024	2552	[2006; 3247]	4319	[3478; 5362]
	Senders, 2021	1354,9	[987,1; 1859,8]	1808,1	[1279,4; 2575,4]
	**Overall**	**1057,04**	**[340,56; 3280,8]**	**1698,38**	**[557,69; 5172,21]**
	**Schedule 3+1**	**1326,87**	**[249,06; 7069,04]**	**2137,45**	**[420,71; 10859,47]**
	**Schedule 2+1**	**537**	**[408; 706]**	**864**	**[664; 1125]**
7F	Korbal, 2024	1811	[1553; 2112]	2197	[1905; 2533]
	Senders, 2024	2022	[1673; 2444]	2099	[1741; 2531]
	Ishihara, 2024	4703	[3704; 5972]	6361	[5024; 8054]
	Senders, 2021	2590,7	[2143,2; 3131,7]	3280,7	[2576,5; 4177,5]
	**Overall**	**2568,52**	**[1309,23; 5039,07]**	**3115,62**	**[1379,02; 7039,1]**
	**Schedule 3+1**	**2895,88**	**[990.12; 8469,80]**	**3514,52**	**[877,62; 14074,24]**
	**Schedule 2+1**	**1811**	**[1553; 2112]**	**2197**	**[1905; 2533]**
9V	Korbal, 2024	3254	[2596; 4079]	4544	[3681; 5610]
	Senders, 2024	2609	[1913; 3558]	3210	[2500; 4123]
	Ishihara, 2024	4201	[3418; 5164]	5162	[4349; 6127]
	Senders, 2021	1280,2	[981,6; 1669,7]	2030	[1469,6; 2804,3]
	**Overall**	**2611,25**	**[1156,76; 5894,6]**	**3570,38**	**[1851,41; 6885,35]**
	**Schedule 3+1**	**2420,14**	**[544,60; 10754,82]**	**3274,50**	**[1028,40; 10426,26]**
	**Schedule 2+1**	**3254**	**[2596; 4079]**	**4544**	**[3681; 5610]**
14	Korbal, 2024	738	[606; 899]	926	[751; 1142]
	Senders, 2024	667	[523; 850]	593	[462; 761]
	Ishihara, 2024	2005	[1631; 2463]	1706	[1385; 2102]
	Senders, 2021	933,8	[715,8; 1218,1]	1127,9	[831,5; 1530]
	**Overall**	**981,53**	**[443,23; 2173,59]**	**1015,64**	**[503,57; 2048,4]**
	**Schedule 3+1**	**1080,44**	**[264,94; 4406,15]**	**1047,27**	**[277,05; 3958,70]**
	**Schedule 2+1**	**738**	**[606; 899]**	**926**	**[751; 1142]**
18C	Korbal, 2024	1296	[1048; 1602]	1870	[1489; 2348]
	Senders, 2024	1973	[1472; 3643]	2425	[1914; 3072]
	Ishihara, 2024	4249	[3355; 5381]	6315	[5081; 7848]
	Senders, 2021	2016,2	[1596,5; 2546,1]	2703,3	[1980,4; 3690]
	**Overall**	**2167,13**	**[974,25; 4820,62]**	**2972,14**	**[1278,3; 6910,46]**
	**Schedule 3+1**	**2609,03**	**[869,11; 7832,22]**	**3476,24**	**[938,84; 12871,47]**
	**Schedule 2+1**	**1296**	**[1048; 1602]**	**1870**	**[1489; 2348]**
19A	Korbal, 2024	754	[627; 907]	707	[558; 896]
	Senders, 2024	844	[622; 1145]	1357	[1007; 1829]
	Ishihara, 2024	1722	[1379; 2151]	2534	[2044; 3143]
	Senders, 2021	651,3	[519,9; 816]	874,8	[650,6; 1176,4]
	**Overall**	**919,43**	**[460,95; 1833,95]**	**1211,03**	**[491,04; 2986,75]**
	**Schedule 3+1**	**984,28**	**[279,55; 3465,52]**	**1454,13**	**[383,26; 5517,08]**
	**Schedule 2+1**	**754**	**[627; 907]**	**707**	**[558; 896]**
19F	Korbal, 2024	183	[140; 237]	258	[192; 347]
	Senders, 2024	246	[179; 337]	373	[272; 513]
	Ishihara, 2024	962	[753; 1230]	1783	[1364; 2331]

	Senders, 2021	500,5	[337,2; 743]	751	[546,6; 1032]
	**Overall**	**383,69**	**[116,63; 1262,22]**	**600,25**	**[154,5; 2332,1]**
	**Schedule 3+1**	**493,55**	**[89,64; 2717,38]**	**796,08**	**[113,35; 5591,07]**
	**Schedule 2+1**	**183**	**[140; 237]**	**258**	**[192; 347]**
23F	Korbal, 2024	697	[530; 917]	975	[734; 1296]
	Senders, 2024	827	[554; 1235]	1532	[1118; 2100]
	Ishihara, 2024	2052	[1668; 2523]	3772	[2966; 4796]
	Senders, 2021	693,1	[519,9; 923,8]	1253,9	[894,6; 1757,6]
	**Overall**	**960,63**	**[415,01; 2223,55]**	**1639,39**	**[637,6; 4215,22]**
	**Schedule 3+1**	**1068,04**	**[247,11; 4616,13]**	**1952,17**	**[451,13; 8447,64]**
	**Schedule 2+1**	**697**	**[530; 917]**	**975**	**[734; 1296]**
**Additional**					
8	Korbal, 2024	1398	[1088; 1796]	31	[25; 39]
	Senders, 2024	1228	[901; 1673]	26	[31; 31]
	Ishihara, 2024	3208	[2525; 4077]	27	[20; 36]
	Senders, 2021	1721,7	[1298,7; 2282,6]	35,2	[23,2; 53,3]
	**Overall**	**1764,53**	**[893,39; 3485,1]**	**28,67**	**[23,63; 34,79]**
	**Schedule 3+1**	**1906,25**	**[566,71; 6412,09]**	**27,79**	**[19,74; 39,14]**
	**Schedule 2+1**	**1398**	**[1088; 1796]**	**31**	**[25; 39]**
10A	Korbal, 2024	3403	[2600; 4455]	69	[52; 91]
	Senders, 2024	3674	[2746; 4916]	57	[44; 74]
	Ishihara, 2024	8269	[6252; 10937]	87	[56; 136]
	Senders, 2021	2697,7	[2082,3; 3494,8]	63,1	[43,2; 92,3
	**Overall**	**4080,91**	**[1877,4; 8870,67]**	**65,97**	**[50,77; 85,7]**
	**Schedule 3+1**	**4338,83**	**[1029,78; 18281,06]**	**65,21**	**[38,87; 109,39]**
	**Schedule 2+1**	**3403**	**[2600; 4455]**	**69**	**[52; 91]**
11A	Korbal, 2024	2966	[2212; 3978]	66	[51; 85]
	Senders, 2024	2728	[1975; 3768]	69	[53; 89]
	Ishihara, 2024	4200	[3187; 5534]	90	[62; 132]
	Senders, 2021	5307,7	[4007,3; 7030,2]	76,7	[33,6; 175,2]
	**Overall**	**3687,87**	**[2255,19; 6030,69]**	**71,96**	**[57,98; 89,29]**
	**Schedule 3+1**	**3961,55**	**[1725,37; 9095,93]**	**75,79**	**[52,31; 109,81]**
	**Schedule 2+1**	**2966**	**[2212; 3978]**	**66**	**[51; 85]**
12F	Korbal, 2024	5501	[4499; 6725]	29	[25; 35]
	Senders, 2024	9320	[7037; 12343]	31	[26; 37]
	Ishihara, 2024	18899	[14215; 25125]	43	[31; 60]
	Senders, 2021	8518,9	[6030,5; 12034,1]	27,8	[22,6; 34,1]
	**Overall**	**9486,26**	**[4182,79; 21514,14]**	**31,25**	**[23,73; 41,14]**
	**Schedule 3+1**	**11505,53**	**[3881,49; 34104,76]**	**32,41**	**[19,20; 54,71]**
	**Schedule 2+1**	**5501**	**[4499; 6725]**	**29**	**[25; 35]**
15B	Korbal, 2024	2676	[1948; 3677]	23	[18; 30]
	Senders, 2024	3035	[2138; 4308]	23	[17; 30]
	Ishihara, 2024	7770	[6448; 9363]	32	[20; 50]
	Senders, 2021	3087,9	[2304,5; 4137,6]	28,2	[19,1; 41,7]
	**Overall**	**3787,35**	**[1706,91; 8403,51]**	**25,09**	**[19,85; 31,72]**
	**Schedule 3+1**	**4231,50**	**[1097,76; 16311,12]**	**26,34**	**[17,36; 39,95]**

	**Schedule 2+1**	**2676**	**[1948; 3677]**	**23**	**[18; 30]**
22F	Korbal, 2024	6523	[4848; 8777]	17	[13; 24]
	Senders, 2024	11077	[7956; 15422]	15	[11; 20]
	Ishihara, 2024	23480	[17229; 21998]	18	[12; 27]
	Senders, 2021	9339,2	[6575,3; 13265]	16,5	[11; 24,9]
	**Overall**	**11376,09**	**[4749,45; 27248,52]**	**16,4**	**[14,48; 18,57]**
	**Schedule 3+1**	**13731,68**	**[3984,78; 47319,76]**	**16,14**	**[12,79; 20,35]**
	**Schedule 2+1**	**6523**	**[4848; 8777]**	**17**	**[13; 24]**
33F	Korbal, 2024	11315	[8107; 15794]	708	[545; 920]
	Senders, 2024	19216	[13193; 27990]	363	[292; 451]
	Ishihara, 2024	29963	[18722; 38830]	658	[480; 904]
	Senders, 2021	8244,6	[5797,7; 11724,2]	135,6	[77,7; 236,5]
	**Overall**	**15180,25**	**[6124,22; 37627,67]**	**399,53**	**[121,78; 1310,71]**
	**Schedule 3+1**	**16779,89**	**[3284,46; 85726,20]**	**327,09**	**[46,47; 1310,71]**
	**Schedule 2+1**	**11315**	**[8107; 15794]**	**708**	**[545; 920]**

GMT of OPA: geometric mean titers of opsonophagocitic activitiy; CI: confidence interval.

Overall: includes the four studies.

Schedule 3+1: includes the three studies with Schedule 3+1: Senders, 2024; Ishihara, 2024; and Shenders, 2021.

Table S7. Quality Grading of Evidence

	Certainty Assessment					Patients, n				
**Outcome [e]**	**Risk of Bias**	**Inconsistency**	**Indirectness**	**Imprecision**	**Other Considerations**	**PCV20**	**PCV13**	**GMR (95%-CI)**	**Certainty**	**Importance**
**GMR after primary series**										
Serotype 1	not serious	not serious [b]	not serious	not serious	publication bias strongly suspected [b]	2051	2042	**0.66** (0.55 to 0.78)	⊕⊕⊕◯ Moderate [b]	Important
Serotype 3	not serious	not serious	not serious	not serious	none	2051	2042	**0.71** (0.68 to 0.75)	⊕⊕⊕⊕ High	Important
Serotype 4	not serious	serious [c]	not serious	not serious	publication bias strongly suspected [c]	2051	2042	**0.67** (0.53 to 0.84)	⊕⊕◯◯ Low [c]	Important
Serotype 5	not serious	not serious [b]	not serious	not serious	publication bias strongly suspected [b]	2051	2042	**0.66** (0.53 to 0.83)	⊕⊕⊕◯ Moderate [b]	Important
Serotype 6A	not serious	serious [c]	not serious	not serious	publication bias strongly suspected [c]	2051	2042	**0.67** (0.48 to 0.94)	⊕⊕◯◯ Low [c]	Important
Serotype 6B	not serious	not serious	not serious	not serious	none	2051	2042	**0.55** (0.47 to 0.65)	⊕⊕⊕⊕ High	Important
Serotype 7F	not serious	not serious	not serious	not serious	none	2051	2042	**0.75** (0.69 to 0.82)	⊕⊕⊕⊕ High	Important
Serotype 9V	not serious	not serious [b]	not serious	not serious	publication bias strongly suspected [b]	2051	2042	**0.7** (0.56 to 0.87)	⊕⊕⊕◯ Moderate [b]	Important
Serotype 14	not serious	not serious	not serious	not serious	none	2051	2042	**0.82** (0.77 to 0.87)	⊕⊕⊕⊕ High	Important
Serotype 18C	not serious	not serious [a]	not serious	not serious	publication bias strongly suspected [a]	2051	2042	**0.75** (0.66 to 0.85)	⊕⊕⊕⊕ High [a]	Important
Serotype 19A	not serious	serious [c]	not serious	not serious	publication bias strongly suspected [c]	2051	2042	**0.74** (0.58 to 0.94)	⊕⊕◯◯ Low [c]	Important
Serotype 19F	not serious	not serious [a]	not serious	not serious	publication bias strongly suspected [a]	2051	2042	**0.77** (0.69 to 0.86)	⊕⊕⊕⊕ High [a]	Important
Serotype 23F	not serious	not serious [b]	not serious	not serious	publication bias strongly suspected [b]	2051	2042	**0.63** (0.5 to 0.79)	⊕⊕⊕◯ Moderate [b]	Important
Serotype 8	not serious	very serious [d]	not serious	not serious	publication bias strongly suspected [d]	2051	2042	**4.6** (0.67 to 31.69)	⊕◯◯◯ Very low [d]	Important

Serotype 10A	not serious	very serious [d]	not serious	not serious	publication bias strongly suspected [d]	2051	2042	**1.37** (0.51 to 3.72)	⊕◯◯◯ Very low [d]	Important
Serotype 11A	not serious	very serious [d]	not serious	not serious	publication bias strongly suspected [d]	2051	2042	**4.82** (0.6 to 39.07)	⊕◯◯◯ Very low [d]	Important
Serotype 12F	not serious	very serious [d]	not serious	not serious	publication bias strongly suspected [d]	2051	2042	**1.05** (0.4 to 2.77)	⊕◯◯◯ Very low [d]	Important
Serotype 15B	not serious	very serious [d]	not serious	not serious	publication bias strongly suspected [d]	2051	2042	**10.64** (1.81 to 62.59)	⊕◯◯◯ Very low [d]	Important
Serotype 22F	not serious	very serious [d]	not serious	not serious	publication bias strongly suspected [d]	2051	2042	**8.04** (1.57 to 41.32)	⊕◯◯◯ Very low [d]	Important
Serotype 33F	not serious	very serious [d]	not serious	not serious	publication bias strongly suspected [d]	2051	2042	**2.48** (1.13 to 5.44)	⊕◯◯◯ Very low [d]	Important

[a] Heterogeneity not important
[b] Moderate heterogeneity
[c] High heterogeneity
[d] Very high heterogeneity
[e] All studies were randomized controlled trials

	**Certainty Assessment**					**Patients, n**				
**Outcome [e]**	**Risk of Bias**	**Inconsistency**	**Indirectness**	**Imprecision**	**Other Considerations**	**PCV20**	**PCV13**	**DP (95%-CI)**	**Certainty**	**Importance**
**DP after primary series**										
Serotype 1	not serious	serious [c]	not serious	not serious	publication bias strongly suspected [c]	2051	2042	**-5.99** (-15.96 to 3.97)	⊕⊕◯◯ Low [c]	Important
Serotype 3	not serious	very serious [d]	not serious	not serious	publication bias strongly suspected [d]	2051	2042	**-11.46** (-22,66; -0,26)	⊕◯◯◯ Very low [d]	Important
Serotype 4	not serious	serious [c]	not serious	not serious	publication bias strongly suspected [c]	2051	2042	**-6.27** (-12,77; 0,22)	⊕⊕◯◯ Low [c]	Important
Serotype 5	not serious	serious [c]	not serious	not serious	publication bias strongly suspected [c]	2051	2042	**-6.01** (-13,05; 1,02)	⊕⊕◯◯ Low [c]	Important
Serotype 6A	not serious	serious [c]	not serious	not serious	publication bias strongly suspected [c]	2051	2042	**-5.75** (-16,21; 4,71)	⊕⊕◯◯ Low [c]	Important
Serotype 6B	not serious	serious [c]	not serious	not serious	publication bias strongly suspected [c]	2051	2042	**-8** (-16,94; 0,94)	⊕⊕◯◯ Low [c]	Important
Serotype 7F	not serious	not serious [a]	not serious	not serious	publication bias strongly suspected [a]	2051	2042	**-1.29** (-4,09; 1,50)	⊕⊕⊕⊕ High [a]	Important

Serotype 9V	not serious	serious [c]	not serious	not serious	publication bias strongly suspected [c]	2051	2042	**-6.27** (-16,08; 3,53)	⊕⊕◯◯ Low [c]	Important
Serotype 14	not serious	not serious	not serious	not serious	none	2051	2042	**-1.26** (-2,82; 0,30)	⊕⊕⊕⊕ High	Important
Serotype 18C	not serious	not serious [a]	not serious	not serious	publication bias strongly suspected [a]	2051	2042	**-2.17** (-5,18; 0,83)	⊕⊕⊕⊕ High [a]	Important
Serotype 19A	not serious	not serious	not serious	not serious	none	2051	2042	**-0.63** (-1,93; 0,68)	⊕⊕⊕⊕ High	Important
Serotype 19F	not serious	not serious	not serious	not serious	none	2051	2042	**-0.02** (-1,54; 1,50)	⊕⊕⊕⊕ High	Important
Serotype 23F	not serious	serious [c]	not serious	not serious	publication bias strongly suspected [c]	2051	2042	**-8.21** (-19.70; 3.28)	⊕⊕◯◯ Low [c]	Important
Serotype 8	not serious	very serious [d]	not serious	not serious	publication bias strongly suspected [d]	2051	2042	**23.66** (-15,54; 62,86)	⊕◯◯◯ Very low [d]	Important
Serotype 10A	not serious	very serious [d]	not serious	not serious	publication bias strongly suspected [d]	2051	2042	**-9.55** (-36,29; 17,19)	⊕◯◯◯ Very low [d]	Important
Serotype 11A	not serious	very serious [d]	not serious	not serious	publication bias strongly suspected [d]	2051	2042	**21.66** (-17,05; 60,36)	⊕◯◯◯ Very low [d]	Important
Serotype 12F	not serious	serious [c]	not serious	not serious	publication bias strongly suspected [c]	2051	2042	**-10.93** (-25,94; 4,08)	⊕⊕◯◯ Low [c]	Important
Serotype 15B	not serious	very serious [d]	not serious	not serious	publication bias strongly suspected [d]	2051	2042	**23.26** (-14,16; 60,68)	⊕◯◯◯ Very low [d]	Important
Serotype 22F	not serious	very serious [d]	not serious	not serious	publication bias strongly suspected [d]	2051	2042	**23.50** (-13,53; 60,54)	⊕◯◯◯ Very low [d]	Important
Serotype 33F	not serious	serious [c]	not serious	not serious	publication bias strongly suspected [c]	2051	2042	**5.21** (-3,10; 13,51)	⊕⊕◯◯ Low [c]	Important

[a] Heterogeneity not important
[b] Moderate heterogeneity
[c] High heterogeneity
[d] Very high heterogeneity
[e] All studies were randomized controlled trials

	**Certainty Assessment**					**Patients, n**				
**Outcome [e]**	**Risk of Bias**	**Inconsistency**	**Indirectness**	**Imprecision**	**Other Considerations**	**PCV20**	**PCV13**	**GMR (95%-CI)**	**Certainty**	**Importance**
**GMR after booster dose**										
Serotype 1	not serious	not serious	not serious	not serious	none	2051	2042	**0.67** (0,60; 0,75)	⊕⊕⊕⊕ High	Important

Serotype 3	not serious	not serious	not serious	not serious	none	2051	2042	**0.67** (0,62; 0,73)	⊕⊕⊕⊕ High	Important
Serotype 4	not serious	not serious	not serious	not serious	none	2051	2042	**0.76** (0,70; 0,82)	⊕⊕⊕⊕ High	Important
Serotype 5	not serious	not serious	not serious	not serious	none	2051	2042	**0.71** (0,64; 0,78)	⊕⊕⊕⊕ High	Important
Serotype 6A	not serious	not serious [a]	not serious	not serious	publication bias strongly suspected [a]	2051	2042	**0.72** (0,64; 0,85)	⊕⊕⊕⊕ High [a]	Important
Serotype 6B	not serious	not serious [a]	not serious	not serious	publication bias strongly suspected [a]	2051	2042	**0.66** (0,56; 0,76)	⊕⊕⊕⊕ High [a]	Important
Serotype 7F	not serious	not serious [a]	not serious	not serious	publication bias strongly suspected [a]	2051	2042	**0.72** (0,65; 0,80)	⊕⊕⊕⊕ High [a]	Important
Serotype 9V	not serious	not serious [a]	not serious	not serious	publication bias strongly suspected [a]	2051	2042	**0.74** (0,67; 0,83)	⊕⊕⊕⊕ High [a]	Important
Serotype 14	not serious	not serious	not serious	not serious	none	2051	2042	**0.84** (0,75; 0,94)	⊕⊕⊕⊕ High	Important
Serotype 18C	not serious	not serious	not serious	not serious	none	2051	2042	**0.72** (0,65; 0,79)	⊕⊕⊕⊕ High	Important
Serotype 19A	not serious	not serious	not serious	not serious	none	2051	2042	**0.84** (0,81; 0,87)	⊕⊕⊕⊕ High	Important
Serotype 19F	not serious	not serious	not serious	not serious	none	2051	2042	**0.82** (0,75; 0,89)	⊕⊕⊕⊕ High	Important
Serotype 23F	not serious	not serious	not serious	not serious	none	2051	2042	**0.62** (0,58; 0,65)	⊕⊕⊕⊕ High	Important
Serotype 8	not serious	very serious [d]	not serious	not serious	publication bias strongly suspected [d]	2051	2042	**1.32** (0,79; 2,22)	⊕◯◯◯ Very low [d]	Important
Serotype 10A	not serious	very serious [d]	not serious	not serious	publication bias strongly suspected [d]	2051	2042	**2.23** (1,37; 3,63)	⊕◯◯◯ Very low [d]	Important
Serotype 11A	not serious	serious [c]	not serious	not serious	publication bias strongly suspected [c]	2051	2042	**1.51** (1,23; 1,86)	⊕⊕◯◯ Low [c]	Important
Serotype 12F	not serious	very serious [d]	not serious	not serious	publication bias strongly suspected [d]	2051	2042	**0.68** (0,47; 0,99)	⊕◯◯◯ Very low [d]	Important
Serotype 15B	not serious	serious [c]	not serious	not serious	publication bias strongly suspected [c]	2051	2042	**5.09** (3,87; 6,69)	⊕⊕◯◯ Low [c]	Important
Serotype 22F	not serious	very serious [d]	not serious	not serious	publication bias strongly suspected [d]	2051	2042	**3.94** (2,83; 5,49)	⊕◯◯◯ Very low [d]	Important
Serotype 33F	not serious	very serious [d]	not serious	not serious	publication bias strongly suspected [d]	2051	2042	**2.41** (1,08; 5,38)	⊕◯◯◯ Very low [d]	Important

[a] *Heterogeneity not important*
[b] *Moderate heterogeneity*

[c] *High heterogeneity*
[d] *Very high heterogeneity*
[e] *All studies were randomized controlled trials*

	**Certainty Assessment**					**Patients, n**				
**Outcome [e]**	**Risk of Bias**	**Inconsistency**	**Indirectness**	**Imprecision**	**Other Considerations**	**PCV20**	**PCV13**	**DP (95%-CI)**	**Certainty**	**Importance**
**DP after booster dose**										
Serotype 1	not serious	not serious [a]	not serious	not serious	publication bias strongly suspected [a]	1602	1590	**-1.92** (-3,1; 0,9)	⊕⊕⊕⊕ High [a]	Important
Serotype 3	not serious	not serious	not serious	not serious	none	1602	1590	**-11.34** (-20,87; -1,81)	⊕⊕⊕⊕ High	Important
Serotype 4	not serious	not serious	not serious	not serious	none	1602	1590	**-0.06** (-0,69; 0,57)	⊕⊕⊕⊕ High	Important
Serotype 5	not serious	not serious	not serious	not serious	none	1602	1590	**0.29** (-0,97; 1,54)	⊕⊕⊕⊕ High	Important
Serotype 6A	not serious	not serious	not serious	not serious	none	1602	1590	**-0.24** (-1,79; 1,32)	⊕⊕⊕⊕ High	Important
Serotype 6B	not serious	not serious [a]	not serious	not serious	publication bias strongly suspected [a]	1602	1590	**-0.03** (-7,05; 6,98)	⊕⊕⊕⊕ High [a]	Important
Serotype 7F	not serious	not serious	not serious	not serious	none	1602	1590	**-0.39** (-0,46; -033)	⊕⊕⊕⊕ High	Important
Serotype 9V	not serious	not serious	not serious	not serious	none	1602	1590	**-0.06** (-5,08; 4,95)	⊕⊕⊕⊕ High	Important
Serotype 14	not serious	not serious	not serious	not serious	none	1602	1590	**-0.73** (-6,04; 4,59)	⊕⊕⊕⊕ High	Important
Serotype 18C	not serious	not serious	not serious	not serious	none	1602	1590	**0.56** (-3,74; 4,86)	⊕⊕⊕⊕ High	Important
Serotype 19A	not serious	not serious	not serious	not serious	none	1602	1590	**0.07** (-0,50; 0,65)	⊕⊕⊕⊕ High	Important
Serotype 19F	not serious	not serious	not serious	not serious	none	1602	1590	**0.06** (-1,85; 1,96)	⊕⊕⊕⊕ High	Important
Serotype 23F	not serious	not serious	not serious	not serious	none	1602	1590	**-0.87** (-1,46; -0,28)	⊕⊕⊕⊕ High	Important
Serotype 8	not serious	not serious	not serious	not serious	none	1602	1590	**2.19** (0,34; 4,03)	⊕⊕⊕⊕ High	Important
Serotype 10A	not serious	not serious	not serious	not serious	none	1602	1590	**0.6** (0,53; 0,66)	⊕⊕⊕⊕ High	Important

Serotype 11A	not serious	not serious	not serious	not serious	none	1602	1590	**1.45** (-1,01; 3,91)	⊕⊕⊕⊕ High	Important
Serotype 12F	not serious	not serious	not serious	not serious	none	1602	1590	**-1.5** (-9,13; 6,14)	⊕⊕⊕⊕ High	Important
Serotype 15B	not serious	not serious	not serious	not serious	none	1602	1590	**2.45** (-0,002; 4,91)	⊕⊕⊕⊕ High	Important
Serotype 22F	not serious	not serious	not serious	not serious	none	1602	1590	**2.26** (-0,18; 4,69)	⊕⊕⊕⊕ High	Important
Serotype 33F	not serious	not serious	not serious	not serious	none	1602	1590	**1.98** (-3,49; 7,45)	⊕⊕⊕⊕ High	Important

[a] Heterogeneity not important
[b] Moderate heterogeneity
[c] High heterogeneity
[d] Very high heterogeneity
[e] All studies were randomized controlled trials

	Certainty Assessment					Patients, n				
**Outcome [e]**	**Risk of Bias**	**Inconsistency**	**Indirectness**	**Imprecision**	**Other Considerations**	**PCV20**	**PCV13**	**OPA (95%-CI)**	**Certainty**	**Importance**
**OPA PCV20 after primary series**										
Serotype 1	not serious	very serious [d]	not serious	not serious	publication bias strongly suspected [d]	2051	2042	**23.96** (8,89; 64,61)	⊕◯◯◯ Very low [d]	Important
Serotype 3	not serious	very serious [d]	not serious	not serious	publication bias strongly suspected [d]	2051	2042	**55.63** (22,62; 136,84)	⊕◯◯◯ Very low [d]	Important
Serotype 4	not serious	very serious [d]	not serious	not serious	publication bias strongly suspected [d]	2051	2042	**529.61** (158,37; 1771,09)	⊕◯◯◯ Very low [d]	Important
Serotype 5	not serious	very serious [d]	not serious	not serious	publication bias strongly suspected [d]	2051	2042	**37.5** (13,85; 101,54)	⊕◯◯◯ Very low [d]	Important
Serotype 6A	not serious	very serious [d]	not serious	not serious	publication bias strongly suspected [d]	2051	2042	**949.33** (224,62; 4012,18)	⊕◯◯◯ Very low [d]	Important
Serotype 6B	not serious	very serious [d]	not serious	not serious	publication bias strongly suspected [d]	2051	2042	**328.06** (35,97; 2992,36)	⊕◯◯◯ Very low [d]	Important
Serotype 7F	not serious	very serious [d]	not serious	not serious	publication bias strongly suspected [d]	2051	2042	**1622.59** (520; 5063,03)	⊕◯◯◯ Very low [d]	Important
Serotype 9V	not serious	very serious [d]	not serious	not serious	publication bias strongly suspected [d]	2051	2042	**642.97** (148,08; 2791,87)	⊕◯◯◯ Very low [d]	Important
Serotype 14	not serious	very serious [d]	not serious	not serious	publication bias strongly suspected [d]	2051	2042	**622.82** (156,77; 2474,29)	⊕◯◯◯ Very low [d]	Important

Serotype 18C	not serious	very serious [d]	not serious	not serious	publication bias strongly suspected [d]	2051	2042	**1421.46** (343,57; 5881,01)	⊕◯◯◯ Very low [d]	Important
Serotype 19A	not serious	very serious [d]	not serious	not serious	publication bias strongly suspected [d]	2051	2042	**138.15** (29,17; 654,17)	⊕◯◯◯ Very low [d]	Important
Serotype 19F	not serious	very serious [d]	not serious	not serious	publication bias strongly suspected [d]	2051	2042	**138.39** (35,96; 532,64)	⊕◯◯◯ Very low [d]	Important
Serotype 23F	not serious	very serious [d]	not serious	not serious	publication bias strongly suspected [d]	2051	2042	**266.2** (33,67; 2104,89)	⊕◯◯◯ Very low [d]	Important
Serotype 8	not serious	very serious [d]	not serious	not serious	publication bias strongly suspected [d]	2051	2042	**531.21** (120,99; 2332,35)	⊕◯◯◯ Very low [d]	Important
Serotype 10A	not serious	very serious [d]	not serious	not serious	publication bias strongly suspected [d]	2051	2042	**2301.83** (586,28; 9037,33)	⊕◯◯◯ Very low [d]	Important
Serotype 11A	not serious	very serious [d]	not serious	not serious	publication bias strongly suspected [d]	2051	2042	**523.18** (133,21; 2054,86)	⊕◯◯◯ Very low [d]	Important
Serotype 12F	not serious	very serious [d]	not serious	not serious	publication bias strongly suspected [d]	2051	2042	**8211.29** (2907,57; 23189,53)	⊕◯◯◯ Very low [d]	Important
Serotype 15B	not serious	very serious [d]	not serious	not serious	publication bias strongly suspected [d]	2051	2042	**1697.89** (437,73; 6585,9)	⊕◯◯◯ Very low [d]	Important
Serotype 22F	not serious	very serious [d]	not serious	not serious	publication bias strongly suspected [d]	2051	2042	**7781.5** (2830,94; 21389,26)	⊕◯◯◯ Very low [d]	Important
Serotype 33F	not serious	very serious [d]	not serious	not serious	publication bias strongly suspected [d]	2051	2042	**6692.72** (1902,78; 23540,57)	⊕◯◯◯ Very low [d]	Important

[a] *Heterogeneity not important*
[b] *Moderate heterogeneity*
[c] *High heterogeneity*
[d] *Very high heterogeneity*
[e] *All studies were randomized controlled trials*

	**Certainty Assessment**					**Patients, n**				
**Outcome [e]**	**Risk of Bias**	**Inconsistency**	**Indirectness**	**Imprecision**	**Other Considerations**	**PCV20**	**PCV13**	**OPA (95%-CI)**	**Certainty**	**Importance**
**OPA PCV20 after booster dose**										
Serotype 1	not serious	very serious [d]	not serious	not serious	publication bias strongly suspected [d]	2051	2042	**66.22** (20,34; 215,57)	⊕◯◯◯ Very low [d]	Important
Serotype 3	not serious	very serious [d]	not serious	not serious	publication bias strongly suspected [d]	2051	2042	**96.74** (54,41; 171,99)	⊕◯◯◯ Very low [d]	Important

Serotype 4	not serious	very serious [d]	not serious	not serious	publication bias strongly suspected [d]	2051	2042	**828.15** (374,97; 1829,06)	⊕◯◯◯ Very low [d]	Important
Serotype 5	not serious	very serious [d]	not serious	not serious	publication bias strongly suspected [d]	2051	2042	**77.47** (39,35; 152,51)	⊕◯◯◯ Very low [d]	Important
Serotype 6A	not serious	very serious [d]	not serious	not serious	publication bias strongly suspected [d]	2051	2042	**1728.47** (774,58; 3857,06)	⊕◯◯◯ Very low [d]	Important
Serotype 6B	not serious	very serious [d]	not serious	not serious	publication bias strongly suspected [d]	2051	2042	**1057.04** (340,56; 3280,8)	⊕◯◯◯ Very low [d]	Important
Serotype 7F	not serious	very serious [d]	not serious	not serious	publication bias strongly suspected [d]	2051	2042	**2568.52** (1309,23; 5039,07)	⊕◯◯◯ Very low [d]	Important
Serotype 9V	not serious	very serious [d]	not serious	not serious	publication bias strongly suspected [d]	2051	2042	**2611.25** (1156,76; 5894,6)	⊕◯◯◯ Very low [d]	Important
Serotype 14	not serious	very serious [d]	not serious	not serious	publication bias strongly suspected [d]	2051	2042	**981.53** (443,23; 2173,59)	⊕◯◯◯ Very low [d]	Important
Serotype 18C	not serious	very serious [d]	not serious	not serious	publication bias strongly suspected [d]	2051	2042	**2167.13** (974,25; 4820,62)	⊕◯◯◯ Very low [d]	Important
Serotype 19A	not serious	very serious [d]	not serious	not serious	publication bias strongly suspected [d]	2051	2042	**919.43** (460,95; 1833,95)	⊕◯◯◯ Very low [d]	Important
Serotype 19F	not serious	very serious [d]	not serious	not serious	publication bias strongly suspected [d]	2051	2042	**383.69** (116,63; 1262,22)	⊕◯◯◯ Very low [d]	Important
Serotype 23F	not serious	very serious [d]	not serious	not serious	publication bias strongly suspected [d]	2051	2042	**960.63** (415,01; 2223,55)	⊕◯◯◯ Very low [d]	Important
Serotype 8	not serious	very serious [d]	not serious	not serious	publication bias strongly suspected [d]	2051	2042	**1764.53** (893,39; 3485,1)	⊕◯◯◯ Very low [d]	Important
Serotype 10A	not serious	very serious [d]	not serious	not serious	publication bias strongly suspected [d]	2051	2042	**4080.91** (1877,4; 8870,67)	⊕◯◯◯ Very low [d]	Important
Serotype 11A	not serious	serious [c]	not serious	not serious	publication bias strongly suspected [c]	2051	2042	**3687.87** (2255,19; 6030,69)	⊕⊕◯◯ Low [c]	Important
Serotype 12F	not serious	very serious [d]	not serious	not serious	publication bias strongly suspected [d]	2051	2042	**9486.26** (4182,79; 21514,14)	⊕◯◯◯ Very low [d]	Important
Serotype 15B	not serious	very serious [d]	not serious	not serious	publication bias strongly suspected [d]	2051	2042	**3787.35** (1706,91; 8403,51)	⊕◯◯◯ Very low [d]	Important
Serotype 22F	not serious	very serious [d]	not serious	not serious	publication bias strongly suspected [d]	2051	2042	**11376.09** (4749,45; 27248,52)	⊕◯◯◯ Very low [d]	Important
Serotype 33F	not serious	serious [c]	not serious	not serious	publication bias strongly suspected [c]	2051	2042	**15180.25** (6124,22; 37627,67)	⊕⊕◯◯ Low [c]	Important

[a] Heterogeneity not important
[b] Moderate heterogeneity
[c] High heterogeneity

[d] *Very high heterogeneity*
[e] *All studies were randomized controlled trials*

	Certainty Assessment					Patients, n				
**Outcome [e]**	**Risk of Bias**	**Inconsistency**	**Indirectness**	**Imprecision**	**Other Considerations**	**PCV20**	**PCV13**	**OPA (95%-CI)**	**Certainty**	**Importance**
**OPA PCV13 after primary series**										
Serotype 1	not serious	very serious [d]	not serious	not serious	publication bias strongly suspected [d]	2051	2042	**42.01** (12,62; 139,82)	⊕◯◯◯ Very low [d]	Important
Serotype 3	not serious	very serious [d]	not serious	not serious	publication bias strongly suspected [d]	2051	2042	**71.83** (27,04; 190,81)	⊕◯◯◯ Very low [d]	Important
Serotype 4	not serious	very serious [d]	not serious	not serious	publication bias strongly suspected [d]	2051	2042	**527.81** (140,64; 1980,79)	⊕◯◯◯ Very low [d]	Important
Serotype 5	not serious	very serious [d]	not serious	not serious	publication bias strongly suspected [d]	2051	2042	**52.05** (15,62; 173,45)	⊕◯◯◯ Very low [d]	Important
Serotype 6A	not serious	very serious [d]	not serious	not serious	publication bias strongly suspected [d]	2051	2042	**1028.76** (257,14; 4115,91)	⊕◯◯◯ Very low [d]	Important
Serotype 6B	not serious	very serious [d]	not serious	not serious	publication bias strongly suspected [d]	2051	2042	**549.67** (69,52; 4346,33)	⊕◯◯◯ Very low [d]	Important
Serotype 7F	not serious	very serious [d]	not serious	not serious	publication bias strongly suspected [d]	2051	2042	**1589.29** (512,52; 4928,3)	⊕◯◯◯ Very low [d]	Important
Serotype 9V	not serious	very serious [d]	not serious	not serious	publication bias strongly suspected [d]	2051	2042	**705.09** (169,79; 2928,03)	⊕◯◯◯ Very low [d]	Important
Serotype 14	not serious	very serious [d]	not serious	not serious	publication bias strongly suspected [d]	2051	2042	**652.61** (148,67; 2864,62)	⊕◯◯◯ Very low [d]	Important
Serotype 18C	not serious	very serious [d]	not serious	not serious	publication bias strongly suspected [d]	2051	2042	**1551.85** (379,70; 6342,47)	⊕◯◯◯ Very low [d]	Important
Serotype 19A	not serious	very serious [d]	not serious	not serious	publication bias strongly suspected [d]	2051	2042	**181.23** (42,18; 778,61)	⊕◯◯◯ Very low [d]	Important
Serotype 19F	not serious	very serious [d]	not serious	not serious	publication bias strongly suspected [d]	2051	2042	**179.18** (47,56; 675,03)	⊕◯◯◯ Very low [d]	Important
Serotype 23F	not serious	very serious [d]	not serious	not serious	publication bias strongly suspected [d]	2051	2042	**317.62** (36,41; 2770,9)	⊕◯◯◯ Very low [d]	Important
Serotype 8	not serious	not serious [a]	not serious	not serious	publication bias strongly suspected [a]	2051	2042	**17.07** (15,72; 18,54)	⊕⊕⊕⊕ High [a]	Important
Serotype 10A	not serious	not serious	not serious	not serious	none	2051	2042	**38** (35,78; 40,36)	⊕⊕⊕⊕ High	Important

Serotype 11A	not serious	very serious [d]	not serious	not serious	publication bias strongly suspected [d]	2051	2042	**41.62** (19,51; 88,78)	⊕◯◯◯ Very low [d]	Important
Serotype 12F	not serious	not serious [b]	not serious	not serious	publication bias strongly suspected [b]	2051	2042	**25.32** (22,83; 28,09)	⊕⊕⊕◯ Moderate [b]	Important
Serotype 15B	not serious	not serious [a]	not serious	not serious	publication bias strongly suspected [a]	2051	2042	**18.01** (14,97; 21,67)	⊕⊕⊕⊕ High [a]	Important
Serotype 22F	not serious	not serious	not serious	not serious	none	2051	2042	**10.13** (9,14; 11,22)	⊕⊕⊕⊕ High	Important
Serotype 33F	not serious	serious [c]	not serious	not serious	publication bias strongly suspected [c]	2051	2042	**143.88** (61,42; 337,07)	⊕⊕◯◯ Low [c]	Important

[a] *Heterogeneity not important*
[b] *Moderate heterogeneity*
[c] *High heterogeneity*
[d] *Very high heterogeneity*
[e] *All studies were randomized controlled trials*

	Certainty Assessment					Patients, n				
**Outcome [e]**	**Risk of Bias**	**Inconsistency**	**Indirectness**	**Imprecision**	**Other Considerations**	**PCV20**	**PCV13**	**OPA (95%-CI)**	**Certainty**	**Importance**
**OPA PCV13 after booster dose**										
Serotype 1	not serious	very serious [d]	not serious	not serious	publication bias strongly suspected [d]	2051	2042	**124.81** (36,09; 431,69)	⊕◯◯◯ Very low [d]	Important
Serotype 3	not serious	very serious [d]	not serious	not serious	publication bias strongly suspected [d]	2051	2042	**129.21** (82; 203,58)	⊕◯◯◯ Very low [d]	Important
Serotype 4	not serious	serious [c]	not serious	not serious	publication bias strongly suspected [c]	2051	2042	**1079.03** (517,31; 2250,67)	⊕⊕◯◯ Low [c]	Important
Serotype 5	not serious	very serious [d]	not serious	not serious	publication bias strongly suspected [d]	2051	2042	**112.40** (45,44; 278,03)	⊕◯◯◯ Very low [d]	Important
Serotype 6A	not serious	very serious [d]	not serious	not serious	publication bias strongly suspected [d]	2051	2042	**2287.81** (855,41; 6118,78)	⊕◯◯◯ Very low [d]	Important
Serotype 6B	not serious	very serious [d]	not serious	not serious	publication bias strongly suspected [d]	2051	2042	**1698.38** (557,69; 5172,21)	⊕◯◯◯ Very low [d]	Important
Serotype 7F	not serious	very serious [d]	not serious	not serious	publication bias strongly suspected [d]	2051	2042	**3115.62** (1379,02; 7039,1)	⊕◯◯◯ Very low [d]	Important
Serotype 9V	not serious	serious [c]	not serious	not serious	publication bias strongly suspected [c]	2051	2042	**3570.38** (1851,41; 6885,35)	⊕⊕◯◯ Low [c]	Important
Serotype 14	not serious	very serious [d]	not serious	not serious	publication bias strongly suspected [d]	2051	2042	**1015.64** (503,57; 2048,4)	⊕◯◯◯ Very low [d]	Important

Serotype 18C	not serious	very serious [d]	not serious	not serious	publication bias strongly suspected [d]	2051	2042	**2972.14** (1278,3; 6910,46)	⊕◯◯◯ Very low [d]	Important
Serotype 19A	not serious	very serious [d]	not serious	not serious	publication bias strongly suspected [d]	2051	2042	**1211.03** (491,04; 2986,75)	⊕◯◯◯ Very low [d]	Important
Serotype 19F	not serious	very serious [d]	not serious	not serious	publication bias strongly suspected [d]	2051	2042	**600.25** (154,5; 2332,1)	⊕◯◯◯ Very low [d]	Important
Serotype 23F	not serious	very serious [d]	not serious	not serious	publication bias strongly suspected [d]	2051	2042	**1639.39** (637,6; 4215,22)	⊕◯◯◯ Very low [d]	Important
Serotype 8	not serious	not serious	not serious	not serious	none	2051	2042	**28.67** (23,63; 34,79)	⊕⊕⊕⊕ High	Important
Serotype 10A	not serious	not serious	not serious	not serious	none	2051	2042	**65.97** (50,77; 85,7)	⊕⊕⊕⊕ High	Important
Serotype 11A	not serious	not serious	not serious	not serious	none	2051	2042	**71.96** (57,98; 89,29)	⊕⊕⊕⊕ High	Important
Serotype 12F	not serious	not serious	not serious	not serious	publication bias strongly suspected [b]	2051	2042	**31.25** (23,73; 41,14)	⊕⊕⊕◯ Moderate [b]	Important
Serotype 15B	not serious	not serious	not serious	not serious	none	2051	2042	**25.09** (19,85; 31,72)	⊕⊕⊕⊕ High	Important
Serotype 22F	not serious	not serious	not serious	not serious	none	2051	2042	**16.4** (14,48; 18,57)	⊕⊕⊕⊕ High	Important
Serotype 33F	not serious	very serious [d]	not serious	not serious	publication bias strongly suspected [d]	2051	2042	**399.53** (121,78; 1310,71)	⊕◯◯◯ Very low [d]	Important

[a] *Heterogeneity not important*
[b] *Moderate heterogeneity*
[c] *High heterogeneity*
[d] *Very high heterogeneity*
[e] *All studies were randomized controlled trials*

Figures of risk of bias (S1-S6)

Figure S1. Risk of bias of RCTs for GMR after primary series

Study ID	Randomization process	Deviations from intended interventions	Missing outcome data	Measurement of the outcome	Selection of the reported result	Overall Bias
Korbal et all, 2024	+	+	+	+	+	+
Senders et all, 2024	+	+	+	+	+	+
Ishihara et all, 2024	+	+	+	+	+	

+	Low risk
?	Some concerns
„	High risk

Figure S2. Risk of bias of RCTs for DP after primary series.

Study ID	Randomization process	Deviations from intended interventions	Missing outcome data	Measurement of the outcome	Selection of the reported result	Overall Bias
Korbal et all, 2024	+	+	+	+	+	+
Senders et all, 2024	+	+	+	+	+	+
Ishihara et all, 2024	+	+	+	+	+	+

+	Low risk
?	Some concerns
„	High risk

Figure S3. Risk of bias of RCTs for GMR after booster dose.

Study ID	Randomization process	Deviations from intended interventions	Missing outcome data	Measurement of the outcome	Selection of the reported result	Overall Bias
Korbal et all, 2024	+	+	+	+	+	+
Senders et all, 2024	+	+	+	+	+	+

+ Low risk

? Some concerns

„ High risk

Figure S4. Risk of bias of RCTs for DP after booster dose.

Study ID	Randomization process	Deviations from intended interventions	Missing outcome data	Measurement of the outcome	Selection of the reported result	Overall Bias
Korbal et all, 2024	+	+	+	+	+	+
Senders et all, 2024	+	+	+	+	+	+

+	Low risk
?	Some concerns
„	High risk

Figure S5. Risk of bias of RCTs for OPA after primary series.

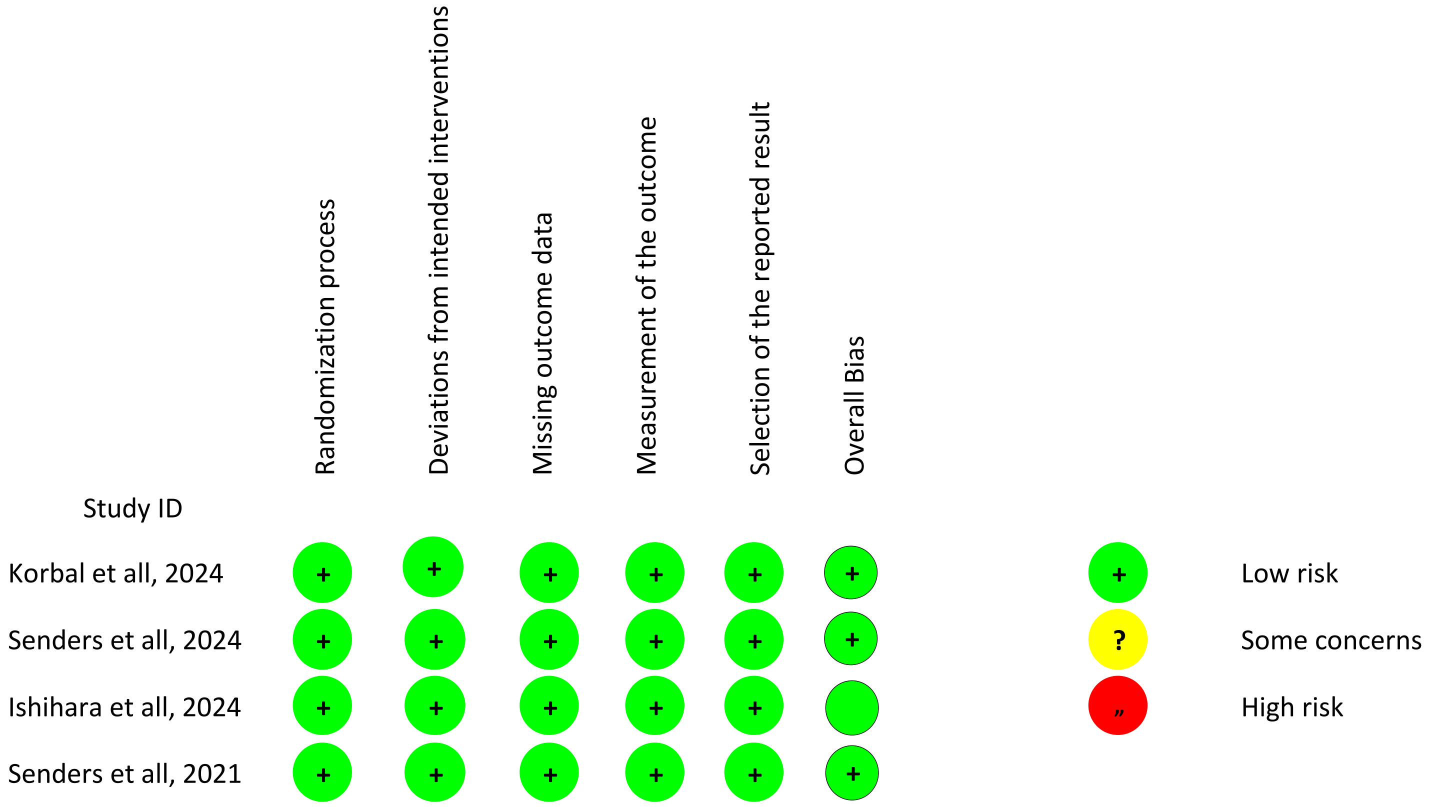

Figure S6. Risk of bias of RCTs for OPA after booster dose.

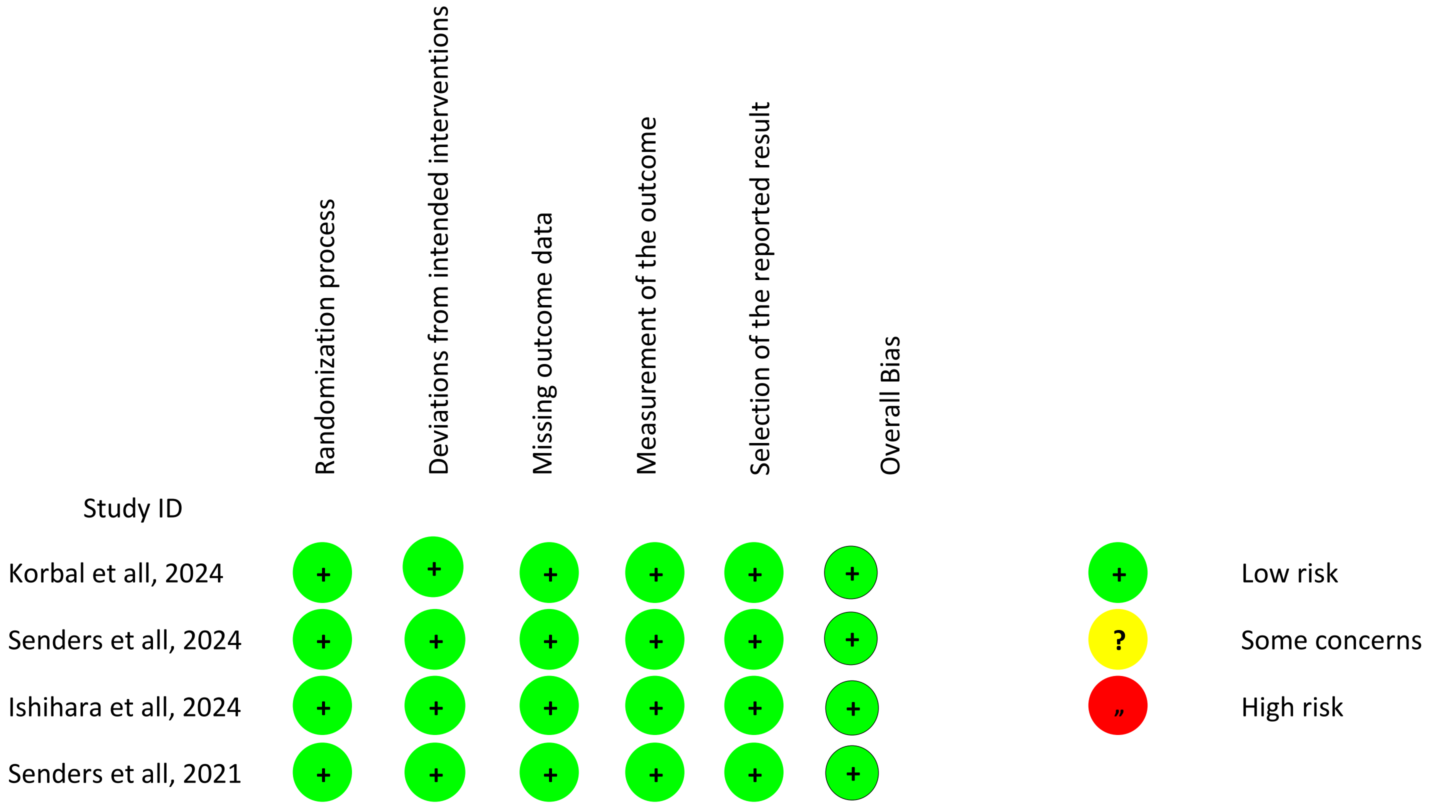

Figures of sensivity analysis for each serotype (S7-S14)

Figure S7. Sensivity analysis for GMR after primary series.

1

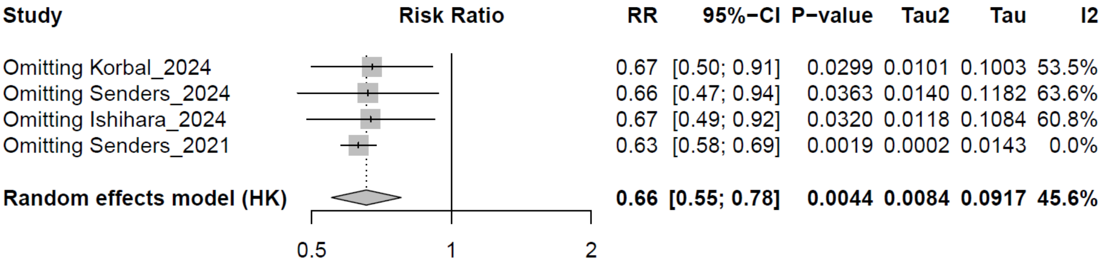

3

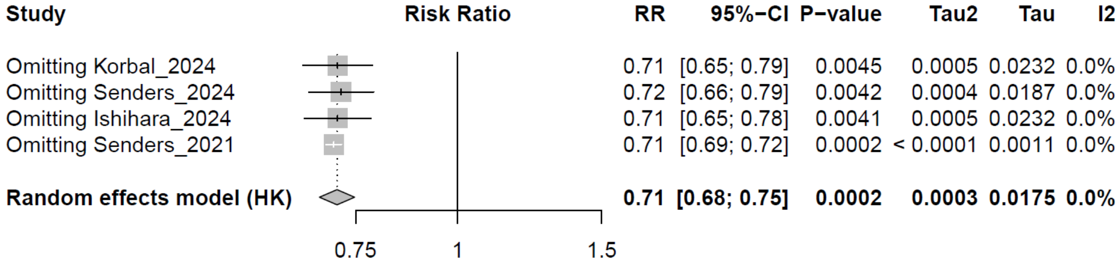

4

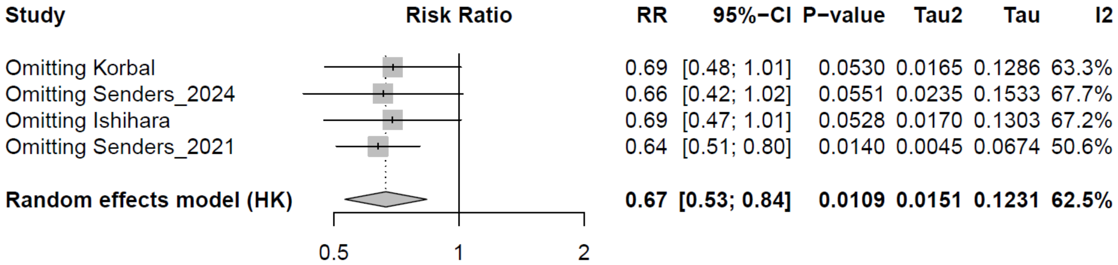

5

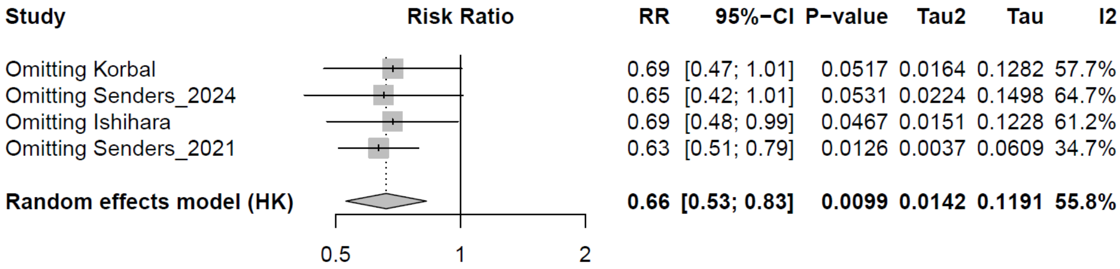

6A

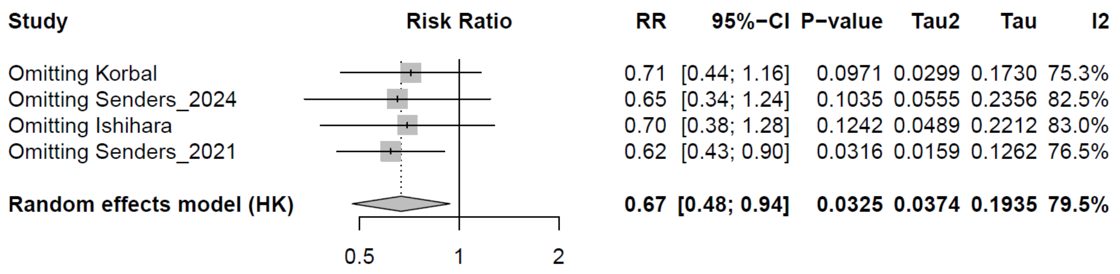
Study
Risk Ratio
RR 95%−CI P−value Tau2 Tau I2
Omitting Korbal 0.71 [0.44; 1.16] 0.0971 0.0299 0.1730 75.3%
Omitting Senders_2024 0.65 [0.34; 1.24] 0.1035 0.0555 0.2356 82.5%
Omitting Ishihara 0.70 [0.38; 1.28] 0.1242 0.0489 0.2212 83.0%
Omitting Senders_2021 0.62 [0.43; 0.90] 0.0316 0.0159 0.1262 76.5%
Random effects model (HK) 0.67 [0.48; 0.94] 0.0325 0.0374 0.1935 79.5%
0.5
1
2

6B

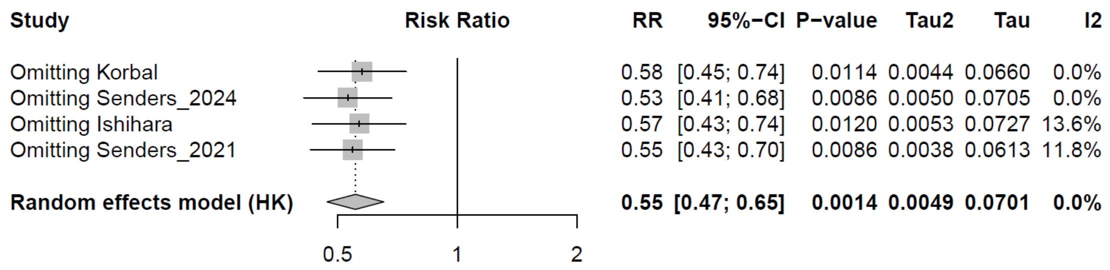
Study
Risk Ratio
RR 95%−CI P−value Tau2 Tau I2
Omitting Korbal 0.58 [0.45; 0.74] 0.0114 0.0044 0.0660 0.0%
Omitting Senders_2024 0.53 [0.41; 0.68] 0.0086 0.0050 0.0705 0.0%
Omitting Ishihara 0.57 [0.43; 0.74] 0.0120 0.0053 0.0727 13.6%
Omitting Senders_2021 0.55 [0.43; 0.70] 0.0086 0.0038 0.0613 11.8%
Random effects model (HK) 0.55 [0.47; 0.65] 0.0014 0.0049 0.0701 0.0%
0.5
1
2

7F

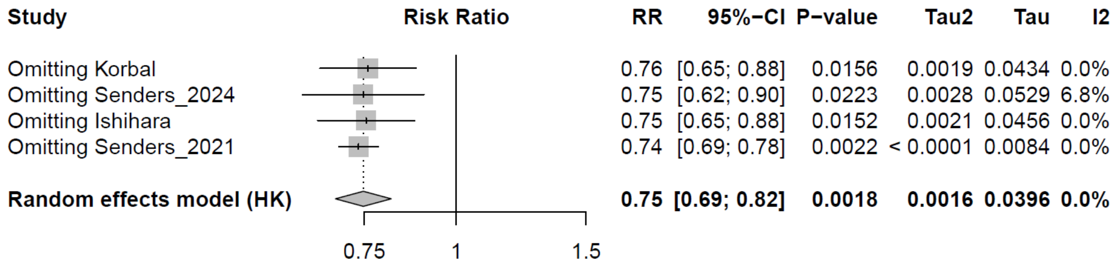
Study
Risk Ratio
RR 95%−CI P−value Tau2 Tau I2
Omitting Korbal 0.76 [0.65; 0.88] 0.0156 0.0019 0.0434 0.0%
Omitting Senders_2024 0.75 [0.62; 0.90] 0.0223 0.0028 0.0529 6.8%
Omitting Ishihara 0.75 [0.65; 0.88] 0.0152 0.0021 0.0456 0.0%
Omitting Senders_2021 0.74 [0.69; 0.78] 0.0022 < 0.0001 0.0084 0.0%
Random effects model (HK) 0.75 [0.69; 0.82] 0.0018 0.0016 0.0396 0.0%
0.75
1
1.5

9V

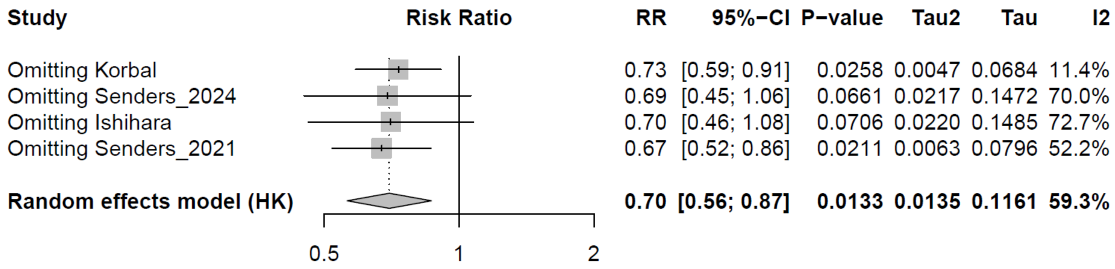
Study
Risk Ratio
RR 95%−CI P−value Tau2 Tau I2
Omitting Korbal 0.73 [0.59; 0.91] 0.0258 0.0047 0.0684 11.4%
Omitting Senders_2024 0.69 [0.45; 1.06] 0.0661 0.0217 0.1472 70.0%
Omitting Ishihara 0.70 [0.46; 1.08] 0.0706 0.0220 0.1485 72.7%
Omitting Senders_2021 0.67 [0.52; 0.86] 0.0211 0.0063 0.0796 52.2%
Random effects model (HK) 0.70 [0.56; 0.87] 0.0133 0.0135 0.1161 59.3%
0.5
1
2

14

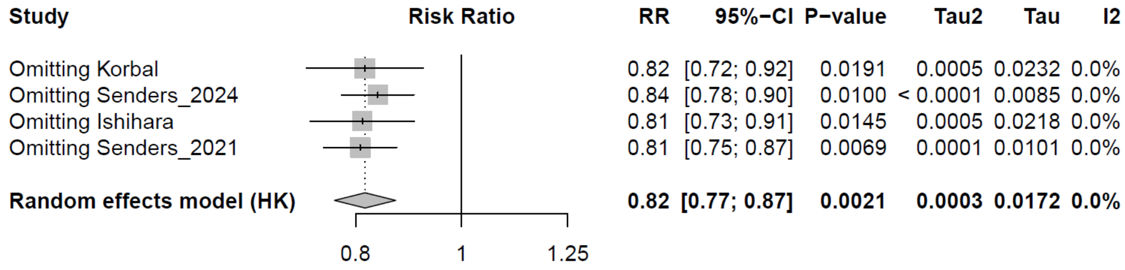
Study
Risk Ratio
RR 95%−CI P−value Tau2 Tau I2
Omitting Korbal 0.82 [0.72; 0.92] 0.0191 0.0005 0.0232 0.0%
Omitting Senders_2024 0.84 [0.78; 0.90] 0.0100 < 0.0001 0.0085 0.0%
Omitting Ishihara 0.81 [0.73; 0.91] 0.0145 0.0005 0.0218 0.0%
Omitting Senders_2021 0.81 [0.75; 0.87] 0.0069 0.0001 0.0101 0.0%
Random effects model (HK) 0.82 [0.77; 0.87] 0.0021 0.0003 0.0172 0.0%
0.8
1
1.25

18C

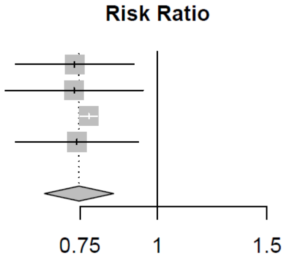

Study	RR	95%-CI	P-value	Tau2	Tau	I2
Omitting Korbal	0.74	[0.59; 0.92]	0.0265	0.0041	0.0637	36.1%
Omitting Senders_2024	0.73	[0.57; 0.95]	0.0350	0.0049	0.0698	33.3%
Omitting Ishihara	0.78	[0.75; 0.80]	0.0008	< 0.0001	0.0021	0.0%
Omitting Senders_2021	0.74	[0.59; 0.93]	0.0301	0.0050	0.0709	42.9%
**Random effects model (HK)**	**0.75**	**[0.66; 0.85]**	**0.0055**	**0.0032**	**0.0568**	**16.8%**

19A

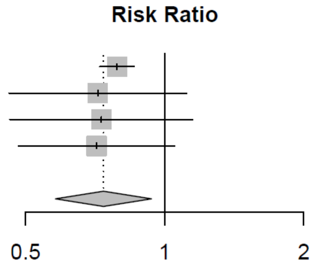

Study	RR	95%-CI	P-value	Tau2	Tau	I2
Omitting Korbal	0.79	[0.72; 0.86]	0.0070	0.0003	0.0185	0.0%
Omitting Senders_2024	0.72	[0.46; 1.12]	0.0838	0.0241	0.1552	78.6%
Omitting Ishihara	0.73	[0.46; 1.15]	0.0966	0.0271	0.1647	83.6%
Omitting Senders_2021	0.71	[0.48; 1.05]	0.0644	0.0194	0.1395	81.5%
**Random effects model (HK)**	**0.74**	**[0.58; 0.94]**	**0.0269**	**0.0173**	**0.1315**	**75.5%**

19F

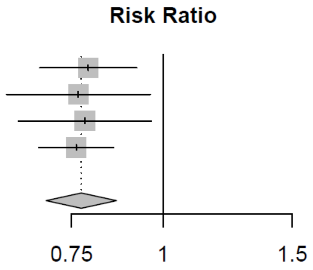

Study	RR	95%-CI	P-value	Tau2	Tau	I2
Omitting Korbal	0.79	[0.68; 0.92]	0.0218	0.0020	0.0453	0.0%
Omitting Senders_2024	0.77	[0.61; 0.96]	0.0366	0.0048	0.0691	31.5%
Omitting Ishihara	0.78	[0.63; 0.96]	0.0367	0.0043	0.0659	35.2%
Omitting Senders_2021	0.76	[0.68; 0.86]	0.0099	0.0008	0.0274	0.0%
**Random effects model (HK)**	**0.77**	**[0.69; 0.86]**	**0.0051**	**0.0029**	**0.0538**	**12.1%**

23F

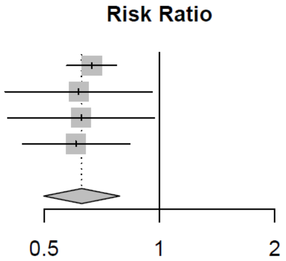

Study	RR	95%-CI	P-value	Tau2	Tau	I2
Omitting Korbal	0.67	[0.57; 0.77]	0.0072	0.0014	0.0375	0.0%
Omitting Senders_2024	0.62	[0.40; 0.96]	0.0421	0.0207	0.1437	63.3%
Omitting Ishihara	0.63	[0.40; 0.97]	0.0443	0.0221	0.1487	69.9%
Omitting Senders_2021	0.61	[0.44; 0.84]	0.0216	0.0104	0.1019	60.0%
**Random effects model (HK)**	**0.63**	**[0.50; 0.79]**	**0.0074**	**0.0134**	**0.1157**	**55.3%**

8

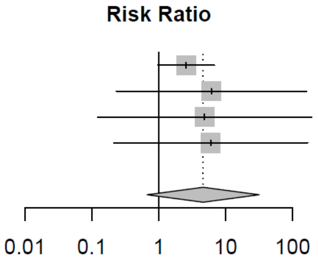

Study	RR	95%-CI	P-value	Tau2	Tau	I2
Omitting Korbal	2.56	[0.97; 6.78]	0.0533	0.1413	0.3759	95.9%
Omitting Senders_2024	6.10	[0.23; 160.52]	0.1405	1.7179	1.3107	99.5%
Omitting Ishihara	4.81	[0.12; 190.66]	0.2076	2.1820	1.4772	99.8%
Omitting Senders_2021	5.95	[0.21; 167.04]	0.1481	1.7951	1.3398	99.8%
**Random effects model (HK)**	**4.60**	**[0.67; 31.69]**	**0.0865**	**1.4608**	**1.2086**	**99.7%**

10A

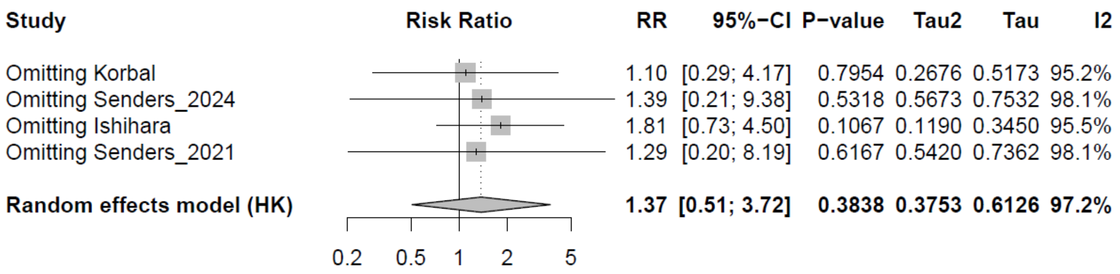

11A

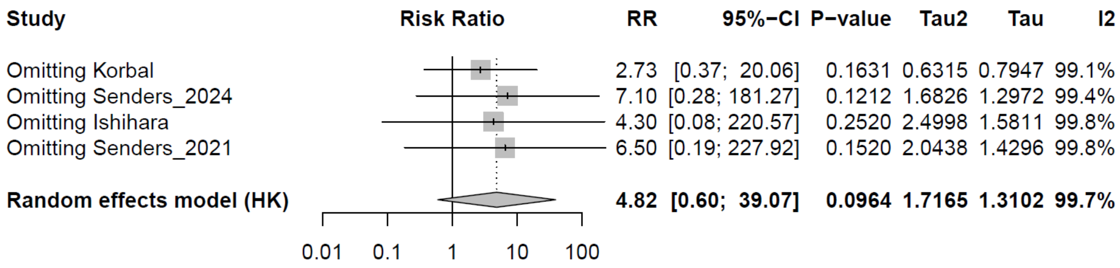

12F

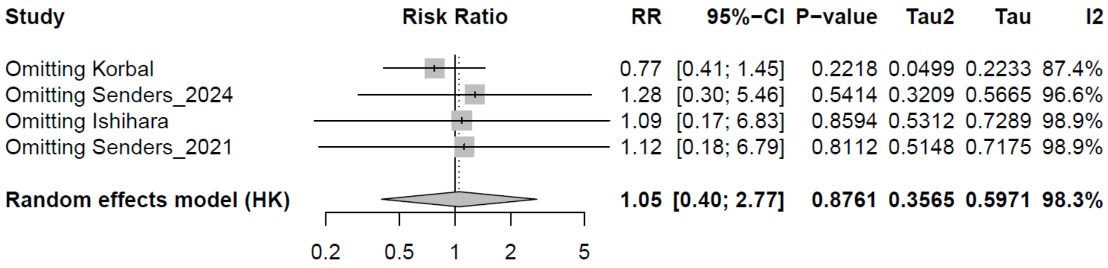

15B

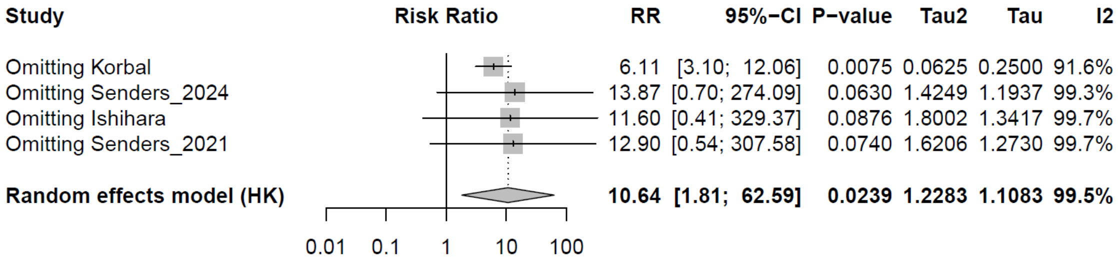

22F

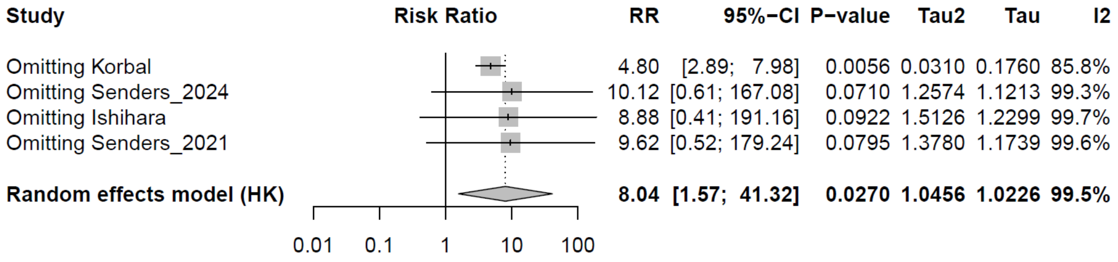

33F

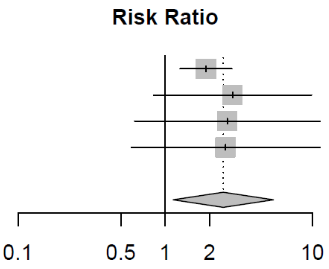

Study	RR	95%-CI	P-value	Tau2	Tau	I2
Omitting Korbal	1.89	[1.27; 2.84]	0.0209	0.0166	0.1287	68.2%
Omitting Senders_2024	2.87	[0.83; 9.88]	0.0669	0.2303	0.4799	96.3%
Omitting Ishihara	2.64	[0.62; 11.25]	0.1019	0.3259	0.5709	98.4%
Omitting Senders_2021	2.57	[0.59; 11.20]	0.1103	0.3419	0.5847	98.4%
**Random effects model (HK)**	**2.48**	**[1.13; 5.44]**	**0.0345**	**0.2306**	**0.4802**	**97.6%**

Figure S8. Sensivity analysis for GMR after booster dose.

1

Study	RR	95%−CI	P-value	Tau2	Tau	I2
Omitting Korbal, 2024	0.67	[0.54; 0.84]	0.0164	0.0045	0.0670	27.8%
Omitting Senders, 2024	0.66	[0.53; 0.82]	0.0147	0.0040	0.0634	17.5%
Omitting Ishihara_2024	0.69	[0.63; 0.75]	0.0028	0.0003	0.0181	0.0%
Omitting Senders_2021	0.66	[0.57; 0.77]	0.0076	0.0019	0.0438	0.0%
**Random effects model (HK)**	**0.67**	**[0.60; 0.75]**	**0.0014**	**0.0026**	**0.0505**	**0.0%**

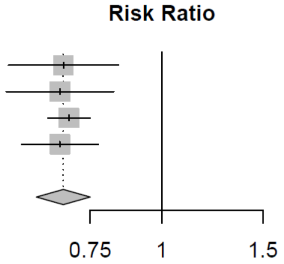

3

Study	RR	95%−CI	P-value	Tau2	Tau	I2
Omitting Korbal, 2024	0.68	[0.58; 0.80]	0.0100	0.0024	0.0495	0.0%
Omitting Senders, 2024	0.68	[0.57; 0.81]	0.0108	0.0024	0.0489	0.0%
Omitting Ishihara_2024	0.68	[0.57; 0.80]	0.0096	0.0029	0.0542	0.0%
Omitting Senders_2021	0.66	[0.65; 0.68]	0.0002	< 0.0001	0.0017	0.0%
**Random effects model (HK)**	**0.67**	**[0.62; 0.73]**	**0.0006**	**0.0017**	**0.0409**	**0.0%**

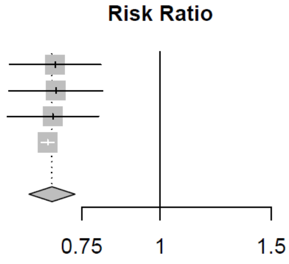

4

Study	RR	95%−CI	P-value	Tau2	Tau	I2
Omitting Korbal_2024	0.75	[0.65; 0.88]	0.0151	0.0012	0.0346	0.0%
Omitting Senders_2024	0.74	[0.65; 0.86]	0.0122	0.0008	0.0287	0.0%
Omitting Ishihara_2024	0.77	[0.75; 0.80]	0.0008	< 0.0001	0.0022	0.0%
Omitting Senders_2021	0.76	[0.66; 0.87]	0.0137	0.0014	0.0372	0.0%
**Random effects model (HK)**	**0.76**	**[0.70; 0.82]**	**0.0015**	**0.0008**	**0.0277**	**0.0%**

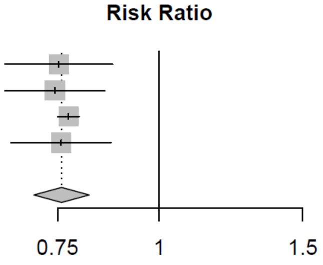

5

Study	RR	95%−CI	P-value	Tau2	Tau	I2
Omitting Korbal, 2024	0.70	[0.58; 0.85]	0.0155	0.0024	0.0488	13.3%
Omitting Senders, 2024	0.69	[0.59; 0.81]	0.0096	0.0012	0.0340	0.0%
Omitting Ishihara_2024	0.73	[0.68; 0.78]	0.0027	0.0002	0.0124	0.0%
Omitting Senders_2021	0.71	[0.59; 0.85]	0.0139	0.0027	0.0515	11.4%
**Random effects model (HK)**	**0.71**	**[0.64; 0.78]**	**0.0016**	**0.0015**	**0.0393**	**0.0%**

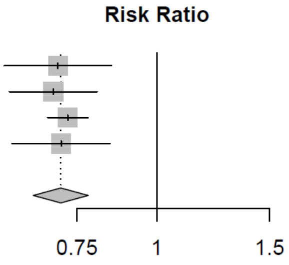

6A

Study	RR	95%-CI	P-value	Tau2	Tau	I2
Omitting Korbal, 2024	0.74	[0.64; 0.85]	0.0121	0.0010	0.0316	0.0%
Omitting Senders, 2024	0.69	[0.61; 0.78]	0.0056	0.0005	0.0231	0.0%
Omitting Ishihara_2024	0.72	[0.59; 0.89]	0.0212	0.0032	0.0562	38.1%
Omitting Senders_2021	0.71	[0.58; 0.88]	0.0197	0.0035	0.0589	45.8%
**Random effects model (HK)**	**0.72**	**[0.64; 0.81]**	**0.0028**	**0.0021**	**0.0463**	**19.0%**

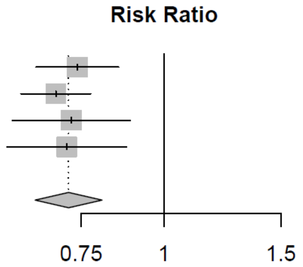

6B

Study	RR	95%-CI	P-value	Tau2	Tau	I2
Omitting Korbal, 2024	0.69	[0.64; 0.74]	0.0019	< 0.0001	0.0093	0.0%
Omitting Senders, 2024	0.63	[0.49; 0.81]	0.0160	0.0041	0.0642	18.7%
Omitting Ishihara_2024	0.64	[0.49; 0.85]	0.0206	0.0061	0.0779	47.6%
Omitting Senders_2021	0.65	[0.50; 0.86]	0.0219	0.0073	0.0853	50.9%
**Random effects model (HK)**	**0.66**	**[0.56; 0.76]**	**0.0031**	**0.0043**	**0.0652**	**26.4%**

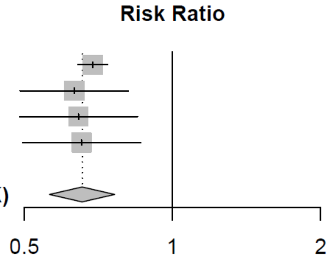

7F

Study	RR	95%-CI	P-value	Tau2	Tau	I2
Omitting Korbal, 2024	0.71	[0.58; 0.87]	0.0182	0.0032	0.0569	45.6%
Omitting Senders, 2024	0.70	[0.61; 0.80]	0.0081	0.0010	0.0320	0.0%
Omitting Ishihara_2024	0.73	[0.62; 0.85]	0.0130	0.0024	0.0487	19.0%
Omitting Senders_2021	0.73	[0.64; 0.84]	0.0097	0.0015	0.0387	3.6%
**Random effects model (HK)**	**0.72**	**[0.65; 0.80]**	**0.0019**	**0.0021**	**0.0459**	**18.6%**

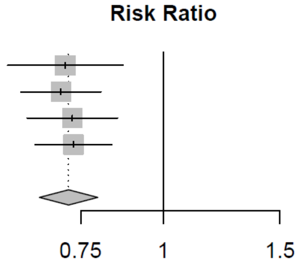

9V

Study	RR	95%-CI	P-value	Tau2	Tau	I2
Omitting Korbal, 2024	0.75	[0.60; 0.93]	0.0279	0.0034	0.0583	38.1%
Omitting Senders, 2024	0.72	[0.66; 0.77]	0.0031	0.0001	0.0110	0.0%
Omitting Ishihara_2024	0.76	[0.65; 0.88]	0.0160	0.0016	0.0398	10.5%
Omitting Senders_2021	0.75	[0.62; 0.90]	0.0212	0.0030	0.0550	39.1%
**Random effects model (HK)**	**0.74**	**[0.67; 0.83]**	**0.0032**	**0.0020**	**0.0449**	**17.2%**

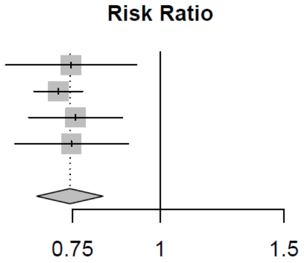

14

Study	RR	95%-CI	P-value	Tau2	Tau	I2
Omitting Korbal, 2024	0.85	[0.70; 1.03]	0.0677	0.0024	0.0494	9.3%
Omitting Senders, 2024	0.80	[0.77; 0.82]	0.0009	< 0.0001	0.0016	0.0%
Omitting Ishihara_2024	0.84	[0.70; 1.02]	0.0641	0.0025	0.0499	15.6%
Omitting Senders_2021	0.84	[0.71; 1.01]	0.0550	0.0021	0.0453	15.2%
**Random effects model (HK)**	**0.84**	**[0.75; 0.94]**	**0.0147**	**0.0017**	**0.0411**	**0.0%**

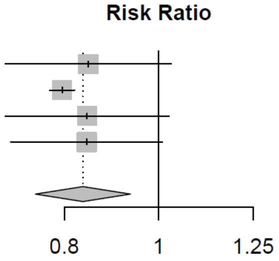

18C

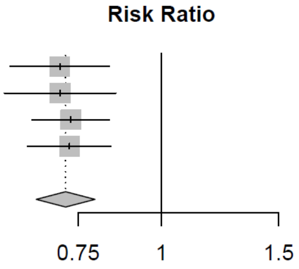

Study	RR	95%-CI	P-value	Tau2	Tau	I2
Omitting Korbal, 2024	0.70	[0.59; 0.84]	0.0126	0.0016	0.0400	0.0%
Omitting Senders, 2024	0.70	[0.58; 0.85]	0.0160	0.0022	0.0466	2.5%
Omitting Ishihara_2024	0.73	[0.64; 0.84]	0.0098	0.0014	0.0381	0.0%
Omitting Senders_2021	0.73	[0.63; 0.84]	0.0109	0.0016	0.0401	0.0%
**Random effects model (HK)**	**0.72**	**[0.65; 0.79]**	**0.0019**	**0.0018**	**0.0426**	**0.0%**

19A

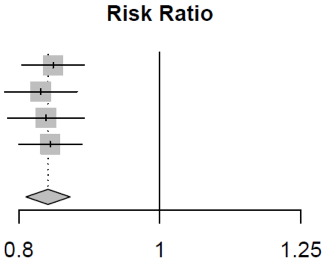

Study	RR	95%-CI	P-value	Tau2	Tau	I2
Omitting Korbal, 2024	0.84	[0.80; 0.89]	0.0046	< 0.0001	0.0063	0.0%
Omitting Senders, 2024	0.83	[0.78; 0.88]	0.0050	< 0.0001	0.0057	0.0%
Omitting Ishihara_2024	0.84	[0.79; 0.89]	0.0060	< 0.0001	0.0072	0.0%
Omitting Senders_2021	0.84	[0.80; 0.88]	0.0044	< 0.0001	0.0053	0.0%
**Random effects model (HK)**	**0.84**	**[0.81; 0.87]**	**0.0005**	**< 0.0001**	**0.0066**	**0.0%**

19F

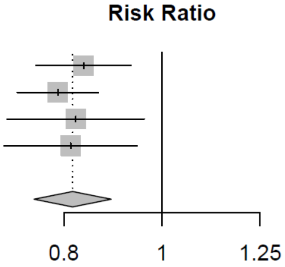

Study	RR	95%-CI	P-value	Tau2	Tau	I2
Omitting Korbal, 2024	0.84	[0.75; 0.93]	0.0192	0.0003	0.0186	0.0%
Omitting Senders, 2024	0.79	[0.72; 0.87]	0.0081	0.0002	0.0156	0.0%
Omitting Ishihara_2024	0.82	[0.70; 0.96]	0.0324	0.0012	0.0353	0.0%
Omitting Senders_2021	0.81	[0.70; 0.95]	0.0274	0.0013	0.0361	0.0%
**Random effects model (HK)**	**0.82**	**[0.75; 0.89]**	**0.0052**	**0.0008**	**0.0289**	**0.0%**

23F

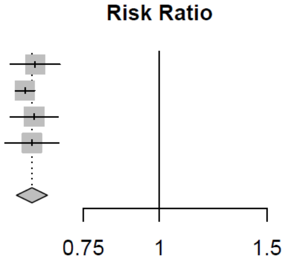

Study	RR	95%-CI	P-value	Tau2	Tau	I2
Omitting Korbal, 2024	0.62	[0.57; 0.69]	0.0022	0.0002	0.0127	0.0%
Omitting Senders, 2024	0.60	[0.58; 0.62]	0.0003	< 0.0001	0.0025	0.0%
Omitting Ishihara_2024	0.62	[0.57; 0.68]	0.0020	0.0001	0.0120	0.0%
Omitting Senders_2021	0.62	[0.56; 0.68]	0.0024	0.0003	0.0168	0.0%
**Random effects model (HK)**	**0.62**	**[0.58; 0.65]**	**0.0001**	**0.0002**	**0.0123**	**0.0%**

8

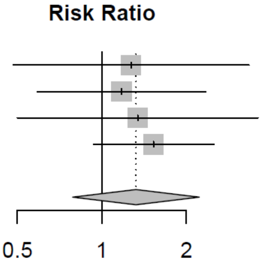

Study	RR	95%-CI	P-value	Tau2	Tau	I2
Omitting Korbal, 2024	1.27	[0.48; 3.34]	0.3986	0.1442	0.3798	97.0%
Omitting Senders, 2024	1.18	[0.59; 2.35]	0.4217	0.0698	0.2642	92.6%
Omitting Ishihara_2024	1.34	[0.50; 3.60]	0.3292	0.1513	0.3889	96.8%
Omitting Senders_2021	1.53	[0.93; 2.50]	0.0662	0.0342	0.1850	90.5%
**Random effects model (HK)**	**1.32**	**[0.79; 2.22]**	**0.1853**	**0.1003**	**0.3168**	**95.6%**

10A

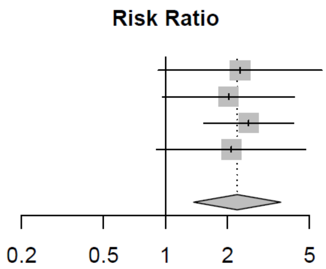

Study	RR	95%-CI	P-value	Tau2	Tau	I2
Omitting Korbal, 2024	2.30	[0.92; 5.73]	0.0592	0.1257	0.3546	95.3%
Omitting Senders, 2024	2.02	[0.97; 4.22]	0.0544	0.0780	0.2792	90.3%
Omitting Ishihara_2024	2.53	[1.53; 4.18]	0.0154	0.0341	0.1846	89.8%
Omitting Senders_2021	2.09	[0.91; 4.80]	0.0630	0.1056	0.3249	95.8%
**Random effects model (HK)**	**2.23**	**[1.37; 3.63]**	**0.0136**	**0.0863**	**0.2937**	**94.0%**

11A

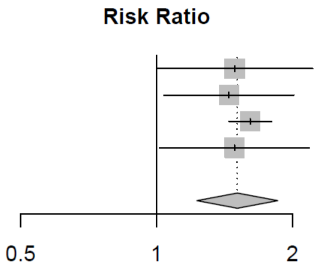

Study	RR	95%-CI	P-value	Tau2	Tau	I2
Omitting Korbal, 2024	1.49	[1.00; 2.22]	0.0495	0.0189	0.1374	78.5%
Omitting Senders, 2024	1.45	[1.04; 2.01]	0.0406	0.0116	0.1076	62.7%
Omitting Ishihara_2024	1.61	[1.45; 1.80]	0.0028	0.0004	0.0196	0.0%
Omitting Senders_2021	1.49	[1.02; 2.18]	0.0461	0.0185	0.1360	78.5%
**Random effects model (HK)**	**1.51**	**[1.23; 1.86]**	**0.0079**	**0.0120**	**0.1095**	**67.8%**

12F

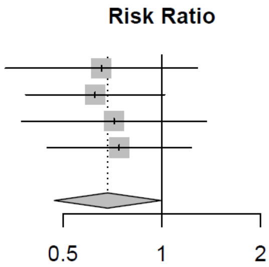

Study	RR	95%-CI	P-value	Tau2	Tau	I2
Omitting Korbal, 2024	0.65	[0.33; 1.28]	0.1138	0.0655	0.2560	93.5%
Omitting Senders, 2024	0.63	[0.39; 1.02]	0.0539	0.0305	0.1746	85.0%
Omitting Ishihara_2024	0.72	[0.37; 1.37]	0.1572	0.0611	0.2471	91.1%
Omitting Senders_2021	0.74	[0.45; 1.23]	0.1256	0.0351	0.1872	87.4%
**Random effects model (HK)**	**0.68**	**[0.47; 0.99]**	**0.0482**	**0.0487**	**0.2207**	**90.4%**

15B

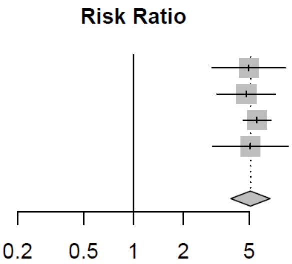

Study	RR	95%-CI	P-value	Tau2	Tau	I2
Omitting Korbal, 2024	4.96	[2.98; 8.26]	0.0054	0.0355	0.1883	89.6%
Omitting Senders, 2024	4.80	[3.19; 7.22]	0.0036	0.0205	0.1433	80.5%
Omitting Ishihara_2024	5.56	[4.59; 6.73]	0.0007	0.0030	0.0552	33.6%
Omitting Senders_2021	5.07	[3.02; 8.53]	0.0055	0.0383	0.1958	89.6%
**Random effects model (HK)**	**5.09**	**[3.87; 6.69]**	**0.0003**	**0.0244**	**0.1563**	**84.6%**

22F

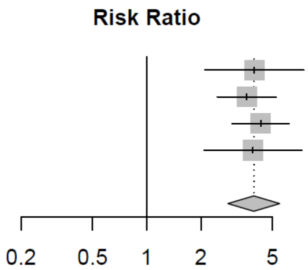

Study	RR	95%-CI	P-value	Tau2	Tau	I2
Omitting Korbal, 2024	3.96	[2.10; 7.47]	0.0112	0.0572	0.2393	93.1%
Omitting Senders, 2024	3.60	[2.47; 5.25]	0.0046	0.0163	0.1275	71.6%
Omitting Ishihara_2024	4.31	[2.99; 6.20]	0.0034	0.0158	0.1259	83.6%
Omitting Senders_2021	3.90	[2.09; 7.27]	0.0111	0.0572	0.2392	93.6%
**Random effects model (HK)**	**3.94**	**[2.83; 5.49]**	**0.0010**	**0.0373**	**0.1930**	**90.4%**

33F

Study	RR	95%-CI	P-value	Tau2	Tau	I2
Omitting Korbal, 2024	2.34	[0.51; 10.72]	0.1390	0.3689	0.6074	98.8%
Omitting Senders, 2024	1.97	[0.78; 4.95]	0.0869	0.1291	0.3593	95.5%
Omitting Ishihara_2024	2.48	[0.54; 11.37]	0.1249	0.3698	0.6081	98.7%
Omitting Senders_2021	2.96	[1.22; 7.19]	0.0341	0.1214	0.3484	97.2%
**Random effects model (HK)**	**2.41**	**[1.08; 5.38]**	**0.0398**	**0.2480**	**0.4980**	**98.2%**

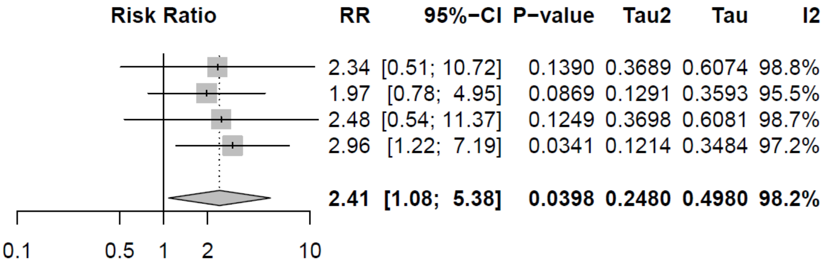

Figure S9. Sensivity analysis for DP after primary series.

1

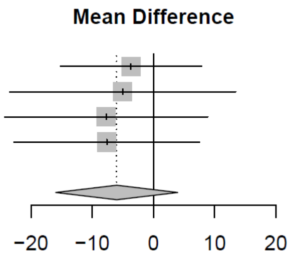

Study	MD	95%−CI	P−value	Tau2	Tau	I2
Omitting Korbal, 2024	−3.67	[−15.21; 7.87]	0.3048	15.9867	3.9983	82.1%
Omitting Senders, 2024	−5.03	[−23.50; 13.44]	0.3621	46.9224	6.8500	89.8%
Omitting Ishihara, 2024	−7.71	[−24.28; 8.86]	0.1833	36.2437	6.0203	79.9%
Omitting Senders_2021	−7.62	[−22.73; 7.48]	0.1620	32.1497	5.6701	90.7%
**Random effects model (HK)**	**−5.99**	**[−15.96; 3.97]**	**0.1514**	**33.0426**	**5.7483**	**87.6%**

3

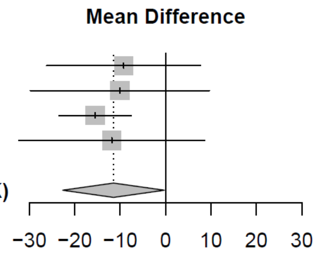

Study	MD	95%−CI	P−value	Tau2	Tau	I2
Omitting Korbal, 2024	−9.22	[−26.16; 7.72]	0.1440	36.1281	6.0107	90.2%
Omitting Senders, 2024	−10.05	[−29.76; 9.65]	0.1593	51.0638	7.1459	91.4%
Omitting Ishihara, 2024	−15.52	[−23.48; −7.55]	0.0139	5.6713	2.3815	0.0%
Omitting Senders_2021	−11.83	[−32.29; 8.63]	0.1307	61.1035	7.8169	94.0%
**Random effects model (HK)**	**−11.46**	**[−22.66; −0.26]**	**0.0473**	**39.9994**	**6.3245**	**91.0%**

4

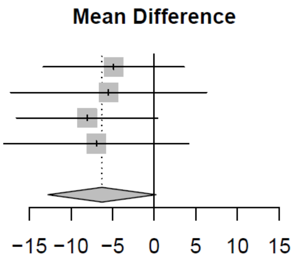

Study	MD	95%−CI	P−value	Tau2	Tau	I2
Omitting Korbal, 2024	−4.87	[−13.33; 3.59]	0.1316	6.9733	2.6407	70.0%
Omitting Senders, 2024	−5.49	[−17.25; 6.27]	0.1824	15.8439	3.9804	76.3%
Omitting Ishihara, 2024	−8.06	[−16.54; 0.42]	0.0549	7.1657	2.6769	35.9%
Omitting Senders_2021	−6.95	[−18.07; 4.16]	0.1148	15.4576	3.9316	82.1%
**Random effects model (HK)**	**−6.27**	**[−12.77; 0.22]**	**0.0544**	**11.7224**	**3.4238**	**74.2%**

5

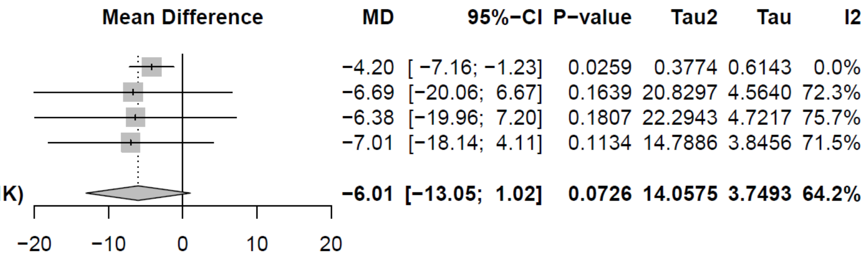

Study	MD	95%−CI	P−value	Tau2	Tau	I2
Omitting Korbal, 2024	−4.20	[−7.16; −1.23]	0.0259	0.3774	0.6143	0.0%
Omitting Senders, 2024	−6.69	[−20.06; 6.67]	0.1639	20.8297	4.5640	72.3%
Omitting Ishihara, 2024	−6.38	[−19.96; 7.20]	0.1807	22.2943	4.7217	75.7%
Omitting Senders_2021	−7.01	[−18.14; 4.11]	0.1134	14.7886	3.8456	71.5%
**Random effects model (HK)**	**−6.01**	**[−13.05; 1.02]**	**0.0726**	**14.0575**	**3.7493**	**64.2%**

6A

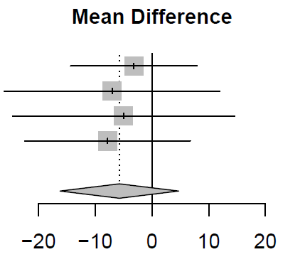

Study	MD	95%−CI	P−value	Tau2	Tau	I2
Omitting Korbal, 2024	−3.20	[−14.35; 7.95]	0.3426	15.4357	3.9288	73.2%
Omitting Senders, 2024	−7.02	[−26.03; 11.99]	0.2530	49.6971	7.0496	87.0%
Omitting Ishihara, 2024	−5.00	[−24.56; 14.56]	0.3859	54.5749	7.3875	89.1%
Omitting Senders_2021	−7.82	[−22.37; 6.74]	0.1470	28.6707	5.3545	88.8%
**Random effects model (HK)**	**−5.75**	**[−16.21; 4.71]**	**0.1787**	**37.1645**	**6.0963**	**86.3%**

6B

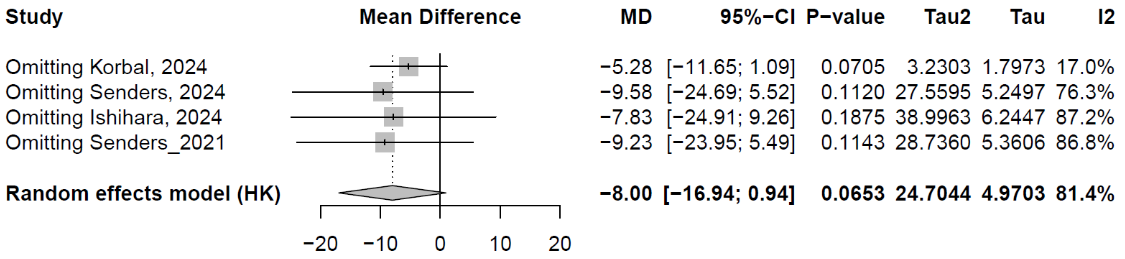

7F

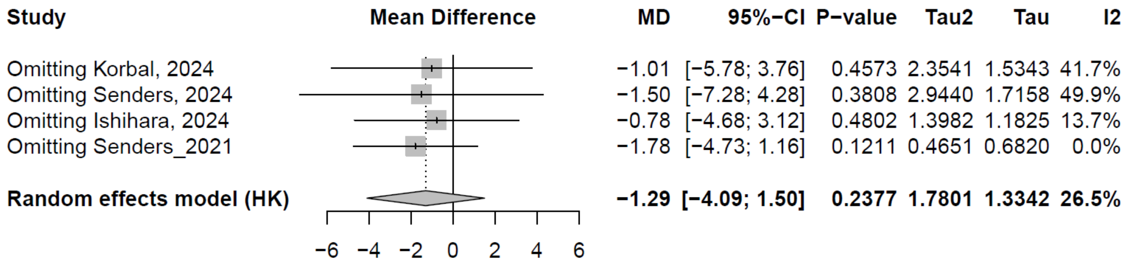

9V

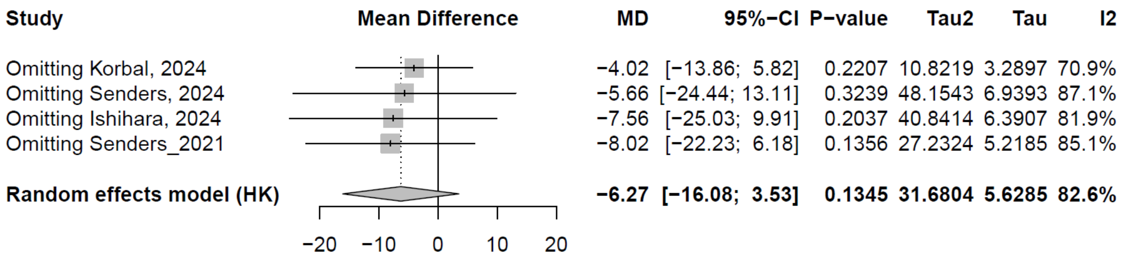

14

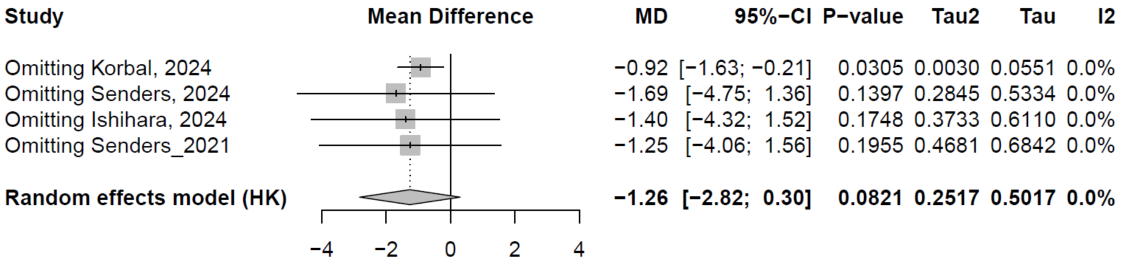

18C

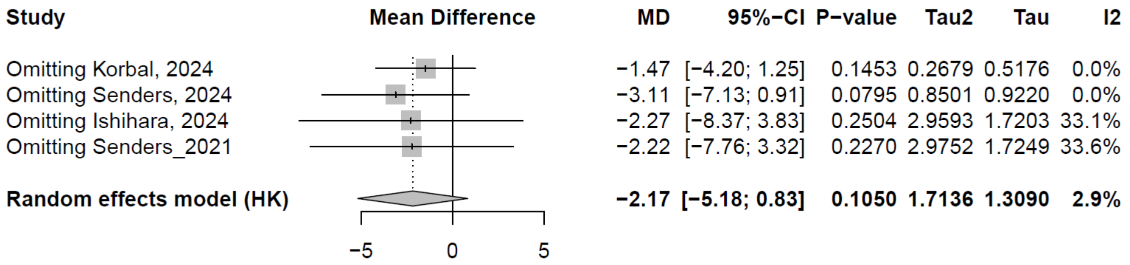

19A

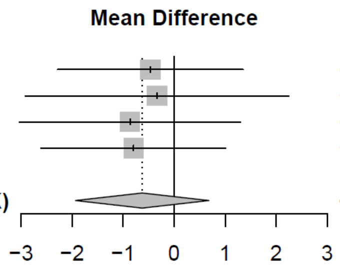

Study	MD	95%-CI	P-value	Tau2	Tau	I2
Omitting Korbal, 2024	−0.46	[−2.28; 1.35]	0.3864	0.1400	0.3742	0.0%
Omitting Senders, 2024	−0.33	[−2.91; 2.25]	0.6357	0.3602	0.6001	0.0%
Omitting Ishihara, 2024	−0.86	[−3.03; 1.30]	0.2286	0.3161	0.5622	0.0%
Omitting Senders_2021	−0.80	[−2.61; 1.01]	0.1986	0.1718	0.4145	0.0%
**Random effects model (HK)**	**−0.63**	**[−1.93; 0.68]**	**0.2254**	**0.2552**	**0.5052**	**0.0%**

19F

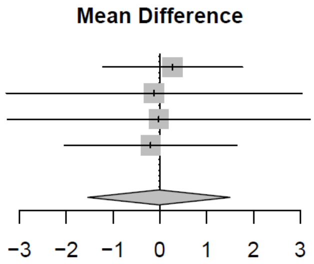

Study	MD	95%-CI	P-value	Tau2	Tau	I2
Omitting Korbal, 2024	0.27	[−1.22; 1.76]	0.5150	0.1319	0.3632	0.0%
Omitting Senders, 2024	−0.12	[−3.28; 3.04]	0.8828	0.8428	0.9181	0.0%
Omitting Ishihara, 2024	−0.03	[−3.26; 3.21]	0.9765	0.8632	0.9291	0.0%
Omitting Senders_2021	−0.20	[−2.04; 1.64]	0.6854	0.1996	0.4467	0.0%
**Random effects model (HK)**	**−0.02**	**[−1.54; 1.50]**	**0.9685**	**0.4800**	**0.6928**	**0.0%**

23F

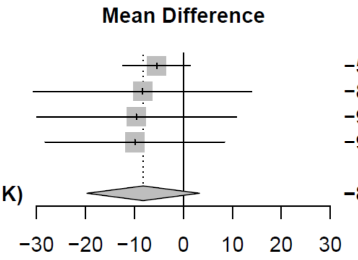

Study	MD	95%-CI	P-value	Tau2	Tau	I2
Omitting Korbal, 2024	−5.47	[−12.36; 1.41]	0.0758	3.3145	1.8206	7.6%
Omitting Senders, 2024	−8.33	[−30.62; 13.96]	0.2490	67.2455	8.2003	88.7%
Omitting Ishihara, 2024	−9.59	[−29.95; 10.77]	0.1799	55.7864	7.4690	86.2%
Omitting Senders_2021	−9.91	[−28.19; 8.37]	0.1450	46.2365	6.7997	87.0%
**Random effects model (HK)**	**−8.21**	**[−19.70; 3.28]**	**0.1075**	**42.8304**	**6.5445**	**83.5%**

8

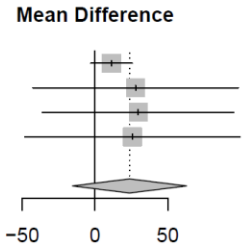

Study	MD	95%-CI	P-value	Tau2	Tau	I2
Omitting Korbal, 2024	11.24	[−3.03; 25.51]	0.0771	27.4473	5.2390	83.8%
Omitting Senders, 2024	27.84	[−42.74; 98.42]	0.2318	799.2604	28.2712	99.5%
Omitting Ishihara, 2024	29.60	[−36.12; 95.32]	0.1922	692.4730	26.3149	99.5%
Omitting Senders_2021	25.64	[−48.27; 99.55]	0.2741	879.9489	29.6639	99.5%
**Random effects model (HK)**	**23.66**	**[−15.54; 62.86]**	**0.1505**	**600.8463**	**24.5122**	**99.3%**

10A

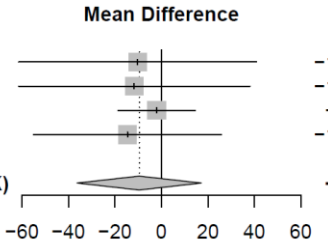

Study	MD	95%-CI	P-value	Tau2	Tau	I2
Omitting Korbal, 2024	−10.23	[−61.35; 40.90]	0.4802	407.1475	20.1779	97.0%
Omitting Senders, 2024	−11.70	[−61.43; 38.03]	0.4180	382.6127	19.5605	96.5%
Omitting Ishihara, 2024	−2.06	[−18.62; 14.50]	0.6455	35.1854	5.9317	75.3%
Omitting Senders_2021	−14.60	[−55.00; 25.81]	0.2603	251.9590	15.8732	96.3%
**Random effects model (HK)**	**−9.55**	**[−36.29; 17.19]**	**0.3383**	**269.1123**	**16.4046**	**95.5%**

11A

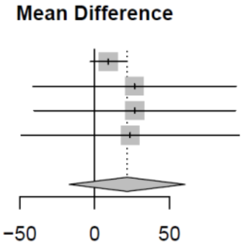

Study	MD	95%-CI	P-value	Tau2	Tau	I2
Omitting Korbal, 2024	9.10	[-2.82; 21.02]	0.0815	18.0915	4.2534	70.1%
Omitting Senders, 2024	26.54	[-41.32; 94.40]	0.2345	737.7762	27.1620	99.4%
Omitting Ishihara, 2024	26.77	[-40.45; 93.99]	0.2288	723.9763	26.9068	99.4%
Omitting Senders_2021	23.67	[-49.23; 96.57]	0.2973	855.7374	29.2530	99.5%
**Random effects model (HK)**	**21.66**	**[-17.05; 60.36]**	**0.1730**	**585.1013**	**24.1889**	**99.3%**

12F

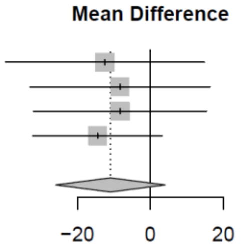

Study	MD	95%-CI	P-value	Tau2	Tau	I2
Omitting Korbal, 2024	-12.46	[-39.69; 14.77]	0.1878	105.6619	10.2792	88.9%
Omitting Senders, 2024	-8.31	[-32.95; 16.32]	0.2837	83.1280	9.1175	86.9%
Omitting Ishihara, 2024	-8.30	[-31.93; 15.33]	0.2699	78.3448	8.8513	91.1%
Omitting Senders_2021	-14.43	[-32.11; 3.25]	0.0724	41.3183	6.4279	85.2%
**Random effects model (HK)**	**-10.93**	**[-25.94; 4.08]**	**0.1032**	**77.5299**	**8.8051**	**88.5%**

15B

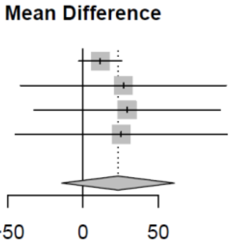

Study	MD	95%-CI	P-value	Tau2	Tau	I2
Omitting Korbal, 2024	11.48	[-2.78; 25.75]	0.0742	27.2639	5.2215	85.7%
Omitting Senders, 2024	26.81	[-41.48; 95.09]	0.2333	747.1410	27.3339	99.4%
Omitting Ishihara, 2024	29.20	[-32.60; 91.00]	0.1791	611.1172	24.7208	99.3%
Omitting Senders_2021	25.30	[-45.07; 95.68]	0.2619	797.0471	28.2320	99.5%
**Random effects model (HK)**	**23.26**	**[-14.16; 60.68]**	**0.1423**	**546.5109**	**23.3776**	**99.2%**

22F

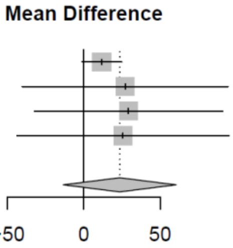

Study	MD	95%-CI	P-value	Tau2	Tau	I2
Omitting Korbal, 2024	11.74	[-1.34; 24.81]	0.0610	22.5280	4.7464	84.8%
Omitting Senders, 2024	27.10	[-40.32; 94.52]	0.2259	728.4992	26.9907	99.4%
Omitting Ishihara, 2024	29.23	[-32.48; 90.95]	0.1784	609.4586	24.6872	99.3%
Omitting Senders_2021	25.63	[-43.91; 95.17]	0.2537	778.4446	27.9006	99.5%
**Random effects model (HK)**	**23.50**	**[-13.53; 60.54]**	**0.1367**	**535.4855**	**23.1406**	**99.2%**

33F

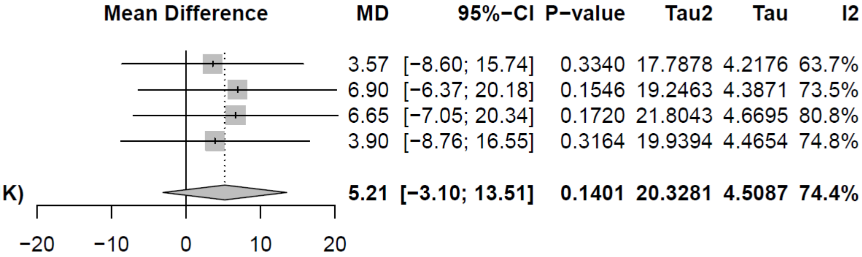

Study	MD	95%-CI	P-value	Tau2	Tau	I2
Omitting Korbal, 2024	3.57	[-8.60; 15.74]	0.3340	17.7878	4.2176	63.7%
Omitting Senders, 2024	6.90	[-6.37; 20.18]	0.1546	19.2463	4.3871	73.5%
Omitting Ishihara, 2024	6.65	[-7.05; 20.34]	0.1720	21.8043	4.6695	80.8%
Omitting Senders_2021	3.90	[-8.76; 16.55]	0.3164	19.9394	4.4654	74.8%
**Random effects model (HK)**	**5.21**	**[-3.10; 13.51]**	**0.1401**	**20.3281**	**4.5087**	**74.4%**

Figure S10. Sensivity analysis for DP after booster dose.

1

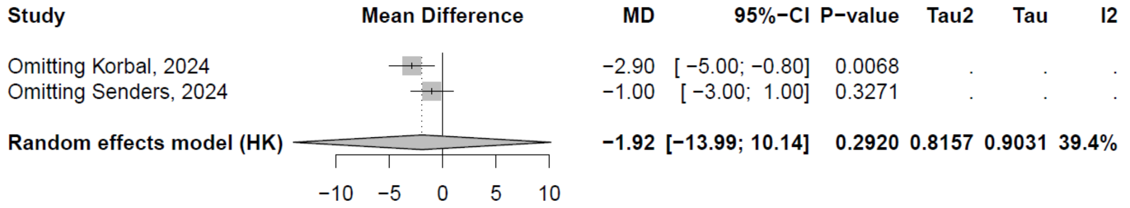

3

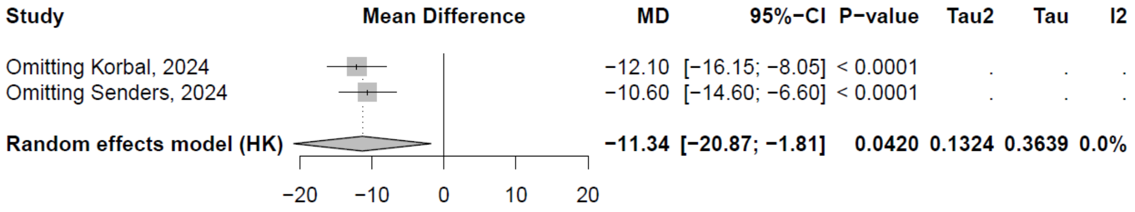

4

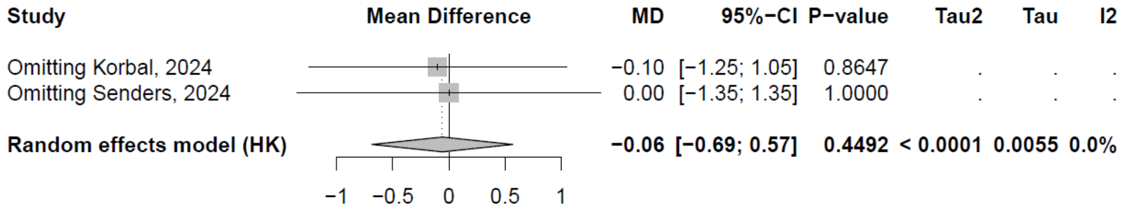

5

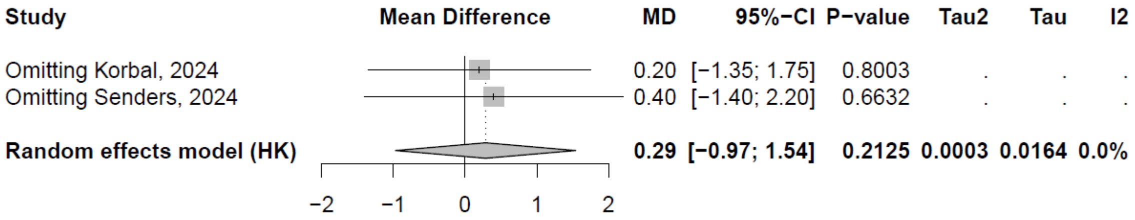

6A

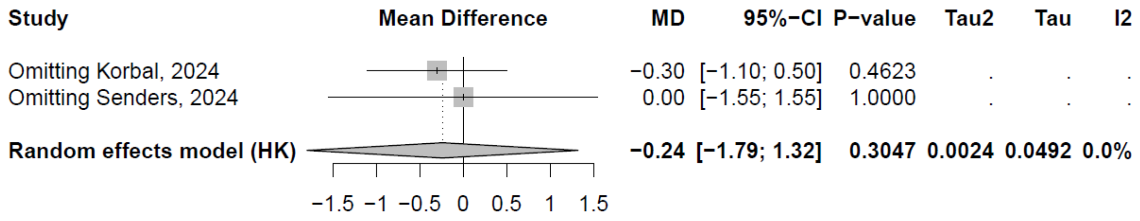

6B

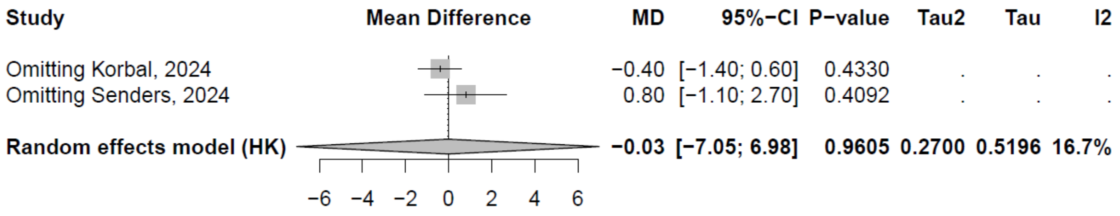

7F

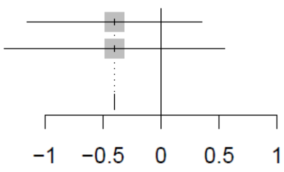

Study	MD	95%−CI	P−value	Tau2	Tau	I2
Omitting Korbal, 2024	−0.40	[−1.15; 0.35]	0.2959	.	.	.
Omitting Senders, 2024	−0.40	[−1.35; 0.55]	0.4092	.	.	.
**Random effects model (HK)**	**−0.40**	**[−0.40; −0.40]**	**0**	**0**	**0**	**0.0%**

9V

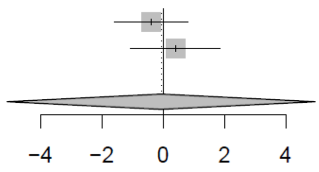

Study	MD	95%−CI	P−value	Tau2	Tau	I2
Omitting Korbal, 2024	−0.40	[−1.60; 0.80]	0.5135	.	.	.
Omitting Senders, 2024	0.40	[−1.05; 1.85]	0.5887	.	.	.
**Random effects model (HK)**	**−0.06**	**[−5.08; 4.95]**	**0.8986**	**0.0824**	**0.2871**	**0.0%**

14

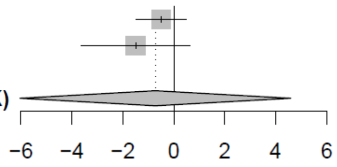

Study	MD	95%−CI	P−value	Tau2	Tau	I2
Omitting Korbal, 2024	−0.50	[−1.50; 0.50]	0.3271	.	.	.
Omitting Senders, 2024	−1.50	[−3.65; 0.65]	0.1715	.	.	.
**Random effects model (HK)**	**−0.73**	**[−6.04; 4.59]**	**0.3327**	**0.1273**	**0.3568**	**0.0%**

18C

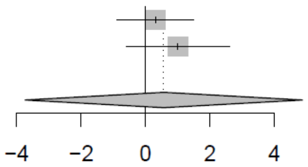

Study	MD	95%−CI	P−value	Tau2	Tau	I2
Omitting Korbal, 2024	0.30	[−0.90; 1.50]	0.6241	.	.	.
Omitting Senders, 2024	1.00	[−0.60; 2.60]	0.2206	.	.	.
**Random effects model (HK)**	**0.56**	**[−3.74; 4.86]**	**0.3459**	**0.0467**	**0.2160**	**0.0%**

19A

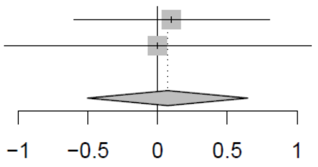

Study	MD	95%−CI	P−value	Tau2	Tau	I2
Omitting Korbal, 2024	0.10	[−0.60; 0.80]	0.7795	.	.	.
Omitting Senders, 2024	0.00	[−1.10; 1.10]	1.0000	.	.	.
**Random effects model (HK)**	**0.07**	**[−0.50; 0.65]**	**0.3608**	**< 0.0001**	**0.0075**	**0.0%**

19F

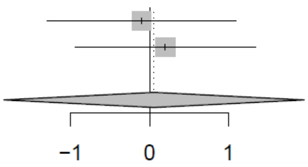

Study	MD	95%−CI	P−value	Tau2	Tau	I2
Omitting Korbal, 2024	−0.10	[−1.30; 1.10]	0.8703	.	.	.
Omitting Senders, 2024	0.20	[−0.95; 1.35]	0.7332	.	.	.
**Random effects model (HK)**	**0.06**	**[−1.85; 1.96]**	**0.7711**	**0.0027**	**0.0515**	**0.0%**

23F

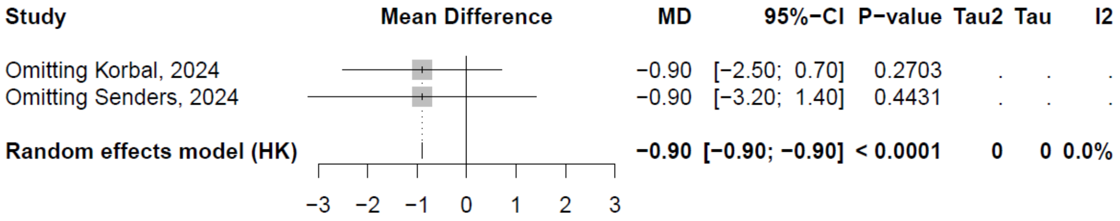

8

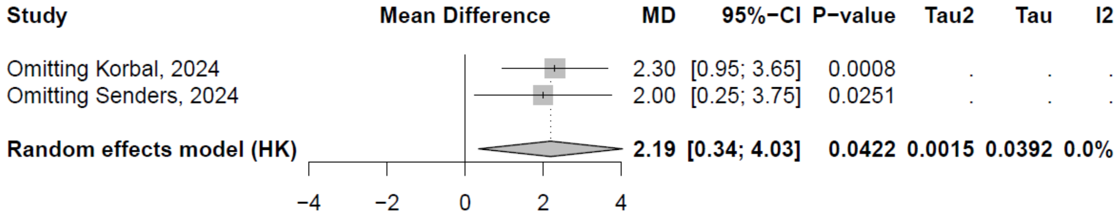

10A

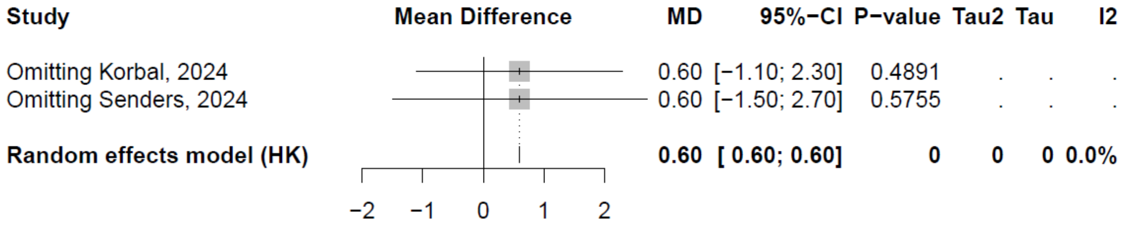

11A

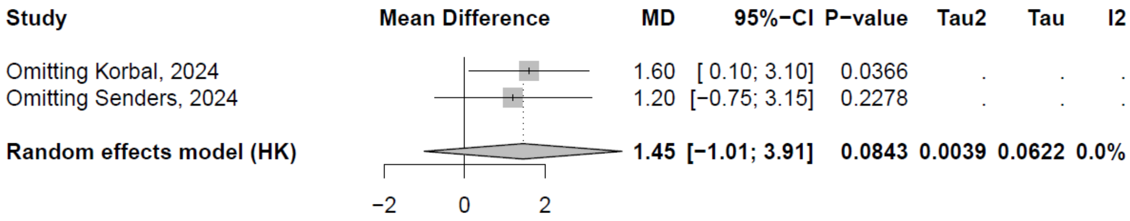

12F

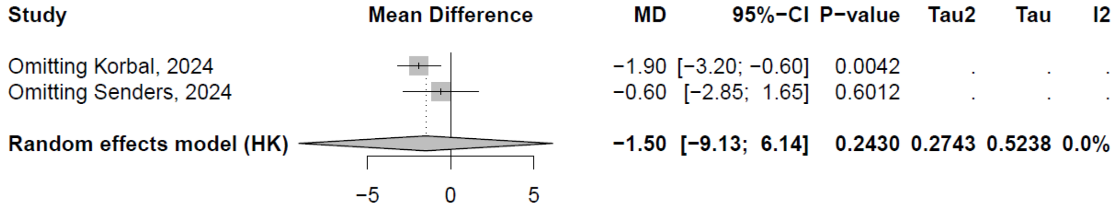

15B

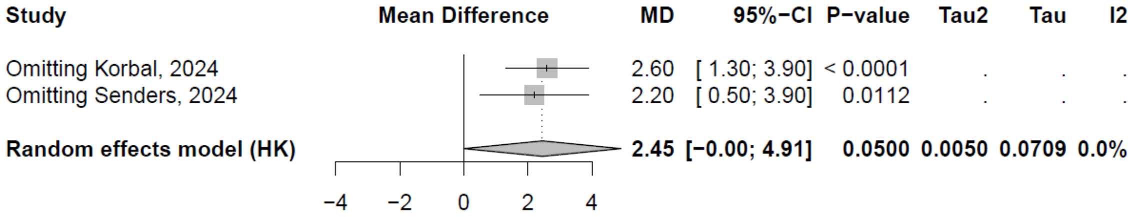

22F

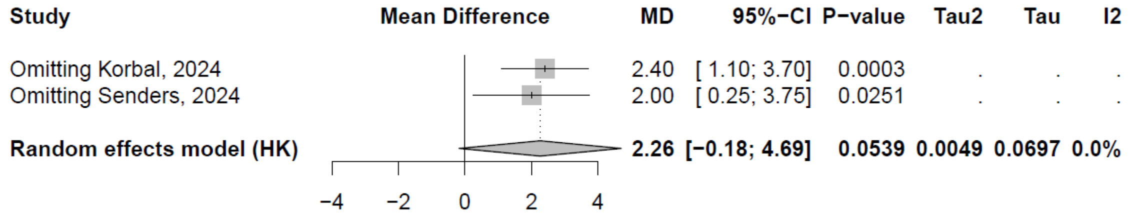

Study
Mean Difference
MD
95%−CI
P−value
Tau2
Tau
I2
Omitting Korbal, 2024
2.40 [1.10; 3.70] 0.0003 . . .
Omitting Senders, 2024
2.00 [0.25; 3.75] 0.0251 . . .
Random effects model (HK)
2.26 [−0.18; 4.69] 0.0539 0.0049 0.0697 0.0%
−4 −2 0 2 4

33F

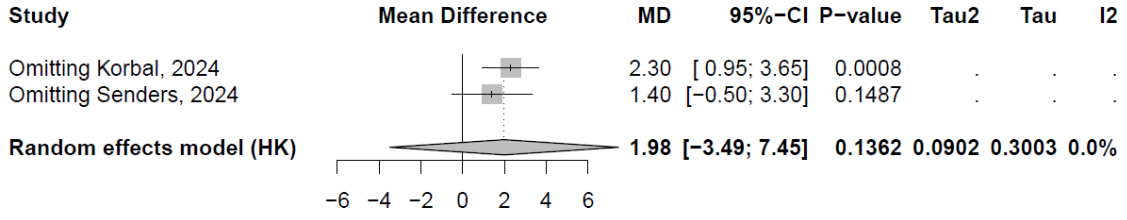

Study
Mean Difference
MD
95%−CI
P−value
Tau2
Tau
I2
Omitting Korbal, 2024
2.30 [0.95; 3.65] 0.0008 . . .
Omitting Senders, 2024
1.40 [−0.50; 3.30] 0.1487 . . .
Random effects model (HK)
1.98 [−3.49; 7.45] 0.1362 0.0902 0.3003 0.0%
−6 −4 −2 0 2 4 6

Figure S11. Sensivity analysis of GMTs of OPA PCV20 after primary series.

1

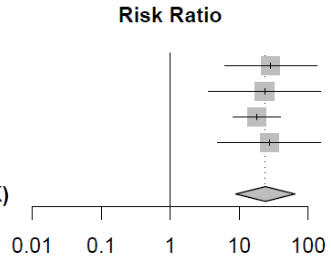

Study	RR	95%-CI	P-value	Tau2	Tau	I2
Omitting Korbal, 2024	28.78	[6.12; 135.30]	0.0113	0.3687	0.6072	96.5%
Omitting Senders, 2024	23.34	[3.53; 154.24]	0.0189	0.5615	0.7494	98.2%
Omitting Ishihara, 2024	17.99	[8.06; 40.15]	0.0041	0.0901	0.3001	90.3%
Omitting Senders, 2021	27.22	[4.84; 153.13]	0.0144	0.4687	0.6846	98.2%
**Random effects model (HK)**	**23.96**	**[8.89; 64.61]**	**0.0020**	**0.3738**	**0.6114**	**97.4%**

3

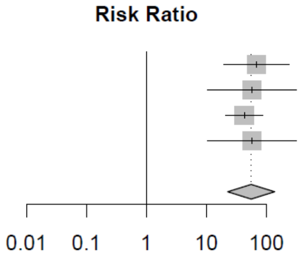

Study	RR	95%-CI	P-value	Tau2	Tau	I2
Omitting Korbal, 2024	67.82	[19.55; 235.23]	0.0047	0.2372	0.4870	97.0%
Omitting Senders, 2024	57.24	[10.35; 316.52]	0.0095	0.4611	0.6791	98.6%
Omitting Ishihara, 2024	42.68	[20.96; 86.89]	0.0019	0.0691	0.2629	89.9%
Omitting Senders, 2021	57.50	[10.49; 315.27]	0.0094	0.4594	0.6778	98.7%
**Random effects model (HK)**	**55.63**	**[22.62; 136.84]**	**0.0008**	**0.3088**	**0.5557**	**98.0%**

4

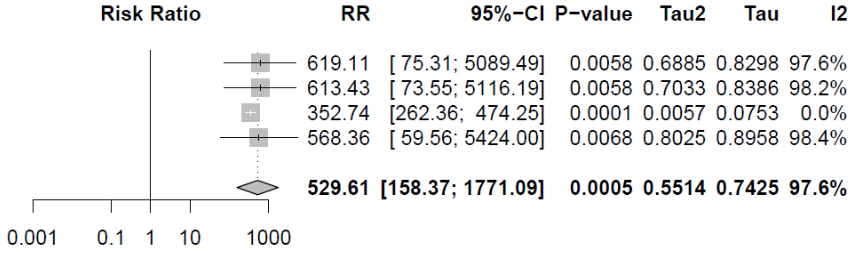

Study	RR	95%-CI	P-value	Tau2	Tau	I2
Omitting Korbal, 2024	619.11	[75.31; 5089.49]	0.0058	0.6885	0.8298	97.6%
Omitting Senders, 2024	613.43	[73.55; 5116.19]	0.0058	0.7033	0.8386	98.2%
Omitting Ishihara, 2024	352.74	[262.36; 474.25]	0.0001	0.0057	0.0753	0.0%
Omitting Senders, 2021	568.36	[59.56; 5424.00]	0.0068	0.8025	0.8958	98.4%
**Random effects model (HK)**	**529.61**	**[158.37; 1771.09]**	**0.0005**	**0.5514**	**0.7425**	**97.6%**

5

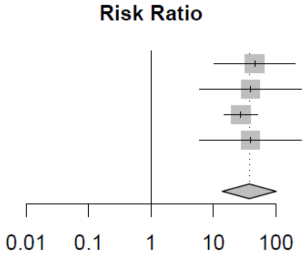

Study	RR	95%-CI	P-value	Tau2	Tau	I2
Omitting Korbal, 2024	45.67	[10.26; 203.35]	0.0082	0.3462	0.5884	97.5%
Omitting Senders, 2024	39.54	[6.06; 258.04]	0.0138	0.5575	0.7466	98.9%
Omitting Ishihara, 2024	27.53	[14.72; 51.49]	0.0019	0.0521	0.2283	89.3%
Omitting Senders, 2021	39.34	[6.03; 256.81]	0.0138	0.5604	0.7486	98.9%
**Random effects model (HK)**	**37.50**	**[13.85; 101.54]**	**0.0014**	**0.3806**	**0.6169**	**98.4%**

6A

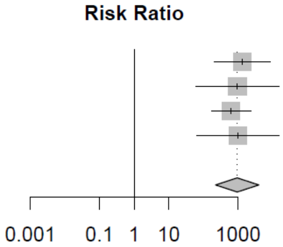

Study	RR	95%-CI	P-value	Tau2	Tau	I2
Omitting Korbal, 2024	1329.77	[208.65; 8474.81]	0.0036	0.5348	0.7313	97.9%
Omitting Senders, 2024	961.96	[61.15; 15132.87]	0.0086	1.2071	1.0987	99.0%
Omitting Ishihara, 2024	636.25	[170.49; 2374.38]	0.0022	0.2587	0.5086	95.2%
Omitting Senders, 2021	996.40	[64.78; 15326.72]	0.0084	1.1937	1.0926	99.0%
**Random effects model (HK)**	**949.33**	**[224.62; 4012.18]**	**0.0006**	**0.8016**	**0.8953**	**98.5%**

6B

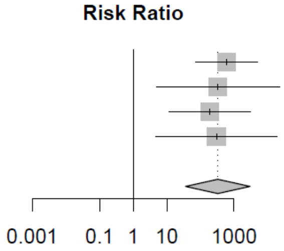

Study	RR	95%−CI	P−value	Tau2	Tau	I2
Omitting Korbal, 2024	602.14	[73.10; 4959.82]	0.0058	0.6855	0.8279	97.4%
Omitting Senders, 2024	331.58	[4.85; 22671.38]	0.0275	2.8580	1.6906	99.4%
Omitting Ishihara, 2024	194.02	[11.88; 3167.39]	0.0148	1.2256	1.1071	98.2%
Omitting Senders, 2021	299.49	[4.57; 19627.38]	0.0279	2.8085	1.6759	99.4%
**Random effects model (HK)**	**328.06**	**[35.97; 2992.36]**	**0.0036**	**1.9000**	**1.3784**	**99.1%**

7F

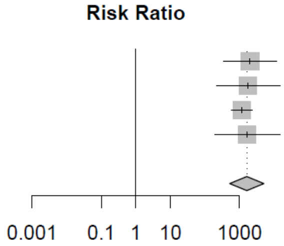

Study	RR	95%−CI	P−value	Tau2	Tau	I2
Omitting Korbal, 2024	2011.26	[350.36; 11545.86]	0.0028	0.4785	0.6917	97.9%
Omitting Senders, 2024	1784.93	[218.99; 14548.74]	0.0042	0.6983	0.8356	98.8%
Omitting Ishihara, 2024	1144.85	[575.62; 2276.98]	0.0005	0.0641	0.2532	88.0%
Omitting Senders, 2021	1673.48	[192.15; 14574.72]	0.0046	0.7465	0.8640	98.8%
**Random effects model (HK)**	**1622.59**	**[520.00; 5063.03]**	**0.0002**	**0.4984**	**0.7060**	**98.2%**

9V

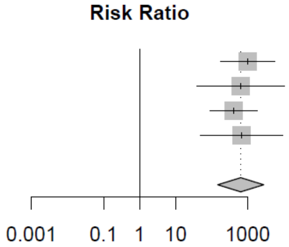

Study	RR	95%−CI	P−value	Tau2	Tau	I2
Omitting Korbal, 2024	1003.17	[177.91; 5656.35]	0.0034	0.3605	0.6004	93.7%
Omitting Senders, 2024	631.22	[38.00; 10485.72]	0.0101	1.1105	1.0538	98.7%
Omitting Ishihara, 2024	404.94	[89.94; 1823.18]	0.0034	0.2557	0.5057	92.4%
Omitting Senders, 2021	668.06	[48.15; 9269.91]	0.0087	1.0964	1.0471	98.8%
**Random effects model (HK)**	**642.97**	**[148.08; 2791.87]**	**0.0008**	**0.7353**	**0.8575**	**98.1%**

14

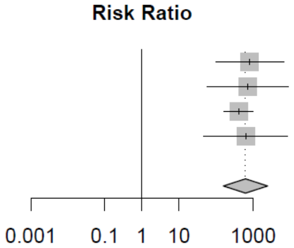

Study	RR	95%−CI	P−value	Tau2	Tau	I2
Omitting Korbal, 2024	808.42	[97.49; 6703.62]	0.0053	0.6854	0.8279	96.8%
Omitting Senders, 2024	715.11	[58.32; 8769.17]	0.0078	0.9760	0.9879	97.6%
Omitting Ishihara, 2024	408.19	[164.61; 1012.19]	0.0012	0.1037	0.3220	77.6%
Omitting Senders, 2021	628.82	[45.38; 8712.96]	0.0089	1.0871	1.0426	97.8%
**Random effects model (HK)**	**622.82**	**[156.77; 2474.29]**	**0.0007**	**0.7174**	**0.8470**	**96.7%**

18C

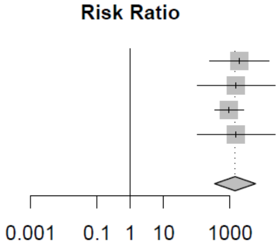

Study	RR	95%−CI	P−value	Tau2	Tau	I2
Omitting Korbal, 2024	1908.34	[247.70; 14702.47]	0.0039	0.6565	0.8103	98.3%
Omitting Senders, 2024	1522.98	[104.39; 22220.32]	0.0071	1.1453	1.0702	99.0%
Omitting Ishihara, 2024	936.78	[341.45; 2570.06]	0.0012	0.1454	0.3813	91.2%
Omitting Senders, 2021	1494.99	[100.98; 22133.32]	0.0073	1.1586	1.0764	99.0%
**Random effects model (HK)**	**1421.46**	**[343.57; 5881.01]**	**0.0005**	**0.7792**	**0.8827**	**98.6%**

19A

Study	Risk Ratio	RR	95%-CI	P-value	Tau2	Tau	I2
Omitting Korbal, 2024		185.92	[17.41; 1985.40]	0.0109	0.8753	0.9355	97.9%
Omitting Senders, 2024		149.67	[8.02; 2793.27]	0.0179	1.3575	1.1651	98.8%
Omitting Ishihara, 2024		85.56	[34.40; 212.82]	0.0023	0.1032	0.3212	82.1%
Omitting Senders, 2021		150.85	[8.17; 2783.72]	0.0178	1.3493	1.1616	98.8%
**Random effects model (HK)**		**138.15**	**[29.17; 654.17]**	**0.0021**	**0.9264**	**0.9625**	**98.3%**

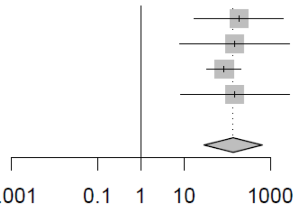

19F

Study	Risk Ratio	RR	95%-CI	P-value	Tau2	Tau	I2
Omitting Korbal, 2024		155.72	[13.12; 1848.65]	0.0127	0.9709	0.9854	98.9%
Omitting Senders, 2024		163.31	[15.29; 1744.78]	0.0115	0.8899	0.9434	98.9%
Omitting Ishihara, 2024		91.44	[75.23; 111.15]	0.0001	0.0013	0.0355	0.0%
Omitting Senders, 2021		158.63	[13.99; 1798.17]	0.0122	0.9412	0.9702	99.1%
**Random effects model (HK)**		**138.39**	**[35.96; 532.64]**	**0.0014**	**0.7006**	**0.8370**	**98.7%**

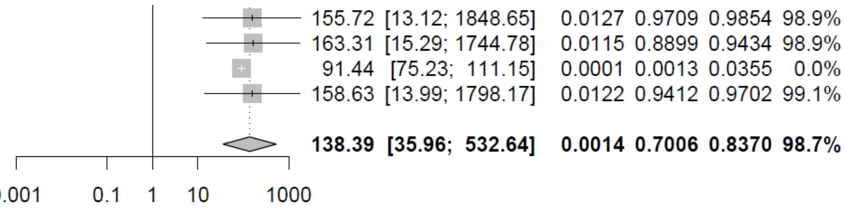

23F

Study	Risk Ratio	RR	95%-CI	P-value	Tau2	Tau	I2
Omitting Korbal, 2024		440.56	[35.63; 5448.06]	0.0091	0.9883	0.9941	98.0%
Omitting Senders, 2024		269.54	[5.18; 14012.95]	0.0259	2.4901	1.5780	99.1%
Omitting Ishihara, 2024		152.07	[19.73; 1172.17]	0.0088	0.6308	0.7942	95.4%
Omitting Senders, 2021		277.26	[5.39; 14254.49]	0.0255	2.4805	1.5750	99.1%
**Random effects model (HK)**		**266.20**	**[33.67; 2104.89]**	**0.0033**	**1.6537**	**1.2860**	**98.7%**

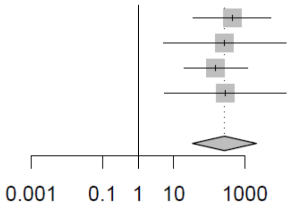

8

Study	Risk Ratio	RR	95%-CI	P-value	Tau2	Tau	I2
Omitting Korbal, 2024		792.02	[175.30; 3578.39]	0.0027	0.3411	0.5840	95.1%
Omitting Senders, 2024		493.16	[30.33; 8017.51]	0.0107	1.2366	1.1120	99.0%
Omitting Ishihara, 2024		370.90	[59.93; 2295.48]	0.0051	0.5124	0.7158	97.2%
Omitting Senders, 2021		551.24	[33.05; 9195.07]	0.0106	1.2619	1.1234	99.0%
**Random effects model (HK)**		**531.21**	**[120.99; 2332.35]**	**0.0009**	**0.8425**	**0.9179**	**98.5%**

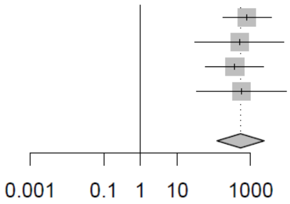

10A

Study	Risk Ratio	RR	95%-CI	P-value	Tau2	Tau	I2
Omitting Korbal, 2024		3199.13	[591.45; 17303.97]	0.0024	0.4331	0.6581	96.2%
Omitting Senders, 2024		2216.44	[164.08; 29939.58]	0.0061	1.0664	1.0327	98.3%
Omitting Ishihara, 2024		1596.87	[395.57; 6446.38]	0.0019	0.2794	0.5286	91.1%
Omitting Senders, 2021		2470.26	[188.18; 32427.45]	0.0058	1.0418	1.0207	98.1%
**Random effects model (HK)**		**2301.83**	**[586.28; 9037.33]**	**0.0004**	**0.7095**	**0.8423**	**97.4%**

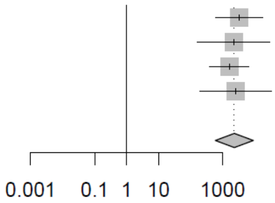

11A

Study	Risk Ratio	RR	95%−CI	P-value	Tau2	Tau	I2
Omitting Korbal, 2024		612.03	[53.63; 6984.74]	0.0077	0.9254	0.9620	98.0%
Omitting Senders, 2024		636.84	[62.53; 6485.68]	0.0069	0.8412	0.9172	98.1%
Omitting Ishihara, 2024		334.69	[214.53; 522.15]	0.0003	0.0165	0.1284	12.4%
Omitting Senders, 2021		559.43	[42.75; 7321.40]	0.0088	1.0448	1.0222	98.4%
**Random effects model (HK)**		**523.18**	**[133.21; 2054.86]**	**0.0007**	**0.7101**	**0.8427**	**97.7%**

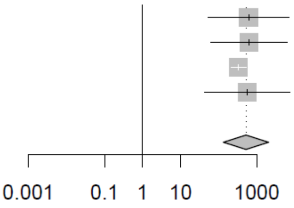

12F

Study	Risk Ratio	RR	95%−CI	P-value	Tau2	Tau	I2
Omitting Korbal, 2024		9882.05	[1893.77; 51566.26]	0.0017	0.4044	0.6359	92.4%
Omitting Senders, 2024		8450.80	[1153.71; 61901.41]	0.0026	0.6041	0.7772	95.1%
Omitting Ishihara, 2024		6049.39	[3335.67; 10970.83]	0.0003	0.0392	0.1979	71.8%
Omitting Senders, 2021		9122.21	[1371.40; 60678.62]	0.0023	0.5457	0.7387	95.0%
**Random effects model (HK)**		**8211.29**	**[2907.57; 23189.53]**	**0.0001**	**0.3955**	**0.6289**	**92.7%**

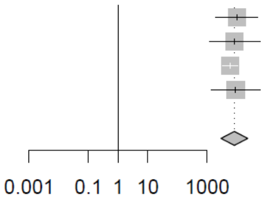

15B

Study	Risk Ratio	RR	95%−CI	P-value	Tau2	Tau	I2
Omitting Korbal, 2024		2146.10	[244.86; 18810.07]	0.0043	0.7132	0.8445	95.6%
Omitting Senders, 2024		1743.53	[131.61; 23098.04]	0.0064	1.0335	1.0166	97.3%
Omitting Ishihara, 2024		1124.38	[513.39; 2462.50]	0.0007	0.0630	0.2510	66.6%
Omitting Senders, 2021		1962.35	[173.85; 22150.60]	0.0055	0.9096	0.9537	97.0%
**Random effects model (HK)**		**1697.89**	**[437.73; 6585.90]**	**0.0004**	**0.6831**	**0.8265**	**95.9%**

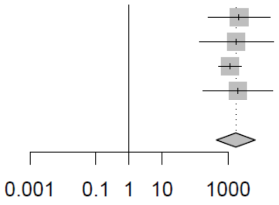

22F

Study	Risk Ratio	RR	95%−CI	P-value	Tau2	Tau	I2
Omitting Korbal, 2024		9440.67	[1989.83; 44790.97]	0.0016	0.3622	0.6018	95.0%
Omitting Senders, 2024		8141.57	[1204.84; 55015.54]	0.0024	0.5654	0.7519	97.6%
Omitting Ishihara, 2024		5617.14	[3113.96; 10132.52]	0.0003	0.0364	0.1909	72.7%
Omitting Senders, 2021		8331.21	[1275.00; 54438.65]	0.0023	0.5494	0.7412	97.6%
**Random effects model (HK)**		**7781.50**	**[2830.94; 21389.26]**	**< 0.0001**	**0.3803**	**0.6167**	**96.4%**

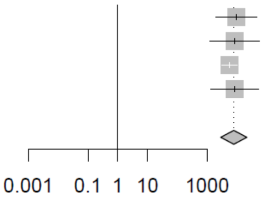

33F

Study	Risk Ratio	RR	95%−CI	P-value	Tau2	Tau	I2
Omitting Korbal, 2024		9647.12	[3114.48; 29881.98]	0.0008	0.1615	0.4018	83.6%
Omitting Senders, 2024		6487.93	[597.66; 70430.14]	0.0040	0.8796	0.9379	97.3%
Omitting Ishihara, 2024		4965.04	[972.08; 25359.74]	0.0020	0.3822	0.6182	92.8%
Omitting Senders, 2021		6521.75	[597.80; 71149.40]	0.0040	0.8815	0.9389	97.3%
**Random effects model (HK)**		**6692.72**	**[1902.78; 23540.57]**	**0.0002**	**0.5830**	**0.7635**	**95.9%**

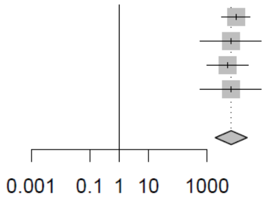

Figure S12. Sensivity analysis of GMTs of OPA PCV20 after booster dose.

1

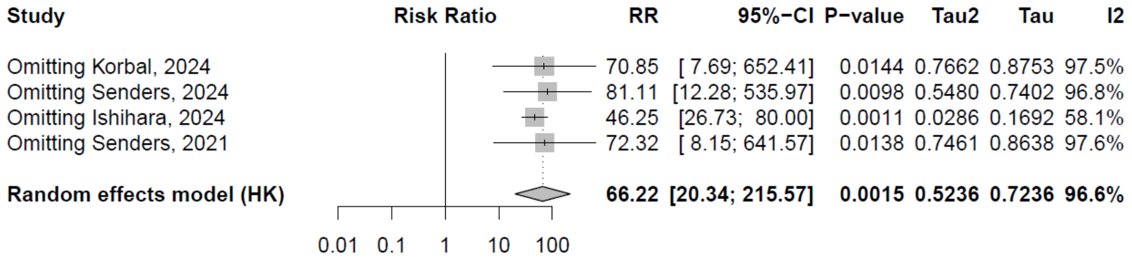

3

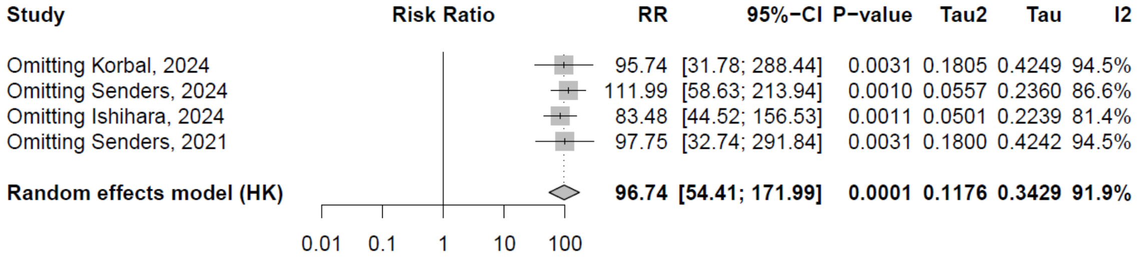

4

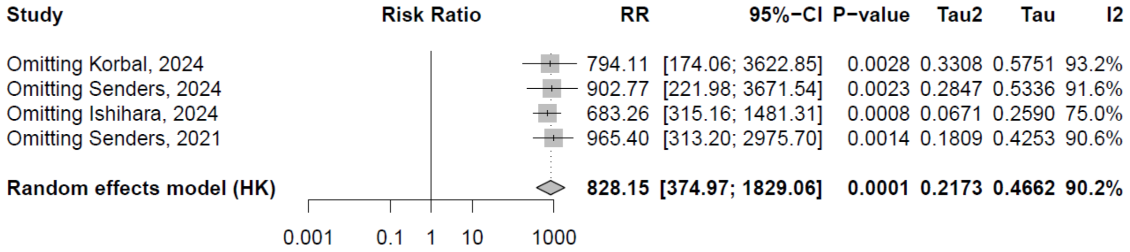

5

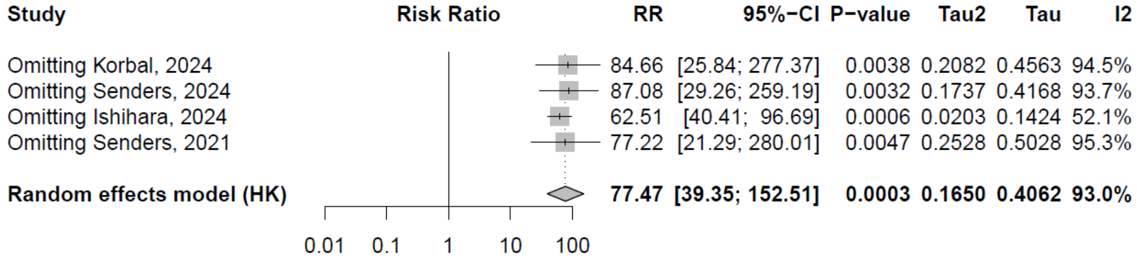

6A

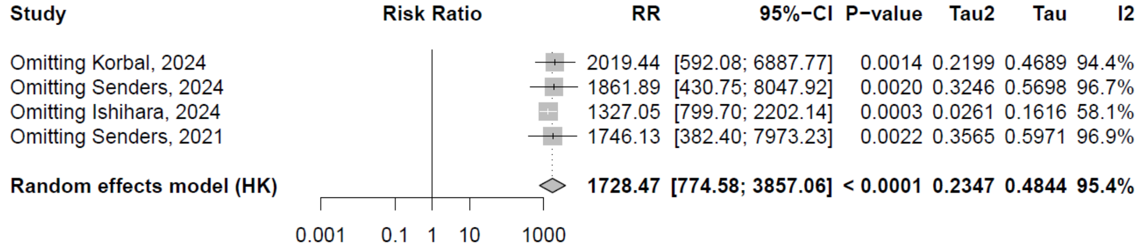

6B

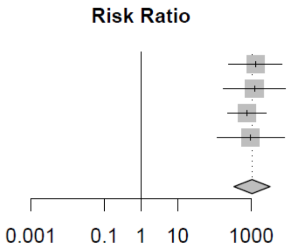

Study	RR	95%−CI	P−value	Tau2	Tau	I2
Omitting Korbal, 2024	1326.87	[249.06; 7069.04]	0.0029	0.4234	0.6507	95.6%
Omitting Senders, 2024	1230.90	[174.89; 8663.23]	0.0040	0.5891	0.7675	97.1%
Omitting Ishihara, 2024	782.31	[235.26; 2601.46]	0.0018	0.2033	0.4509	89.8%
Omitting Senders, 2021	972.89	[118.89; 7961.13]	0.0050	0.6886	0.8298	97.6%
**Random effects model (HK)**	**1057.04**	**[340.56; 3280.80]**	**0.0003**	**0.4803**	**0.6931**	**96.5%**

7F

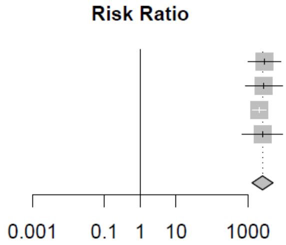

Study	RR	95%−CI	P−value	Tau2	Tau	I2
Omitting Korbal, 2024	2895.88	[990.12; 8469.80]	0.0010	0.1709	0.4134	93.3%
Omitting Senders, 2024	2787.47	[844.06; 9205.55]	0.0012	0.2168	0.4656	95.4%
Omitting Ishihara, 2024	2105.85	[1334.85; 3322.18]	0.0002	0.0247	0.1570	76.0%
Omitting Senders, 2021	2568.17	[705.76; 9345.23]	0.0015	0.2554	0.5054	95.6%
**Random effects model (HK)**	**2568.52**	**[1309.23; 5039.07]**	**< 0.0001**	**0.1668**	**0.4084**	**93.6%**

9V

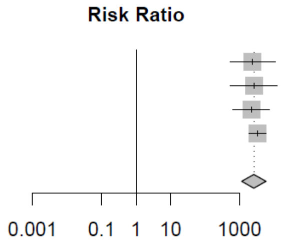

Study	RR	95%−CI	P−value	Tau2	Tau	I2
Omitting Korbal, 2024	2420.14	[544.60; 10754.82]	0.0020	0.3361	0.5798	95.8%
Omitting Senders, 2024	2606.75	[553.00; 12287.89]	0.0021	0.3688	0.6073	96.0%
Omitting Ishihara, 2024	2218.04	[657.86; 7478.38]	0.0013	0.2145	0.4631	93.0%
Omitting Senders, 2021	3353.82	[1870.51; 6013.38]	0.0003	0.0389	0.1973	70.8%
**Random effects model (HK)**	**2611.25**	**[1156.76; 5894.60]**	**< 0.0001**	**0.2413**	**0.4912**	**94.0%**

14

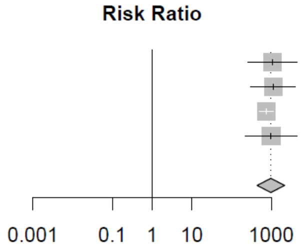

Study	RR	95%−CI	P−value	Tau2	Tau	I2
Omitting Korbal, 2024	1080.44	[264.94; 4406.15]	0.0022	0.2999	0.5476	96.0%
Omitting Senders, 2024	1115.02	[303.04; 4102.69]	0.0019	0.2572	0.5072	96.0%
Omitting Ishihara, 2024	765.39	[507.80; 1153.63]	0.0002	0.0160	0.1267	42.9%
Omitting Senders, 2021	996.93	[219.83; 4521.04]	0.0026	0.3531	0.5942	96.8%
**Random effects model (HK)**	**981.53**	**[443.23; 2173.59]**	**0.0001**	**0.2329**	**0.4826**	**95.3%**

18C

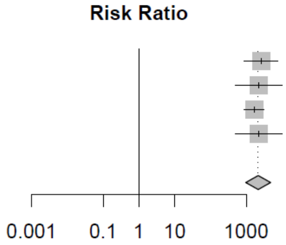

Study	RR	95%−CI	P−value	Tau2	Tau	I2
Omitting Korbal, 2024	2609.03	[869.11; 7832.22]	0.0011	0.1641	0.4051	90.9%
Omitting Senders, 2024	2227.60	[501.53; 9894.19]	0.0020	0.3412	0.5841	96.3%
Omitting Ishihara, 2024	1684.89	[883.35; 3213.74]	0.0004	0.0443	0.2104	76.1%
Omitting Senders, 2021	2220.25	[485.75; 10148.36]	0.0021	0.3426	0.5853	96.3%
**Random effects model (HK)**	**2167.13**	**[974.25; 4820.62]**	**< 0.0001**	**0.2267**	**0.4761**	**94.5%**

19A

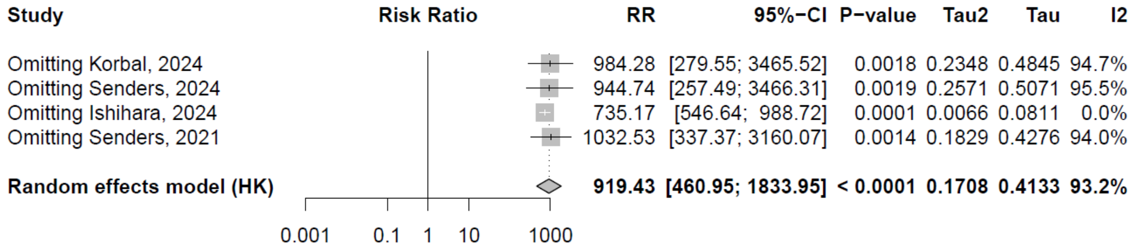

19F

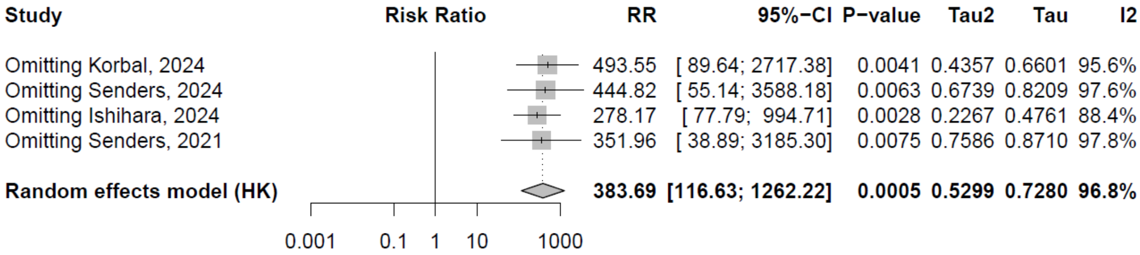

23F

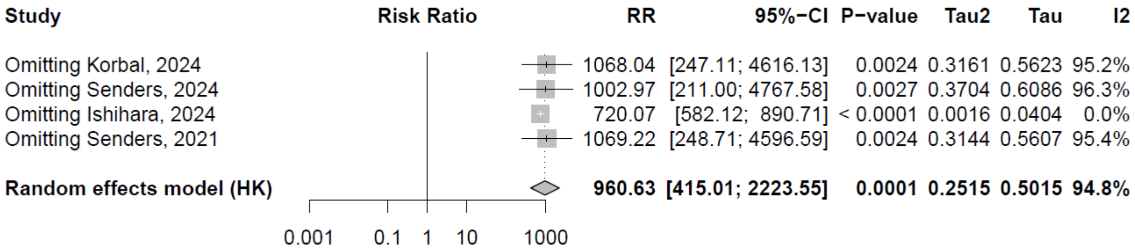

8

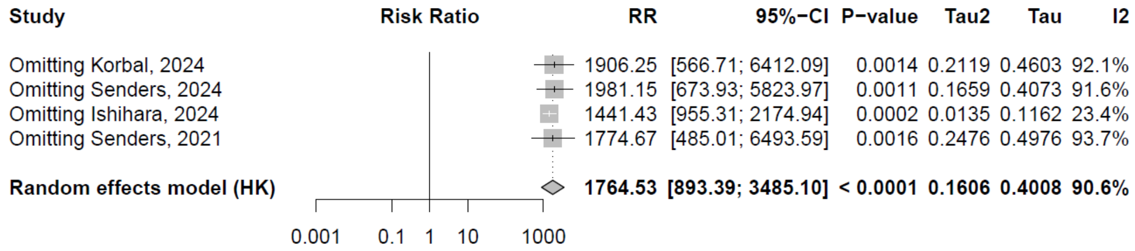

10A

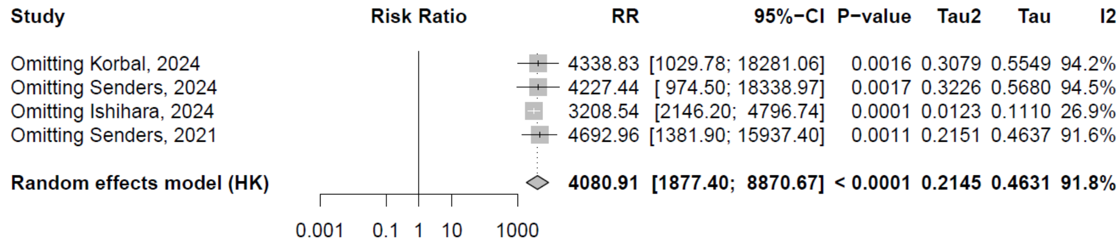

11A

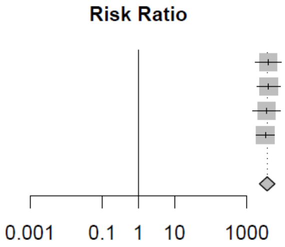

Study	RR	95%−CI	P−value	Tau2	Tau	I2
Omitting Korbal, 2024	3961.55	[1725.37; 9095.93]	0.0005	0.0863	0.2938	78.7%
Omitting Senders, 2024	4053.33	[1964.16; 8364.64]	0.0004	0.0622	0.2495	74.8%
Omitting Ishihara, 2024	3519.75	[1424.98; 8693.91]	0.0007	0.1048	0.3237	83.1%
Omitting Senders, 2021	3268.36	[1841.97; 5799.34]	0.0003	0.0321	0.1792	58.1%
**Random effects model (HK)**	**3687.87**	**[2255.19; 6030.69]**	**< 0.0001**	**0.0728**	**0.2699**	**76.4%**

12F

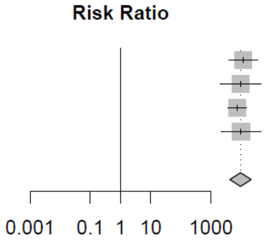

Study	RR	95%−CI	P−value	Tau2	Tau	I2
Omitting Korbal, 2024	11505.53	[3881.49; 34104.76]	0.0007	0.1610	0.4012	88.0%
Omitting Senders, 2024	9563.04	[2005.79; 45593.84]	0.0016	0.3676	0.6063	95.8%
Omitting Ishihara, 2024	7444.36	[3613.14; 15338.02]	0.0004	0.0621	0.2492	81.3%
Omitting Senders, 2021	9840.47	[2108.76; 45920.32]	0.0015	0.3605	0.6004	95.9%
**Random effects model (HK)**	**9486.26**	**[4182.79; 21514.14]**	**< 0.0001**	**0.2403**	**0.4902**	**93.8%**

15B

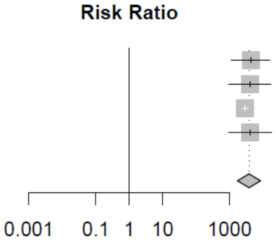

Study	RR	95%−CI	P−value	Tau2	Tau	I2
Omitting Korbal, 2024	4231.50	[1097.76; 16311.12]	0.0014	0.2682	0.5179	94.9%
Omitting Senders, 2024	4046.59	[952.10; 17198.65]	0.0016	0.3142	0.5606	95.7%
Omitting Ishihara, 2024	2929.94	[2407.86; 3565.23]	< 0.0001	0.0008	0.0289	0.0%
Omitting Senders, 2021	4033.61	[935.57; 17390.42]	0.0017	0.3175	0.5635	95.4%
**Random effects model (HK)**	**3787.35**	**[1706.91; 8403.51]**	**< 0.0001**	**0.2254**	**0.4747**	**94.4%**

22F

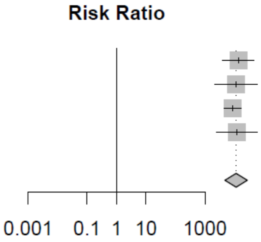

Study	RR	95%−CI	P−value	Tau2	Tau	I2
Omitting Korbal, 2024	13731.68	[3984.78; 47319.76]	0.0009	0.2219	0.4711	94.6%
Omitting Senders, 2024	11405.11	[2177.20; 59744.94]	0.0017	0.4199	0.6480	97.4%
Omitting Ishihara, 2024	8696.27	[4395.60; 17204.73]	0.0003	0.0481	0.2193	65.4%
Omitting Senders, 2021	12057.93	[2410.04; 60328.40]	0.0016	0.3965	0.6297	97.2%
**Random effects model (HK)**	**11376.09**	**[4749.45; 27248.52]**	**< 0.0001**	**0.2768**	**0.5261**	**96.4%**

33F

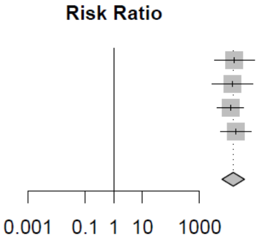

Study	RR	95%−CI	P−value	Tau2	Tau	I2
Omitting Korbal, 2024	16779.89	[3284.46; 85726.20]	0.0015	0.3850	0.6205	92.3%
Omitting Senders, 2024	14059.75	[2653.29; 74502.45]	0.0016	0.4070	0.6379	92.6%
Omitting Ishihara, 2024	12094.99	[4209.76; 34749.88]	0.0007	0.1422	0.3770	80.9%
Omitting Senders, 2021	18597.53	[5513.44; 62731.79]	0.0008	0.1982	0.4451	86.7%
**Random effects model (HK)**	**15180.25**	**[6124.22; 37627.67]**	**< 0.0001**	**0.2865**	**0.5353**	**89.8%**

Figure S13. Sensivity analysis of GMTs of OPA PCV13 after primary series.

1

Study	RR	95%-CI	P-value	Tau2	Tau	I2
Omitting Korbal, 2024	51.45	[7.35; 359.99]	0.0129	0.5913	0.7690	98.0%
Omitting Senders, 2024	45.05	[4.72; 430.37]	0.0185	0.8053	0.8974	98.8%
Omitting Ishihara, 2024	28.68	[16.82; 48.88]	0.0014	0.0301	0.1734	73.0%
Omitting Senders, 2021	46.21	[5.05; 423.04]	0.0175	0.7793	0.8828	98.8%
**Random effects model (HK)**	**42.01**	**[12.62; 139.82]**	**0.0022**	**0.5534**	**0.7439**	**98.2%**

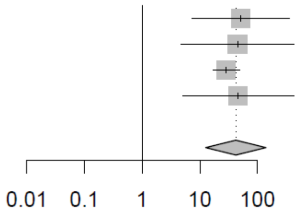

3

Study	RR	95%-CI	P-value	Tau2	Tau	I2
Omitting Korbal, 2024	87.42	[20.69; 369.29]	0.0056	0.3249	0.5700	98.0%
Omitting Senders, 2024	75.00	[11.83; 475.61]	0.0097	0.5421	0.7363	98.9%
Omitting Ishihara, 2024	53.55	[28.14; 101.91]	0.0014	0.0561	0.2368	88.3%
Omitting Senders, 2021	75.45	[11.99; 474.93]	0.0096	0.5387	0.7340	98.9%
**Random effects model (HK)**	**71.83**	**[27.04; 190.81]**	**0.0008**	**0.3671**	**0.6059**	**98.4%**

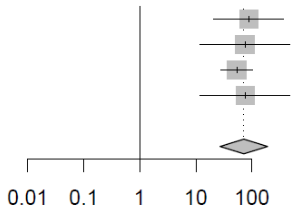

4

Study	RR	95%-CI	P-value	Tau2	Tau	I2
Omitting Korbal, 2024	583.10	[50.05; 6793.71]	0.0079	0.9344	0.9666	97.9%
Omitting Senders, 2024	652.47	[74.09; 5746.05]	0.0060	0.7283	0.8534	97.8%
Omitting Ishihara, 2024	348.14	[214.83; 564.19]	0.0004	0.0185	0.1359	39.0%
Omitting Senders, 2021	579.55	[50.57; 6642.14]	0.0078	0.9392	0.9691	98.3%
**Random effects model (HK)**	**527.81**	**[140.64; 1980.79]**	**0.0006**	**0.6578**	**0.8111**	**97.5%**

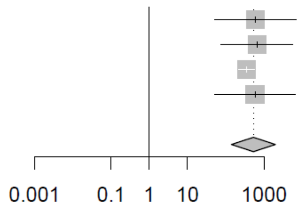

5

Study	RR	95%-CI	P-value	Tau2	Tau	I2
Omitting Korbal, 2024	64.89	[9.94; 423.43]	0.0107	0.5559	0.7456	98.5%
Omitting Senders, 2024	57.29	[6.19; 529.93]	0.0159	0.7896	0.8886	99.2%
Omitting Ishihara, 2024	35.88	[18.48; 69.67]	0.0019	0.0594	0.2438	88.2%
Omitting Senders, 2021	54.53	[5.57; 533.32]	0.0171	0.8318	0.9120	99.2%
**Random effects model (HK)**	**52.05**	**[15.62; 173.45]**	**0.0019**	**0.5609**	**0.7490**	**98.8%**

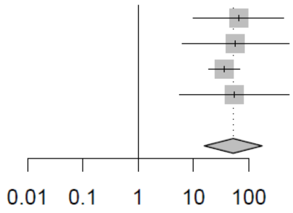

6A

Study	RR	95%-CI	P-value	Tau2	Tau	I2
Omitting Korbal, 2024	1396.77	[212.38; 9185.97]	0.0036	0.5589	0.7476	98.2%
Omitting Senders, 2024	1060.45	[75.37; 14921.09]	0.0077	1.1142	1.0556	99.0%
Omitting Ishihara, 2024	694.80	[223.81; 2156.97]	0.0016	0.1887	0.4344	92.8%
Omitting Senders, 2021	1087.88	[78.60; 15056.43]	0.0075	1.1007	1.0491	98.9%
**Random effects model (HK)**	**1028.76**	**[257.14; 4115.91]**	**0.0005**	**0.7430**	**0.8620**	**98.5%**

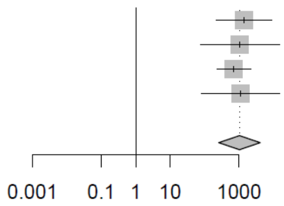

6B

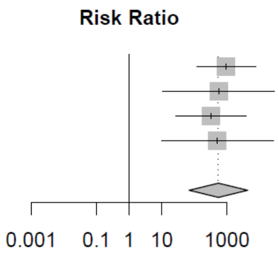

Study	RR	95%-CI	P-value	Tau2	Tau	I2
Omitting Korbal, 2024	953.99	[116.53; 7810.11]	0.0050	0.6910	0.8313	98.1%
Omitting Senders, 2024	560.67	[10.76; 29207.36]	0.0204	2.4995	1.5810	99.2%
Omitting Ishihara, 2024	331.23	[27.54; 3983.50]	0.0098	0.9671	0.9834	97.5%
Omitting Senders, 2021	514.38	[10.10; 26200.79]	0.0207	2.4767	1.5737	99.3%
**Random effects model (HK)**	**549.67**	**[69.52; 4346.33]**	**0.0023**	**1.6616**	**1.2890**	**98.9%**

7F

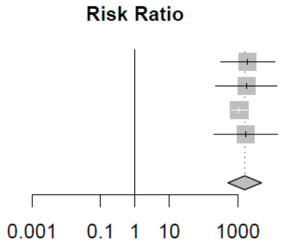

Study	RR	95%-CI	P-value	Tau2	Tau	I2
Omitting Korbal, 2024	1930.09	[312.45; 11922.52]	0.0031	0.5201	0.7211	98.4%
Omitting Senders, 2024	1769.25	[225.84; 13860.59]	0.0041	0.6730	0.8204	99.2%
Omitting Ishihara, 2024	1098.25	[634.87; 1899.85]	0.0003	0.0356	0.1887	77.9%
Omitting Senders, 2021	1658.88	[195.41; 14082.47]	0.0045	0.7309	0.8549	99.2%
**Random effects model (HK)**	**1589.29**	**[512.52; 4928.30]**	**0.0002**	**0.4929**	**0.7021**	**98.9%**

9V

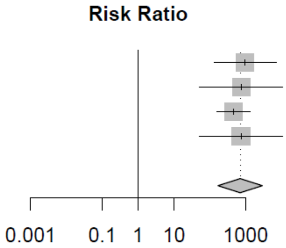

Study	RR	95%-CI	P-value	Tau2	Tau	I2
Omitting Korbal, 2024	961.41	[129.99; 7110.55]	0.0046	0.6135	0.7833	97.2%
Omitting Senders, 2024	744.90	[50.53; 10982.29]	0.0088	1.1464	1.0707	99.1%
Omitting Ishihara, 2024	456.81	[154.64; 1349.41]	0.0017	0.1558	0.3947	88.6%
Omitting Senders, 2021	741.33	[50.34; 10916.31]	0.0088	1.1494	1.0721	99.1%
**Random effects model (HK)**	**705.09**	**[169.79; 2928.03]**	**0.0007**	**0.7734**	**0.8794**	**98.6%**

14

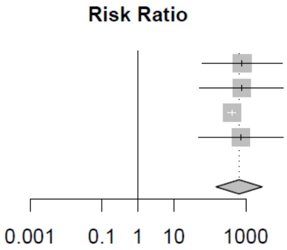

Study	RR	95%-CI	P-value	Tau2	Tau	I2
Omitting Korbal, 2024	795.00	[61.64; 10252.95]	0.0078	1.0264	1.0131	98.2%
Omitting Senders, 2024	756.23	[51.66; 11070.71]	0.0087	1.1301	1.0631	98.2%
Omitting Ishihara, 2024	407.17	[310.06; 534.68]	0.0001	0.0037	0.0604	0.0%
Omitting Senders, 2021	732.66	[47.86; 11216.26]	0.0091	1.1785	1.0856	98.5%
**Random effects model (HK)**	**652.61**	**[148.67; 2864.62]**	**0.0008**	**0.8344**	**0.9134**	**97.8%**

18C

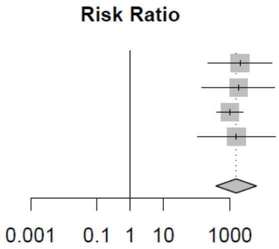

Study	RR	95%-CI	P-value	Tau2	Tau	I2
Omitting Korbal, 2024	2010.97	[224.94; 17977.98]	0.0045	0.7541	0.8684	98.5%
Omitting Senders, 2024	1795.61	[142.58; 22613.05]	0.0061	1.0213	1.0106	99.2%
Omitting Ishihara, 2024	1004.61	[405.97; 2486.05]	0.0009	0.1094	0.3307	86.1%
Omitting Senders, 2021	1569.76	[107.48; 22925.74]	0.0071	1.1491	1.0720	99.3%
**Random effects model (HK)**	**1551.85**	**[379.70; 6342.47]**	**0.0005**	**0.7641**	**0.8741**	**99.0%**

19A

Study	RR	95%−CI	P−value	Tau2	Tau	I2
Omitting Korbal, 2024	228.18	[20.50; 2539.44]	0.0105	0.9060	0.9518	98.1%
Omitting Senders, 2024	214.23	[16.14; 2843.99]	0.0123	1.0519	1.0256	98.6%
Omitting Ishihara, 2024	113.03	[57.65; 221.61]	0.0011	0.0472	0.2172	58.0%
Omitting Senders, 2021	189.39	[11.98; 2995.11]	0.0146	1.2086	1.0994	98.8%
**Random effects model (HK)**	**181.23**	**[42.18; 778.61]**	**0.0015**	**0.8097**	**0.8998**	**98.2%**

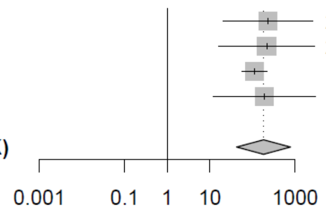

19F

Study	RR	95%−CI	P−value	Tau2	Tau	I2
Omitting Korbal, 2024	206.61	[19.04; 2242.22]	0.0106	0.8968	0.9470	98.3%
Omitting Senders, 2024	207.04	[19.22; 2230.73]	0.0106	0.8929	0.9449	98.4%
Omitting Ishihara, 2024	117.73	[111.67; 124.13]	< 0.0001	< 0.0001	0.0032	0.0%
Omitting Senders, 2021	203.87	[18.36; 2263.44]	0.0109	0.9179	0.9581	98.5%
**Random effects model (HK)**	**179.18**	**[47.56; 675.03]**	**0.0011**	**0.6744**	**0.8212**	**97.9%**

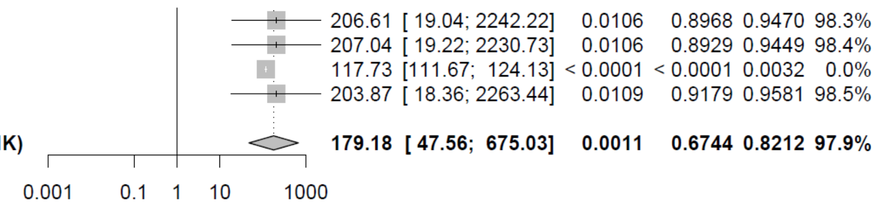

23F

Study	RR	95%−CI	P−value	Tau2	Tau	I2
Omitting Korbal, 2024	534.56	[35.34; 8084.76]	0.0100	1.1472	1.0711	98.2%
Omitting Senders, 2024	324.75	[5.19; 20306.74]	0.0265	2.7227	1.6501	99.3%
Omitting Ishihara, 2024	173.89	[22.35; 1352.72]	0.0084	0.6246	0.7903	95.1%
Omitting Senders, 2021	334.85	[5.48; 20462.77]	0.0260	2.7080	1.6456	99.3%
**Random effects model (HK)**	**317.62**	**[36.41; 2770.90]**	**0.0035**	**1.8110**	**1.3457**	**99.0%**

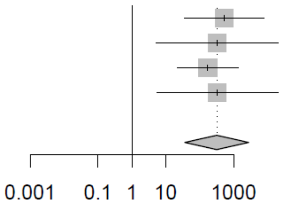

8

Study	RR	95%−CI	P−value	Tau2	Tau	I2
Omitting Korbal, 2024	17.09	[14.59; 20.02]	0.0002	0.0019	0.0442	44.5%
Omitting Senders, 2024	16.66	[15.06; 18.43]	< 0.0001	0.0005	0.0221	0.0%
Omitting Ishihara, 2024	17.50	[16.16; 18.96]	< 0.0001	0.0002	0.0139	0.0%
Omitting Senders, 2021	17.02	[14.69; 19.72]	0.0001	0.0019	0.0434	44.2%
**Random effects model (HK)**	**17.07**	**[15.72; 18.54]**	**< 0.0001**	**0.0011**	**0.0337**	**17.1%**

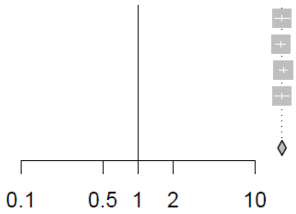

10A

Study	RR	95%−CI	P−value	Tau2	Tau	I2
Omitting Korbal, 2024	37.60	[33.94; 41.66]	< 0.0001	0.0004	0.0192	0.0%
Omitting Senders, 2024	38.51	[34.85; 42.56]	< 0.0001	0.0003	0.0169	0.0%
Omitting Ishihara, 2024	37.63	[34.75; 40.75]	< 0.0001	0.0001	0.0116	0.0%
Omitting Senders, 2021	38.34	[34.80; 42.23]	< 0.0001	0.0003	0.0164	0.0%
**Random effects model (HK)**	**38.00**	**[35.78; 40.36]**	**< 0.0001**	**0.0003**	**0.0168**	**0.0%**

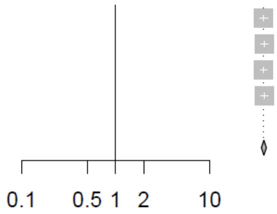

11A

Study	RR	95%-CI	P-value	Tau2	Tau	I2
Omitting Korbal, 2024	39.11	[9.41; 162.49]	0.0081	0.3090	0.5559	94.3%
Omitting Senders, 2024	38.86	[9.47; 159.52]	0.0079	0.3041	0.5515	94.2%
Omitting Ishihara, 2024	37.19	[10.27; 134.64]	0.0068	0.2532	0.5032	93.8%
Omitting Senders, 2021	50.53	[42.58; 59.97]	0.0001	0.0037	0.0605	21.5%
**Random effects model (HK)**	**41.62**	**[19.51; 88.78]**	**0.0006**	**0.2135**	**0.4621**	**91.4%**

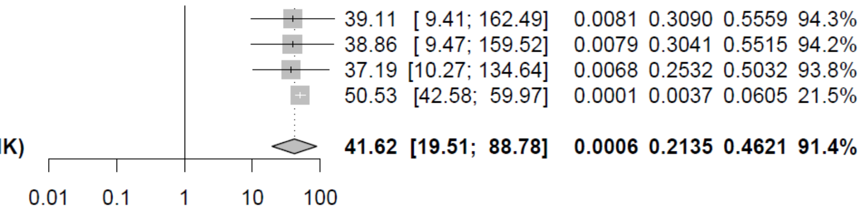

12F

Study	RR	95%-CI	P-value	Tau2	Tau	I2
Omitting Korbal, 2024	25.34	[20.79; 30.88]	0.0002	0.0037	0.0611	60.2%
Omitting Senders, 2024	24.80	[21.80; 28.20]	< 0.0001	0.0015	0.0384	48.7%
Omitting Ishihara, 2024	26.55	[24.28; 29.03]	< 0.0001	0.0002	0.0157	0.0%
Omitting Senders, 2021	25.30	[21.12; 30.31]	0.0002	0.0035	0.0592	65.7%
**Random effects model (HK)**	**25.32**	**[22.83; 28.09]**	**< 0.0001**	**0.0023**	**0.0483**	**58.5%**

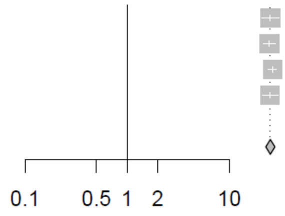

15B

Study	RR	95%-CI	P-value	Tau2	Tau	I2
Omitting Korbal, 2024	18.57	[13.11; 26.29]	0.0008	0.0129	0.1136	43.3%
Omitting Senders, 2024	18.17	[12.50; 26.43]	0.0009	0.0165	0.1284	50.7%
Omitting Ishihara, 2024	18.51	[13.10; 26.14]	0.0008	0.0132	0.1148	47.4%
Omitting Senders, 2021	17.24	[15.99; 18.59]	< 0.0001	0.0001	0.0110	0.0%
**Random effects model (HK)**	**18.01**	**[14.97; 21.67]**	**< 0.0001**	**0.0097**	**0.0987**	**27.6%**

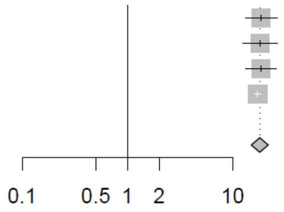

22F

Study	RR	95%-CI	P-value	Tau2	Tau	I2
Omitting Korbal, 2024	10.33	[5.59; 19.08]	0.0132	0.0011	0.0326	0.0%
Omitting Senders, 2024	10.12	[9.14; 11.22]	0.0001	0.0006	0.0247	0.0%
Omitting Ishihara, 2024	10.19	[6.10; 17.03]	0.0111	0.0012	0.0352	0.0%
Omitting Senders, 2021	10.00	[10.00; 10.00]	< 0.0001	0	0	0.0%
**Random effects model (HK)**	**10.12**	**[9.14; 11.22]**	**0.0001**	**0.0006**	**0.0247**	**0.0%**

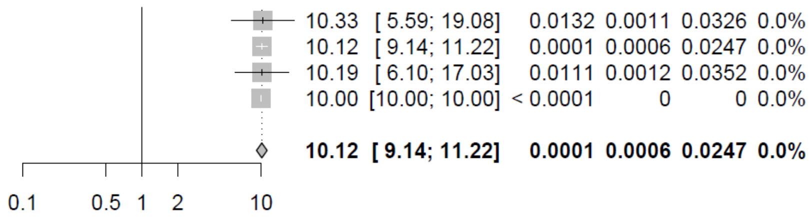

33F

Study	RR	95%-CI	P-value	Tau2	Tau	I2
Omitting Korbal, 2024	132.17	[26.96; 647.96]	0.0057	0.3819	0.6180	92.8%
Omitting Senders, 2024	127.81	[28.39; 575.41]	0.0052	0.3402	0.5832	91.5%
Omitting Ishihara, 2024	132.42	[26.92; 651.34]	0.0057	0.3837	0.6195	92.7%
Omitting Senders, 2021	183.38	[157.53; 213.47]	< 0.0001	0.0019	0.0440	29.1%
**Random effects model (HK)**	**143.88**	**[61.42; 337.07]**	**0.0003**	**0.2684**	**0.5181**	**89.2%**

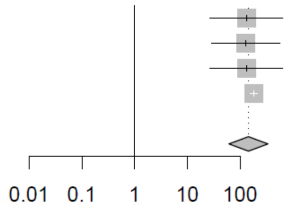

Figure S14. Sensivity analysis of GMTs of OPA PCV13 after booster dose.

1

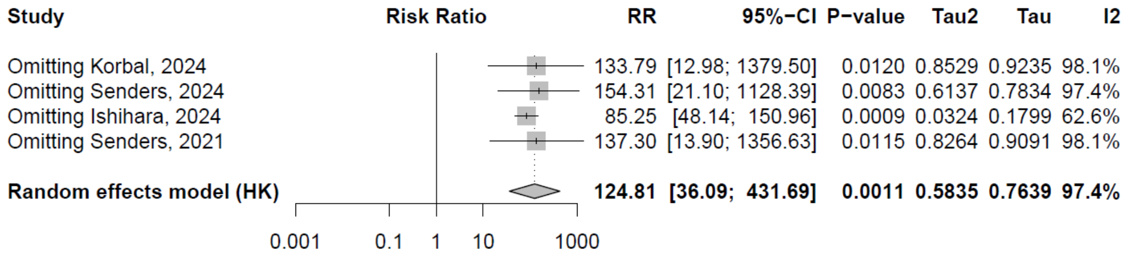

3

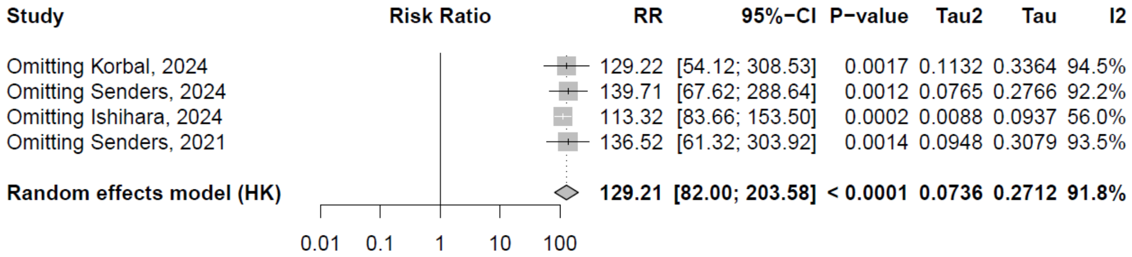

4

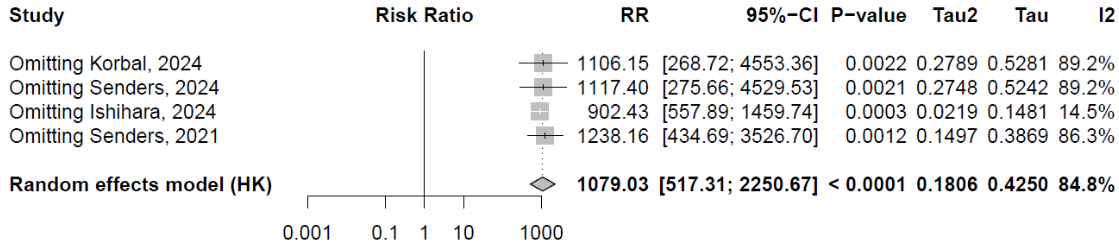

5

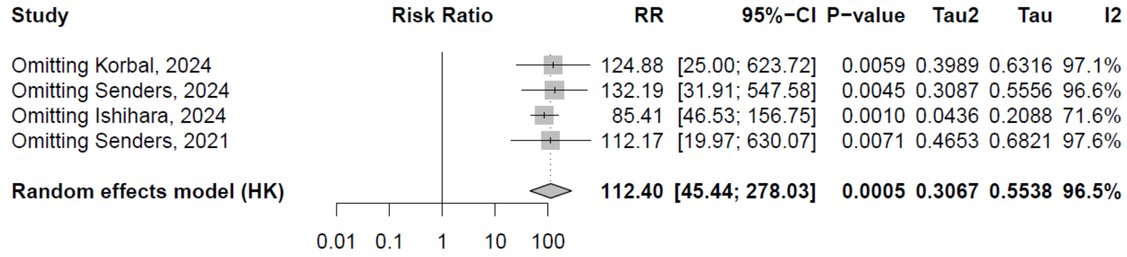

6A

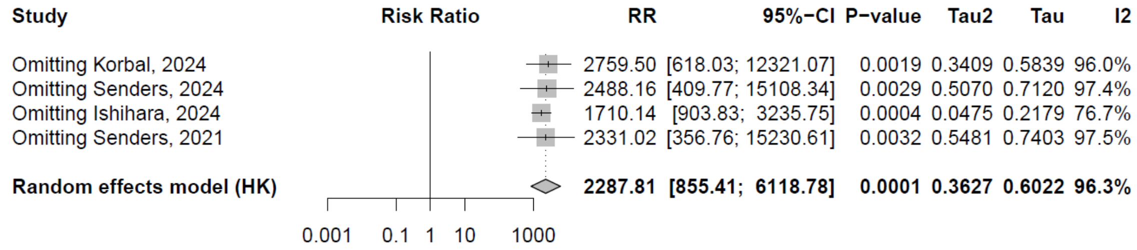

6B

Study	RR	95%-CI	P-value	Tau2	Tau	I2
Omitting Korbal, 2024	2137.45	[420.71; 10859.47]	0.0024	0.3954	0.6288	95.5%
Omitting Senders, 2024	1895.06	[253.90; 14144.34]	0.0038	0.6271	0.7919	97.7%
Omitting Ishihara, 2024	1220.32	[485.36; 3068.23]	0.0009	0.1067	0.3266	81.6%
Omitting Senders, 2021	1660.58	[200.26; 13769.83]	0.0044	0.6981	0.8355	97.9%
**Random effects model (HK)**	**1698.38**	**[557.69; 5172.21]**	**0.0002**	**0.4623**	**0.6799**	**96.9%**

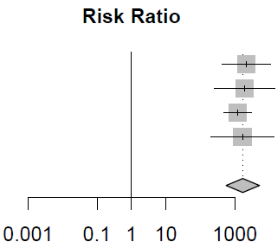

7F

Study	RR	95%-CI	P-value	Tau2	Tau	I2
Omitting Korbal, 2024	3514.51	[877.62; 14074.24]	0.0016	0.2940	0.5422	96.2%
Omitting Senders, 2024	3560.33	[936.17; 13540.24]	0.0014	0.2731	0.5226	96.5%
Omitting Ishihara, 2024	2442.69	[1348.65; 4424.22]	0.0003	0.0459	0.2143	79.1%
Omitting Senders, 2021	3070.32	[649.49; 14514.28]	0.0020	0.3762	0.6133	96.9%
**Random effects model (HK)**	**3115.62**	**[1379.02; 7039.10]**	**< 0.0001**	**0.2484**	**0.4984**	**95.6%**

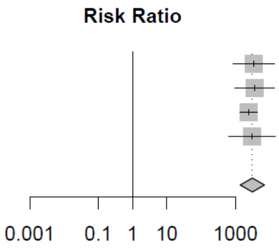

9V

Study	RR	95%-CI	P-value	Tau2	Tau	I2
Omitting Korbal, 2024	3274.50	[1028.40; 10426.26]	0.0011	0.1948	0.4414	92.9%
Omitting Senders, 2024	3675.53	[1060.69; 12736.55]	0.0012	0.2283	0.4778	92.1%
Omitting Ishihara, 2024	3135.63	[1155.84; 8506.50]	0.0008	0.1381	0.3716	88.4%
Omitting Senders, 2021	4273.29	[2338.40; 7809.17]	0.0003	0.0456	0.2136	78.9%
**Random effects model (HK)**	**3570.38**	**[1851.41; 6885.35]**	**< 0.0001**	**0.1520**	**0.3898**	**89.9%**

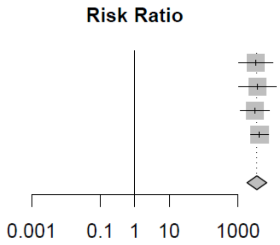

14

Study	RR	95%-CI	P-value	Tau2	Tau	I2
Omitting Korbal, 2024	1047.27	[277.05; 3958.70]	0.0020	0.2637	0.5135	95.1%
Omitting Senders, 2024	1216.13	[552.27; 2678.01]	0.0007	0.0824	0.2871	88.1%
Omitting Ishihara, 2024	847.77	[376.74; 1907.70]	0.0008	0.0861	0.2934	83.2%
Omitting Senders, 2021	981.36	[263.05; 3661.19]	0.0020	0.2626	0.5124	95.3%
**Random effects model (HK)**	**1015.64**	**[503.57; 2048.40]**	**< 0.0001**	**0.1757**	**0.4192**	**92.9%**

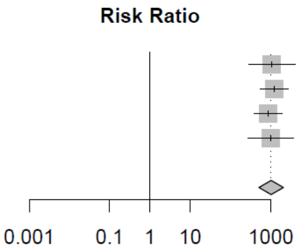

18C

Study	RR	95%-CI	P-value	Tau2	Tau	I2
Omitting Korbal, 2024	3476.24	[938.84; 12871.47]	0.0014	0.2548	0.5048	94.9%
Omitting Senders, 2024	3179.62	[669.59; 15098.82]	0.0020	0.3707	0.6088	96.6%
Omitting Ishihara, 2024	2270.38	[1420.24; 3629.39]	0.0002	0.0208	0.1442	52.9%
Omitting Senders, 2021	3062.26	[622.35; 15067.70]	0.0021	0.3924	0.6264	96.9%
**Random effects model (HK)**	**2972.14**	**[1278.30; 6910.46]**	**< 0.0001**	**0.2615**	**0.5114**	**95.4%**

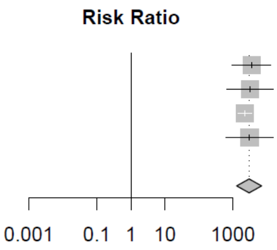

19A

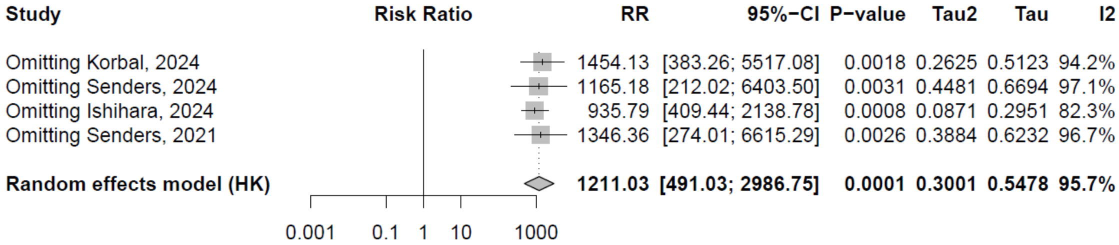

19F

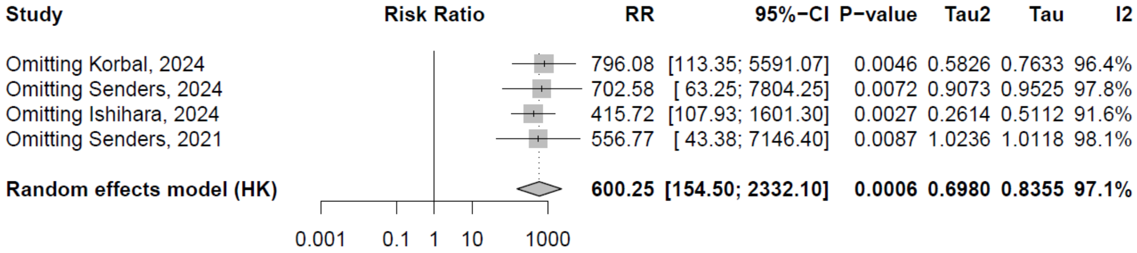

23F

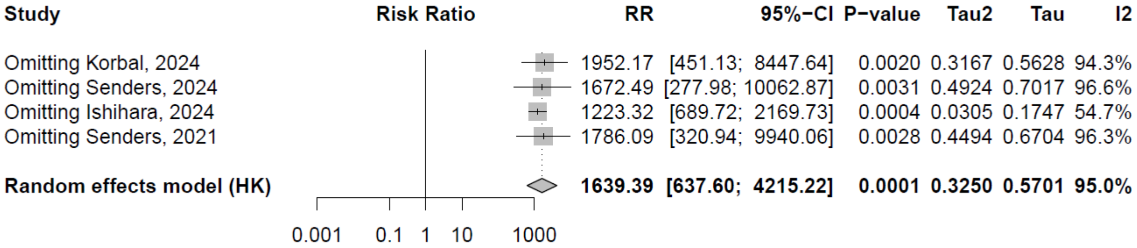

8

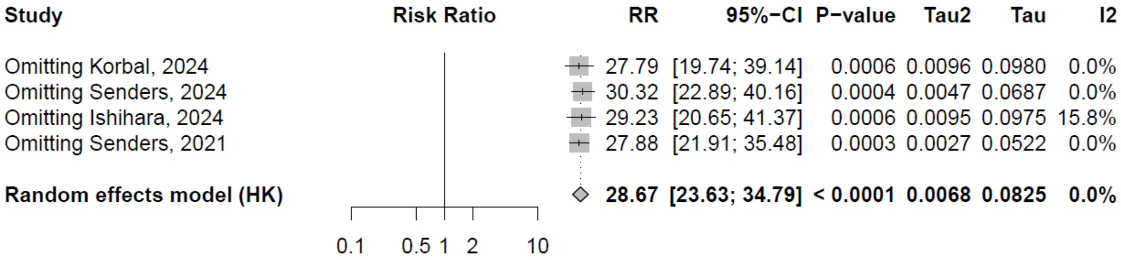

10A

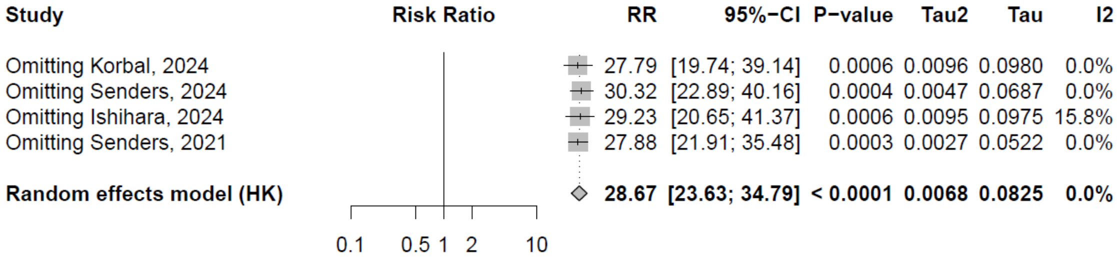

11A

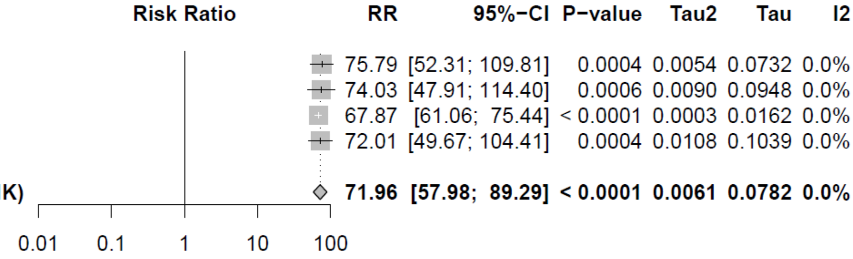

Study	RR	95%-CI	P-value	Tau2	Tau	I2
Omitting Korbal, 2024	75.79	[52.31; 109.81]	0.0004	0.0054	0.0732	0.0%
Omitting Senders, 2024	74.03	[47.91; 114.40]	0.0006	0.0090	0.0948	0.0%
Omitting Ishihara, 2024	67.87	[61.06; 75.44]	< 0.0001	0.0003	0.0162	0.0%
Omitting Senders, 2021	72.01	[49.67; 104.41]	0.0004	0.0108	0.1039	0.0%
**Random effects model (HK)**	**71.96**	**[57.98; 89.29]**	**< 0.0001**	**0.0061**	**0.0782**	**0.0%**

12F

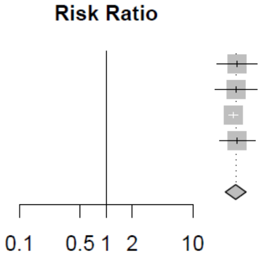

Study	RR	95%-CI	P-value	Tau2	Tau	I2
Omitting Korbal, 2024	32.41	[19.20; 54.71]	0.0012	0.0316	0.1778	58.7%
Omitting Senders, 2024	31.66	[18.16; 55.19]	0.0014	0.0368	0.1919	61.9%
Omitting Ishihara, 2024	29.36	[25.73; 33.50]	< 0.0001	0.0005	0.0232	0.0%
Omitting Senders, 2021	32.65	[20.30; 52.50]	0.0010	0.0260	0.1614	54.1%
**Random effects model (HK)**	**31.25**	**[23.73; 41.14]**	**< 0.0001**	**0.0221**	**0.1486**	**43.5%**

15B

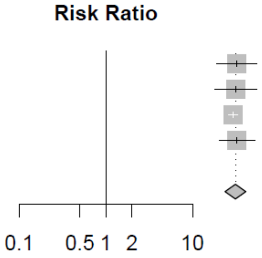

Study	RR	95%-CI	P-value	Tau2	Tau	I2
Omitting Korbal, 2024	32.41	[19.20; 54.71]	0.0012	0.0316	0.1778	58.7%
Omitting Senders, 2024	31.66	[18.16; 55.19]	0.0014	0.0368	0.1919	61.9%
Omitting Ishihara, 2024	29.36	[25.73; 33.50]	< 0.0001	0.0005	0.0232	0.0%
Omitting Senders, 2021	32.65	[20.30; 52.50]	0.0010	0.0260	0.1614	54.1%
**Random effects model (HK)**	**31.25**	**[23.73; 41.14]**	**< 0.0001**	**0.0221**	**0.1486**	**43.5%**

22F

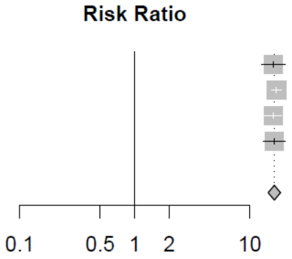

Study	RR	95%-CI	P-value	Tau2	Tau	I2
Omitting Korbal, 2024	16.14	[12.79; 20.35]	0.0004	0.0012	0.0350	0.0%
Omitting Senders, 2024	17.13	[15.52; 18.91]	< 0.0001	< 0.0001	0.0076	0.0%
Omitting Ishihara, 2024	16.06	[13.50; 19.11]	0.0002	0.0004	0.0211	0.0%
Omitting Senders, 2021	16.38	[13.05; 20.56]	0.0004	0.0014	0.0379	0.0%
**Random effects model (HK)**	**16.40**	**[14.48; 18.57]**	**< 0.0001**	**0.0008**	**0.0274**	**0.0%**

33F

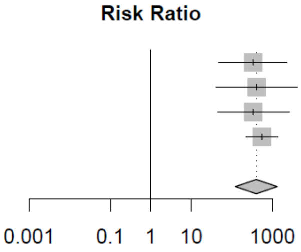

Study	RR	95%-CI	P-value	Tau2	Tau	I2
Omitting Korbal, 2024	327.09	[46.47; 2302.23]	0.0061	0.5611	0.7491	91.9%
Omitting Senders, 2024	408.31	[41.27; 4039.78]	0.0078	0.7894	0.8885	93.1%
Omitting Ishihara, 2024	335.67	[43.69; 2579.00]	0.0066	0.6206	0.7878	93.9%
Omitting Senders, 2021	547.74	[217.84; 1377.22]	0.0012	0.1149	0.3389	88.8%
**Random effects model (HK)**	**399.53**	**[121.78; 1310.71]**	**0.0005**	**0.5148**	**0.7175**	**92.2%**
